# The Great Genotyper: a graph-based method for population genotyping of small and structural variants

**DOI:** 10.1093/gigascience/giaf112

**Published:** 2025-10-03

**Authors:** Moustafa Shokrof, Mohamed Abuelanin, C Titus Brown, Tamer A Mansour

**Affiliations:** Department of Population Health and Reproduction, School of Veterinary Medicine, University of California, Davis, CA 95616, USA; Department of Computer Science, The Graduate Group in Computer Science (GGCS), University of California, Davis, CA 95616, USA; Department of Population Health and Reproduction, School of Veterinary Medicine, University of California, Davis, CA 95616, USA; Department of Computer Science, The Graduate Group in Computer Science (GGCS), University of California, Davis, CA 95616, USA; Department of Population Health and Reproduction, School of Veterinary Medicine, University of California, Davis, CA 95616, USA; Department of Population Health and Reproduction, School of Veterinary Medicine, University of California, Davis, CA 95616, USA; Department of Clinical Pathology, School of Medicine, Mansoura University, Mansoura 35516, Egypt

**Keywords:** genotyping, structural variants, allele frequency, pangenome, counting colored de Bruijn graph

## Abstract

**Background:**

Long-read sequencing (LRS) enables high-quality structural variant (SV) discovery. SV genotypers utilize these precise call sets to improve the recall and precision of genotyping in short-read sequencing (SRS) samples. With the extensive growth in publicly available SRS datasets, it is now possible to calculate accurate population allele frequencies of SVs. However, reprocessing hundreds of terabytes of raw SRS data to genotype new variants is impractical for population-scale studies, a computational challenge known as the N+1 problem (i.e., the challenge of re-genotyping an entire cohort for one additional variant). Overcoming this computational bottleneck is essential for analyzing new SVs from the growing number of pangenomes, public genomic databases, and pathogenic variant discovery studies.

**Results:**

We propose the Great Genotyper, a population-scale genotyping workflow to address the N+1 problem. Applied to a human dataset, the workflow begins by preprocessing 4.2k short-read samples of a total of 183 TB raw data to create an 867-GB Counting Colored de Bruijn Graph (CCDG). The Great Genotyper uses this CCDG to genotype a list of phased or unphased variants, leveraging the CCDG population information to increase both precision and recall. The Great Genotyper offers the same accuracy as the state-of-the-art genotypers while achieving unprecedented performance. It took about 100 hours to genotype 4.5M variants across the 4.2k samples and calculate their population allele frequencies using 1 server with 32 cores and 145 GB of memory. The Great Genotyper opens the door to new ways to study SVs. For example, using the premade index, we demonstrate the Great Genotyper's application in finding pathogenic variants by calculating accurate allele frequency for novel SVs. Also, we used it to create a 4k reference panel by genotyping variants from the Human Pangenome Reference Consortium (HPRC). The new reference panel allows for SV imputation from genotyping microarrays. Moreover, we genotype the human GWAS Catalog and merge its variants with the 4k reference panel. We show 6,253 events of high linkage between the HPRC’s SVs and nearby GWAS single-nucleotide polymorphisms, which can help in interpreting the effect of these SVs on gene functions. This analysis uncovers the detailed haplotype structure of the human fibrinogen locus and revives the pathogenic association of a 28-bp insertion in the *FGA* gene with thromboembolic disorders.

**Conclusion:**

The Great Genotyper solves the N+1 problem for population-scale genotyping of small and structural variants, offering both high accuracy and efficiency. Its ability to rapidly re-genotype large cohorts paves the road for several new studies of SVs.

## Introduction

Maya Angelou eloquently stated, “In diversity, there is beauty and there is strength.” This principle is particularly relevant to genomics studies, emphasizing the importance of exploring genetic diversity across large cohorts and populations. Such research is crucial for advancing our understanding of evolution [1, 2], genetic adaptations [3], and gene–disease associations [4, 5]. Genetic diversity originates from various mutations, including single-nucleotide variants (SNVs), small insertions and deletions (less than 50 base pairs), and structural variants (greater than 50 base pairs). Notably, structural variants (SVs) enhance genomic diversity 15 times more than SNVs [6] and significantly affect gene function [7]. However, SVs are understudied compared to smaller variants due to the limitations of short-read sequencing (SRS), which often yields high false-positive rates and inconsistent recall, varying from 10% to 70% [8]. In contrast, long-read sequencing (LRS) provides more reliable precision and recall rates [8] and is used in both mapping [9–11] and assembly-based approaches [12], the latter of which helps mitigate mapping biases to a linear genome reference. Despite its advantages, LRS remains prohibitively expensive for comprehensive population-scale analysis, and the volume of LRS data available still pales in comparison to that of SRS. As a result, there is a pressing need to develop computational techniques that utilize the precise variant discovery capabilities of LRS while maximizing the extensive data produced by SRS.

To effectively utilize the abundant short-read sequencing data available, while addressing the limitations of short-read SV callers, specialized genotypers analyze the presence and genotype of SVs, whether identified through variant calling from SRS or LRS, in SRS samples [13–17]. Tools such as Paragraph [14] and GraphTyper2 [16] realign reads to a variation-aware graph, minimizing mapping bias and determining genotypes from this realignment. PanGenie [17] uses *k*-mers specific to all potential alleles to genotype phased variants from pangenomes, minimizing mapping bias. Furthermore, PanGenie integrates genotyping and imputation, utilizing the phasing information from the pangenome to infer genotypes in regions lacking coverage, thereby achieving superior performance compared to other SV genotypers. Unlike these single-sample genotypers, muCNV utilizes population data to refine genotyping by modeling read mapping statistics across multiple samples, enhancing genotyping accuracy [15].

SV genotypers generally achieve higher recall and precision compared to direct variant calling in SRS samples. For instance, Huddleston et al. [18] used LRS to analyze SVs in 2 human genomes and found that 90% of these SVs were missing in the 1000 Genomes call set, yet 61% could still be genotyped using SRS. Recent population-scale studies have therefore adopted a combined approach of variant calling and genotyping: initially, variants are identified from a few LRS samples or numerous SRS samples, and then the identified SVs are merged and genotyped in a larger SRS cohort [19]. For instance, Kirsche et al. [20] used Paragraph [14] to genotype variants from 31 LRS samples in a cohort of 1.3k SRS samples from the 1000 Genomes Project (1kGP) [21]. Similarly, GraphTyper2 was employed to build graphs from SVs detected in 50k Icelandic SRS samples [16] or 2k dog SRS samples [22], which were then re-genotyped using the same SRS samples to improve recall. With the same concept in mind, the Human Pangenome Reference Consortium (HPRC) [23] applied PanGenie to genotype the pangenome variants in 3.2k SRS samples from 1kGP [21]. Similarly, Goo Jun et al. [24] used MuCNV to jointly genotype TopMed SVs in 139k SRS samples. These genotypers enable large-scale population genotyping of gene catalogs, pangenomes, and candidate disease-associating variants.

The current SV genotypers, while fast and scalable, face significant challenges at the population level. These genotypers require downloading and reprocessing all the raw SRS data to genotype even a single new variant, a demand that is increasingly impractical. This issue exemplifies a computational challenge known as the N+1 problem [25]. In today’s era of extensive sequencing, new lists of variants emerge daily, and a reliable estimation of their allele frequencies is important for interpretation. For instance, the number of pangenomes for humans [23, 26] as well as numerous other species [27–30] is increasing. Similarly, databases like dbVar [31], gnomeAD [32], TopMed [33], and ClinVar [34] are constantly expanding their variant collections. The N+1 challenge also affects disease gene discovery studies in probands [35]. LRS can produce phased, high-quality SVs, and identifying pathogenic variants involves filtering out common variants and focusing on rare ones. However, matching these variants in public databases poses challenges, and the reliability of allele frequencies in SV catalogs is dubious when calculated in small or distinct subpopulations or when using methods with low recall. Therefore, solving this computational bottleneck is crucial to optimize the usage of genomic data for advancing precision medicine and enhancing our understanding of genetic diversity.

A new trend in genomics [36, 37] involves preprocessing raw sequencing data to create searchable indexes that can be directly utilized by downstream applications. One state-of-the-art tool in this field is Metagraph, highly efficient software for indexing the *k*-mer content of massive sequencing datasets using a Counting Colored de Bruijn Graph (CCDG). A CCDG encodes *k*-mers along with an array that annotates their counts in each sample, preserving essential genotyping information in a compact format [38].

Building on this foundation, we introduce the Great Genotyper, an alignment-free genotyping tool designed for both structural and small variants. The Great Genotyper efficiently partitions raw sequencing data from thousands of samples, then indexes them into CCDGs using Metagraph. The CCDG is used to genotype any set of variants, eliminating the need for raw data and solving the N+1 problem. The Great Genotyper leverages PanGenie’s genotyping model and population-derived data to enhance the genotyping accuracy.

As a use case, we used the Great Genotyper to partition and index 183 TB of raw sequences from 4.2k human samples into an 867-GB partitioned CCDG. The index is used to genotype 26.8 million variants from the human pangenome. Additionally, we demonstrated how these population-level genotypes can serve as an imputation panel for structural variants and enable the annotation of structural variants based on their linkage to nearby genome-wide association study (GWAS) single-nucleotide polymorphisms (SNPs).

## Results

### The Great Genotyper: A workflow for genotyping small and structural variants in thousands of short-read samples

**Figure 1:**
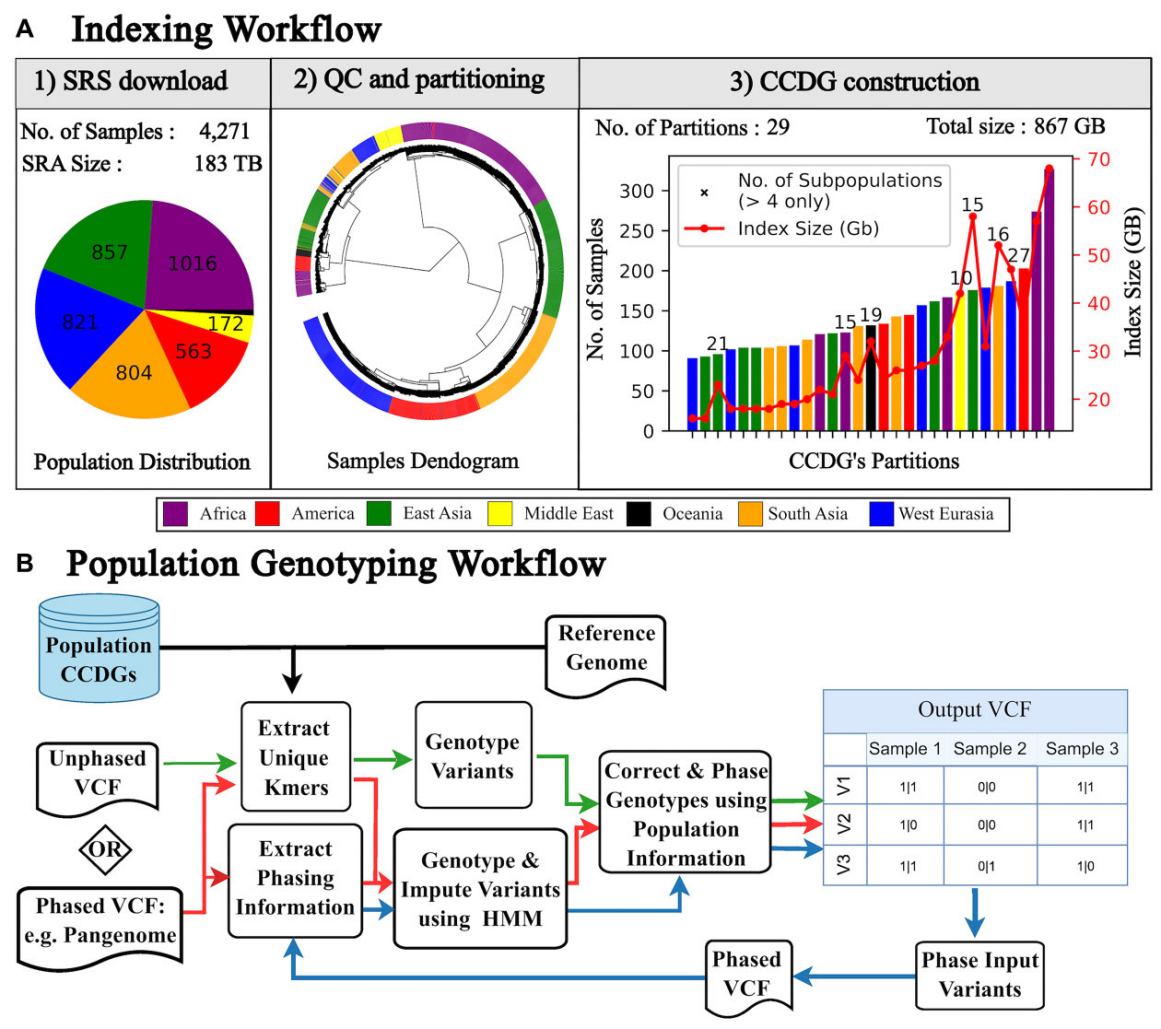
The Great Genotyper workflows. The indexing workflow (A) depicts the high-level pipeline for creating population CCDGs. The workflow downloads and computes the unitigs of each sample individually (A1). A sourmash signature is calculated for each sample to be used for alignment-free quality control and sample partitioning (A2). Lastly, a subgraph is created for each partition of samples (A3). The genotyping workflow (B) describes 3 population genotyping workflows illustrated with a different color of arrows: The HMM workflow (red) genotypes and imputes phased variants using a high-quality HMM model, the *k*-mer–based workflow (green) rapidly genotypes unphased variants, and the 2-pass workflow (blue) enhances the recall of the *k*-mer–based workflow by genotyping its output phased variants using the HMM workflow.

The Great Genotyper solves the problem of genotyping a new list of variants in a given population (i.e., the N+1 problem) by deploying 2 independent workflows. The first is an indexing workflow that performs all the heavy lifting once by creating a CCDG using raw SRS to represent the population (Fig. 1A). Once created, the CCDG can be reused by a population genotyping workflow (Fig. 1B) to genotype a pangenome, phased variants, or unphased variants in the cohort of SRS samples.

The indexing workflow preprocesses raw sequencing datasets to extract *k*-mer content, perform alignment-free quality control (QC), and partition samples. An individual CCDG is generated for each partition. The genotyping workflow utilizes each subindex independently to maximize parallelization. The outputs from genotyping 1 or more subindexes are eventually combined for final population-level QC and imputation.

The design of the Great Genotyper minimizes computational time while avoiding memory bottlenecks when genotyping thousands of samples, especially when distributed across high-performance computing (HPC) or cloud systems. Preprocessing of raw data can be executed independently for each sample, and the runtime is primarily influenced by sample size, averaging approximately 2 hours per sample on a node with 16 threads and 20 GB of memory (Supplementary Fig. S8). The time of subindex creation depends on the number of samples, as illustrated in Supplementary Fig. S9. For instance, creating an index for 150 samples with an average coverage of 30× requires approximately 35 hours on a node with 32 threads and 200 GB of memory. In contrast, genotyping a subindex depends primarily on the number of variants in a subindex of a recommended range of samples. However, genotyping 5 million variants in a subindex with 150 samples needs less than 2.5 hours on a server node with 120 GB of RAM and 32 threads (Supplementary Fig. S10). Since subindexes are independent, their generation and genotyping can be distributed across multiple nodes, enabling efficient scaling. This design also facilitates the addition of new samples by creating a new independent subindex. The final step, which involves aggregating all output Variant Call Format (VCF) files and imputing missing genotypes, depends primarily on Beagle software, which is known for its efficiency and scalability (Supplementary Fig. S11).

In this study, the Great Genotyper was used to construct a CCDG for 140 human populations (Fig. 1A and Supplementary Fig. S1). This involved downloading 4.2k high-coverage (30×) whole-genome sequencing (WGS) samples from the 1kGP [21], the Human Genome Diversity Project (HGDP) [39], and the Simons Genome Diversity Project (SGDP) [40]. Initial QC revealed 7 samples with unexpectedly low genome coverage and 4 discrepancies between reported and predicted sex (Supplementary Figs. S2, S3). Subsequently, a dendrogram of sample sketches was generated (Fig. 1A.2), identifying 29 partitions, each encompassing between 100 and 350 closely related samples (Fig. 1A.3).

Processing the 183 TB of raw data required approximately 21 days of preprocessing time using 16 nodes, each with 16 cores and 20 GB of RAM. The creation of 29 subindexes took an additional 10 days, using four 32-core servers with 200 GB of RAM each. The resulting subindex sizes ranged from 16 GB to 68 GB, with a total combined size of 867 GB.

Building upon the CCDGs created in the indexing workflow, the genotyping workflow (Fig. 1B) empowers the analysis of any variant list across all samples without requiring raw reads or mapping. It begins with 3 key inputs: a list of pregenerated CCDGs, a reference genome, and a variant list (phased or unphased). Depending on needs, 3 different workflows can be chosen: (i) *k*-mer–based workflow, which efficiently genotypes unphased variants; (ii) hidden Markov model (HMM) workflow, which handles both genotyping and imputation for phased variants; and (iii) 2-pass workflow, which genotypes and imputes unphased variants, leveraging population information to determine their phase and impute missing data.

Both the *k*-mer–based and HMM workflows start by extracting *k*-mers unique to the variant regions and querying their count data for all samples within the CCDGs (Fig. 1B). The *k*-mer–based workflow determines initial genotypes by comparing the counts of unique *k*-mers to the average sample coverage for each sample. This identifies variants present in each sample without relying on phasing information. In contrast, the HMM workflow tackles phased variants by genotyping and imputing them using the HMM implemented in PanGenie [17]. This enables the imputation of genotypes in regions with low coverage or complexity. Following initial genotyping, both workflows undergo a 2-step refinement. The first step is to filter low-quality genotypes after comparing the genotype qualities for each variant across all samples. The second step utilizes Beagle [41, 42] to statistically impute low-confidence genotypes and phase the resulting variants.

The third workflow is a pipeline to genotype and impute unphased variants. It starts by running the *k*-mer–based workflow to create a reference panel using the input variants and samples in the CCDGs. This reference panel is then used to phase the input variants. After that, the HMM workflow is employed on the phased variants to obtain more precise genotypes in the indexed population.

### Achieving population genotyping in a matter of hours with no decrease in accuracy

**Figure 2:**
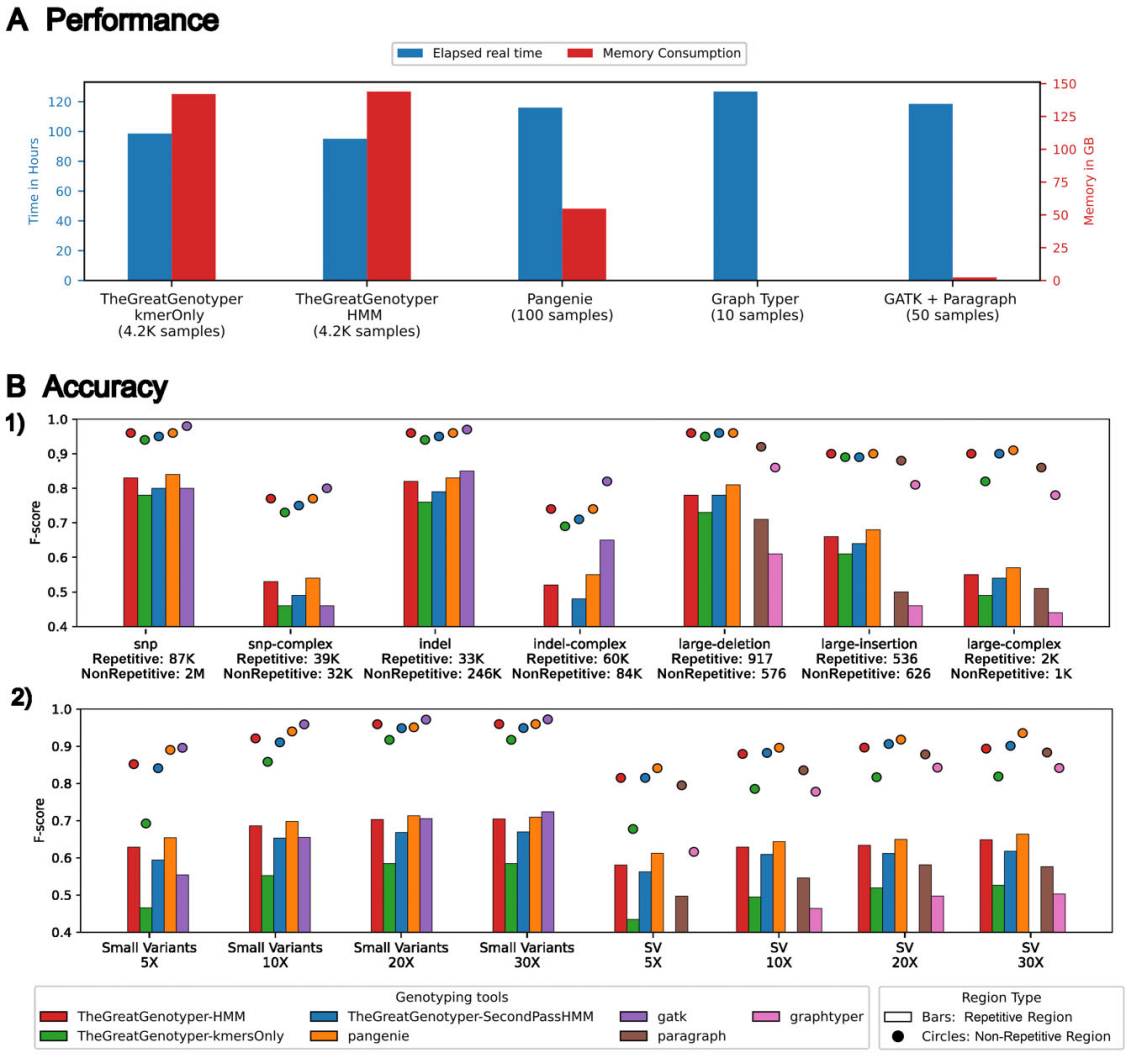
The Great Genotyper provides unparalleled performance compared to the state of the art, with no compromise on accuracy. (A) The running time and memory usage of different tools used to genotype 4.5 million phased variants (including structural variants and small variants). The Great Genotyper is currently genotyping 4,271 samples at 30× coverage, assuming the indexing of the samples was already built, while the other genotypers are handling 10 to 100 samples. (B) The *F*-scores of different genotyping methods for different classes of variants. B2 illustrates the effect of coverage on the *F*-scores of different genotyping methods for small and structural variants. In both B1 and B2, the variants are categorized based on the complexity of the genomic loci into variants located in repetitive (shown as bars) and nonrepetitive regions (shown as circles).

The performance of the Great Genotyper was evaluated using its *k*-mer–based workflow (for unphased variants) and HMM-based workflow (for phased variants). After indexing the 4.2k WGS samples, the Great Genotyper genotyped 4.5 million variants across all samples in approximately 100 hours, utilizing 32 cores and 145 GB of memory, as shown in Fig. 2A. For comparison, we ran PanGenie and GraphTyper2 on raw FASTQ data for a single sample. PanGenie required about 1 hour, while GraphTyper2 took roughly 12 hours on the same machine. Notably, PanGenie processes raw reads directly, whereas GraphTyper2 requires read alignment, which we performed using the BWA-MEM algorithm. Extrapolating these runtimes, both tools would require months to genotype all 4.2k samples for each new variant set. This comparison shows that, due to its one-time indexing cost, the Great Genotyper may not be faster than single-sample genotypers when analyzing a single variant set. However, it offers substantial speed advantages when genotyping large cohorts repeatedly across multiple variant sets (see Supplementary Table S4 for benchmarking of the Great Genotyper, PanGenie, and GraphTyper2 regarding core algorithms, applications, and runtime metrics).

For benchmarking of precision and recall of the Great Genotyper with other state-of-the-art genotypers, we genotyped SVs and small variants derived from the NA12878 haploid-resolved assemblies using the 30× SRS of HG00731 (see Methods and Supplementary Fig. S4 for the design of benchmarking and Fig. 2 for the detailed results).

The Great Genotyper’s HMM and PanGenie exhibit superior *F*-scores for phased SVs, achieving 0.91 in nonrepetitive regions. Paragraph and the *k*-mer–based workflow follow closely, with *F*-scores of 0.88 and 0.87 for unphased SVs. Intriguingly, the 2-pass workflow accurately predicts the phasing information, boosting the *F*-score back to 0.91. In contrast, GraphTyper2 trails with an *F*-score of 0.80. The challenges increase in repetitive regions, where variability in results is more pronounced. Here, PanGenie and the HMM workflow score 0.63 and 0.61, respectively, followed by Paragraph and the *k*-mer–based workflow at 0.55. However, the 2-pass workflow enhances the *k*-mer–based approach’s *F*-score to 0.6, while GraphTyper2 lags with an *F*-score of 0.48.

For small variants, GATK leads, achieving *F*-scores of 0.97 and 0.70 in nonrepetitive and repetitive regions, respectively. The Great Genotyper’s HMM and PanGenie are close behind, with *F*-scores of 0.95 in nonrepetitive areas. The *k*-mer–based workflow scores 0.93, improving slightly to 0.94 with the 2-pass workflow. In repetitive regions, PanGenie matches GATK’s 0.70 *F*-score, while the HMM workflow slightly trails at 0.69. The *k*-mer–based workflow struggles in these regions and scores 0.6 but is improved to 0.65 by the 2-pass workflow. Overall, the Great Genotyper consistently demonstrates competitive genotyping accuracy compared to PanGenie across most scenarios, and it represents the most accurate option for genotyping unphased SVs with the 2-pass workflow.

Sequencing depth impacts the genotyping accuracy, as depicted in Fig. 2B2. Notably, all genotypers exhibit reduced accuracy at sequencing depths of 10× and 5×. Genotypers that incorporate phasing information, such as the Great Genotyper’s HMM and 2-pass workflows, as well as PanGenie, show the smallest decrease in accuracy. For instance, the accuracy of SV genotyping by the Great Genotyper’s HMM and PanGenie at 5× coverage drops by 8% and 9% in nonrepetitive regions, as well as 7% and 5% in repetitive regions, respectively. The *k*-mer–based workflow experiences a decrease of 14% and 9%, which the 2-pass model returns to 7% and 5% in nonrepetitive and repetitive regions, respectively. Last, GraphTyper2’s accuracy diminishes by 12% and 22% in nonrepetitive and repetitive regions, respectively.

The reduction in sequencing depth from 30× to 5× similarly affects the accuracy of small variant genotyping in both nonrepetitive and repetitive regions. PanGenie exhibits the smallest accuracy decline, by 7% and 5%, followed by the Great Genotyper’s HMM with 11% and 7%, as well as GATK with 8% and 17%. The *k*-mer–only model suffers a significant drop of 22% and 11%, but this is mitigated by the 2-pass model to 10% and 7% in nonrepetitive and repetitive regions, respectively.

### Facilitating population studies for small and structural variants

#### The Great Genotyper can help to find pathogenic variants

**Figure 3:**
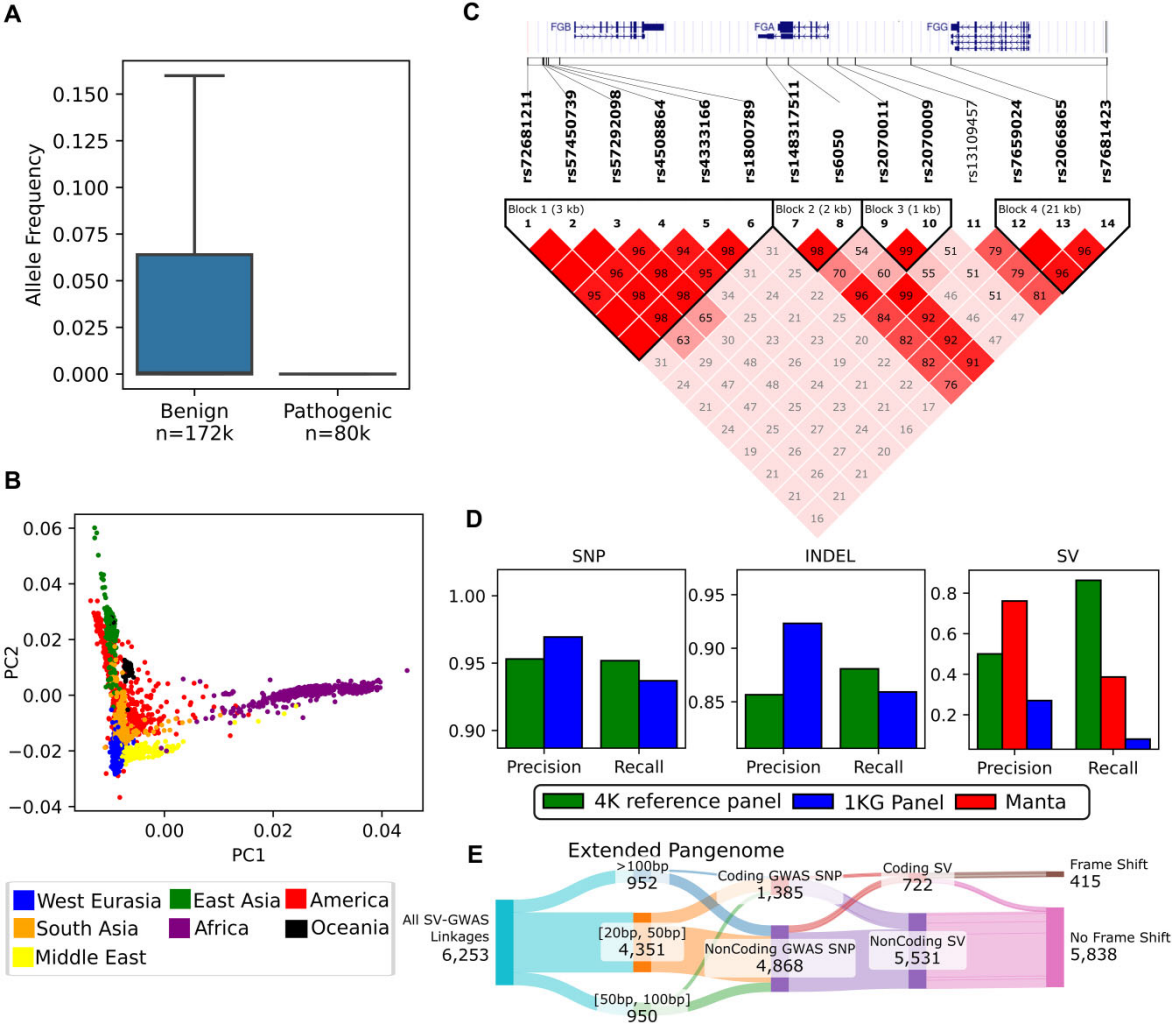
Applications of the Great Genotyper: We used the Great Genotyper to genotype all ClinVar and HPRC pangenome variants in 4.2k human samples. (A) A boxplot of the distinctive distributions of population allele frequencies for ClinVar variants when stratified by the pathogenicity of the variants (outliers are not displayed). (B) A plot of the first 2 principal components from a PCA for the genotypes of the HPRC pangenome variants; the 4.2k samples are colored by their ancestry. (C) An LD heatmap that highlights the associations of an insertion (dbSNP: rs148317511) and multiple GWAS SNPs, including rs6050-C; a peak associating SNP in a GWAS study of the circulating fibrinogen. (D) The precision and recall of variant imputation using the 4k reference panel in comparison to variant imputation using the 1000 Genomes panel and SV calling using Manta. (E) A Sankey plot summarizing 6,253 linkage associations between SVs from the HPRC pangenome and the GWAS catalog. The columns stratify linkages based on various traits of both SVs and GWAS SNPs: SV size, GWAS SNP impact on coding regions, SV impact on coding regions, and SV-induced frameshifts.

Filtering common variants is a widely used strategy in disease association studies. ClinVar, a public database, catalogs genomic variations in humans and their impact on health [34]. As a proof of concept, the *k*-mer–based workflow is applied to genotype the ClinVar database variants in the 4k samples of the CCDG index. Consistent with expectations, almost all pathogenic variants exhibit zero allele frequency in this healthy population, whereas benign variants display a broader range of frequencies (Fig. 3A). This demonstrates that calculating allele frequencies for a list of suspected variants in this indexed cohort is a reliable metric for prioritizing rare variants in studies of their pathogenic potential.

An additional experiment was conducted to highlight the impact of efficient re-genotyping at the population scale. The Great Genotyper was used to genotype 790 likely pathogenic variants identified in 14 recent human genetic studies across a cohort of 4,000 individuals. The Great Genotyper successfully confirmed the population allele frequencies of 749 variants with reliable Hardy–Weinberg equilibrium (HWE) significance threshold of $ p > 10^{-6}$, showing a strong linear correlation with their frequencies in the gnomAD database (Supplementary Table S3 and Supplementary Fig. S13).

#### Generation of 4k reference panel by genotyping HPRC variants in 4k samples

The current HPRC pangenome, consisting of 88 haplotypes, decomposes into a phased VCF containing 26.8 million variants, as previously described [23] (see Supplementary Table S1 for a detailed summary of variant types). The HMM workflow is used to genotype these variants in the prebuilt CCDG. The resulting output is a phased VCF of the HPRC variants in the indexed 4.2k samples, creating a new 4k reference panel. Principal component analysis (PCA) on the genetic variation within this 4k reference panel confirms the expected distribution of populations studied in the 1kGP, paving the way to generate cost-efficient similar panels for several other species (Fig. 3B). Subsequent sections will explore how this panel can facilitate various genomic applications.

#### Impute SV by using the 4k reference panel

Genotype imputation is a statistical method that predicts unobserved genotypes using reference sequences, thereby enhancing the density and scope of genetic analyses at reduced costs. This technique is especially valuable in increasing the power and consistency of genetic studies, including GWAS and fine-mapping efforts [43]. The 4k reference panel may replace the panel generated by the 1kGP project [44] while enabling the imputation of SVs. In this section, we demonstrate the precision and recall of imputing both small and structural variants using the 4k reference panel. Initially, pseudo-microarray variant calls are generated using the HG002 sample from the Genome in a Bottle (GIAB) project [45] by extracting variants at sites used in the Illumina Infinium OmniExpress-24, simulating microarray genotyping. The 4k reference panel is then employed to impute both small and structural variants. For benchmarking purposes, the 1kGP reference panel is used for imputing small variants and SVs. Also, SV calling from 30× SRS using Manta serves as another reference point. The output VCFs are compared against gold-standard GIAB datasets using hap.py (v0.3.12) [46] for small variants and truvari (v3.5.0) [47] for SVs (see the Data and Code Availability section). The 4k reference panel exhibits commendable precision and recall for the imputation of both types of variants, as depicted in Fig. 3D. When compared to the 1kGP reference panel, it displayed some reduced precision compensated by an increase in recall for SNPs and indel imputation. Conversely, the 4k reference panel shows remarkable recall of SV (86%), which surpasses the recall of not only the 1kGP reference panel but even SV calling from 30× SRS using Manta. The precision of the 4k reference panel is also higher than the 1kGP reference but still obviously lower than the precision of variant calling. These results highlight how the 4k reference panel can be leveraged to augment microarray genotypes with common SVs.

We further investigated the reasons behind the performance differences between the 1kGP reference panel and the 4k reference panel. Our analysis revealed that the number variant sites and their alternative haplotypes in the pangenome (used to create the 4k reference panel) are much higher than those in the 1kGP panel. Specifically, the pangenome covers 80k variant sites, while the 1kGP reference panel has only the 48k 1kGP reference panel. Moreover, 72% of the sites in the HPRC pangenome have multiple alternative alleles, with an average 10.6 alternative alleles per position, whereas only 22% of the sites in the 1kGP panel have multiple alternative alleles, with an average 1.2 alleles per position (see Supplementary Fig. S12). The more accurate representation of polymorphic regions in the 4k reference panel enabled the imputation tool to identify the correct haplotypes more effectively.

#### Fine mapping of GWAS SNPs using SVs from the 4k reference panel

The 4k reference panel provides detailed insights into the structure of common haplotypes composed of small and structural variants. In particular, it allows the exploration of linkage disequilibrium (LD) between SVs and neighboring variants known to be associated with phenotypic changes. We initiate our investigation by annotating the SVs in the 4k reference panel using AnnotSV (v3.3.6) [48]. This reveals that approximately 463k SVs affect gene structures. Proceeding further, we compute the pairwise LD for each of these variants with all the variants located within a 1-MB window surrounding them. Our analysis indicates that 91k SVs exhibit a strong association with a neighboring variant, having an *r*^2^ value greater than 0.8.

We utilize the identified associations to illuminate potential causal variants in GWAS. Among the 91k SVs, 6,253 are found in strong linkage with GWAS SNPs. We compiled a table that includes these SVs, their annotations, associated GWAS SNPs, and other relevant metadata (see the Data and Code Availability section). This table should be a valuable resource elucidating the phenotypic effects of common SVs and help pinpoint some causal variants of the traits examined in these GWAS. We are using a Sankey plot in Fig. 3E as a flow diagram between the different categories of these associations based on the size of the SV, the impact of either the GWAS SNP or SV on coding regions, and the ability of the SV to cause a frameshift. Notably, 722 of these SVs impact the coding regions of genes, with 415 causing frameshift mutations.

We explore a specific example from our list in Fig. 3C, focusing on the human fibrinogen locus on chromosome 4. This 50-kilobase region includes 3 fibrinogen genes: the central FGA gene encodes the alpha chain, flanked by FGB and FGG encoding the beta and gamma chains, respectively [49]. Our reference panel shows an insertion of 28 bp at chr4:154584089 (dbSNP: rs148317511; ClinVar: RCV000247066) in a high linkage (phased *r* = 0.98) with rs6050-C, a missense mutation in FGA associated with venous thromboembolism [50– 53] and chronic thromboembolic pulmonary hypertension [53, 54]. The insertion is reported in ClinVar as a benign variant. Surprisingly, further digging in the literature shows that the variant was once known as the Taq I polymorphism because it created an additional restriction site for Taq I [55]. The allele was found to enhance the stability of FGA mRNA in vitro [51]. This was explained by the ability of the insertion to oppose the suppressive effect of hsa-miR-759 on the 3′ UTR of FGA [54]. These findings suggest that the ClinVar information on the variant warrants re-evaluation.

Interestingly, our panel is able to capture the haplotype structure of the fibrinogen locus and shows how the 28-bp insertion fits in. For example, rs6050 is known to be in high linkage with rs7681423, which is upstream to FGG and shows a peak of association with $ \gamma ^\prime$ fibrinogen. Both SNPs are known to have no significant association with total fibrinogen levels and no linkage with rs1800789; SNP in FGB shows the strongest association with total fibrinogen level, but not with $ \gamma ^\prime$ fibrinogen [56]. The panel confirms these relationships between the 3 SNPs and shows that the insertion allele has some linkages (D′ = 0.76) to rs7681423 and no linkage to rs1800789. Also, the panel shows a unique haplotype (D′ = 0.96) of the insertion and rs2070011-A, an allele of FGA’s promoter causing higher expression of the gene. This haplotype is different from the haplotype of rs6050 and rs7681423.

## Discussion

The Great Genotyper serves as a practical solution for population genotyping at massive scales. It provides the ability to genotype a new set of variants, whether small or structural, in thousands of SRS samples in just a matter of hours. The Great Genotyper can do this by providing a novel solution for the chronic N+l problem by eliminating the need to download and process terabytes of raw sequencing data. Instead, the Great Genotyper operates using a prebuilt CCDG, effectively decoupling intensive data preprocessing from the actual genotyping process. Therefore, the Great Genotyper is best suited for re-genotyping scenarios, which allow for quick calculation of population allele frequencies for any new set of variants. Also, it enables instant genotyping of the same dataset samples for any new set of variants. With this design, genotyping a large population once for a given set of variants using the Great Genotyper is still computationally on par with leading state-of-the-art genotyping tools. However, thanks to its prebuilt index, re-genotyping the same cohort for any new set of variants becomes a much easier computational task (Supplementary Table S4).

To add another dimension to the scalability of the Great Genotyper, it adopts a distributed design that allows its CCDG to be composed of multiple subindexes. Therefore, new samples can be appended easily as an additional subindex without the need to recompute the entire structure. In terms of input, the Great Genotyper is versatile; it accepts any set of phased or unphased variants, along with the reference genome. The outcome is the phased genotypes of all input variants in the indexed samples. In this article, 183 TB of SRA files for 4.2k human SRS samples are indexed to generate an 867-GB CCDG to enable unprecedented efficiency in calculating allele frequencies of any list variants in the human population. As a proof of concept, the index is used to genotype the HPRC pangenome variants as an example of phased variants, genotyping all unphased ClinVar variants and candidate variants in 14 studies of human genetic diseases.

The Great Genotyper does not sacrifice quality for scalability. On the contrary, the scalability empowers the Great Genotyper to jointly genotype thousands of samples, which, in turn, enhances the genotyping quality even more. The *k*-mer–based genotypers such as Nebula and PanGenie have previously demonstrated the potential of *k*-mers for precise genotyping. They leverage the specificity of variant-specific *k*-mers, using shifts in the counts of these *k*-mers as indicators to genotype the variants. The Great Genotyper reinforces this approach, considering the counts of these *k*-mers across an entire population of samples. This innovation facilitates the calculation of a confidence measure for each genotype based on the collective population data. Furthermore, the tool is equipped to impute missed genotypes through a 2-tiered approach. Initially, imputation is rooted in the phasing information of the variants, either provided as input or derived from the large cohort genotypes. Subsequently, the Great Genotyper integrates Beagle, leveraging the high-confidence genotypes within the population to further impute genotypes. This dual-phase imputation process ensures that the Great Genotyper can deliver performance on par with PanGenie, even if some data are compromised during the *k*-mer count preprocessing while indexing to enable better data compression, as described in Supplementary Fig. S7.

The enhanced accuracy and scalability of the Great Genotyper pave the way for valuable downstream applications in genomics. For instance, accurate allele frequencies can now be directly derived from sequences rather than merging information from sparse studies or variation databases that rely on variant calling in SRS studies. Such accurate determination of allele frequencies can play a pivotal role in pinpointing causal variants in disease–gene discovery studies (Supplementary Table S3). This is even more important for species that lack human-level variance annotation. Furthermore, simultaneous genotyping and phasing of common variants enables dramatically improved resolution for understanding the haplotype structure within and across populations. As an example, genotyping the HPRC pangenome variants in 4.2k samples produces what we call “the 4k reference panel (4kRP).” We show how the 4kRP can be used to impute common SVs with a recall rate that surpasses some short-read callers like Manta.

Taking our analysis further, we explore the 4kRP for SVs in high LD with known GWAS SNPs. We limit our focus to 91k SV variants impacting gene structures. Intriguingly, we discover that approximately half of these SVs exhibit strong associations with at least 1 neighboring variant, including 6,253 GWAS SNPs. We are optimistic that our findings will contribute to a deeper comprehension of the relationship between genotype and phenotype concerning these structural variants.

Although the Great Genotyper is effective in generating high-quality genotypes for both small and structural variants, it does have certain limitations. First, some variants cannot produce specific *k*-mers because the *k*-mers from the alternate sequences may also be present in other parts of the genome. Such variants cannot be genotyped precisely by *k*-mer–based approaches. This limitation, however, is partially offset through imputation. Furthermore, genotyping copy number variants is beyond the capabilities of the current version of the Great Genotyper. While it is not an insurmountable challenge, it requires development of a dedicated genotyping model. Another constraint is that the Great Genotyper utilizes 2 separate imputation models, as they are implemented in 2 distinct tools, PanGenie and Beagle. A unified model tailored specifically for imputing genotypes using the *k*-mers in the CCDG could both enhance the accuracy and boost the performance.

The Great Genotyper opens many doors for future genomic applications. Creating more CCDGs to represent specific subpopulations or individuals exhibiting specific traits, like autism, is crucial for understanding the role of genomics in these cohorts. Moreover, while most population studies have been conducted on humans [57], this approach is applicable to many other organisms. The Sequence Read Archive (SRA) [58] is a vast reservoir of short-read samples for nonhuman organisms. Generating CCDGs for these samples will facilitate population-scale studies for other species.

The current CCDG for the human population and the additional CCDGs to be created for other cohorts are invaluable resources with potential applications that extend beyond genotyping. For instance, variants can be directly called from the graph using methods such as Corticall [59]. Additionally, they can aid in subsetting pangenomes by selecting segments of the pangenome that have *k*-mers present in a specific population, thereby creating a more streamlined pangenome tailored to that population. We encourage the community to explore and uncover more ways to harness the extensive genomic diversity revealed by the CCDG.

## Conclusion

The Great Genotyper can transform population genotyping into a routine task using a flexible CCDG representation of populations. Its scalability allows the improvement of genotyping quality by using population information. The tool’s practicality aids in expanding variant lists into broader dimensions, revealing complex genomic details. We demonstrate its potential in applications such as creating SV imputation panels, finding SV associations with variants from databases like the GWAS catalog, and accurately calculating population allele frequencies. The CCDG, comprising 4.2k human samples, contains a vast genomic variation spectrum, accessible through the Great Genotyper or other methods, leading to enhanced genomic insights. Producing more CCDGs for additional cohorts or species will further optimize the use of existing SRS samples.

## Methods

### Design overview and foundational software

The Great Genotyper is built on the foundation of multiple powerful tools, including Metagraph [38], for creating compressed, searchable indexes (CCDG); PanGenie [17], for its advanced haplotype-aware genotyping capabilities; Beagle [41, 42], recognized as one of the best imputation tools; and Sourmash [60] and Snipe [61], which provide a fast and efficient method for quality checking and partitioning. In this section, we will highlight the key features of these tools.

#### MetaGraph

MetaGraph [38] is a software designed for indexing billions of *k*-mers using compressed data structures. At its core is the CCDG, which extends the traditional de Bruijn graph structure. Nodes represent unique *k*-mers, and edges capture *k −* 1 overlaps between them, with each node annotated by an array containing *k*-mer counts across individual datasets. This design supports efficient sequence-based queries or graph traversal, returning both the datasets containing the sequence and the associated *k*-mer counts.

#### PanGenie

PanGenie [17] genotypes phased small and structural variants in SRS samples using a *k*-mer–based approach. The method begins by extracting a set of *k*-mers unique to the variants targeted for genotyping, ensuring specificity in variant representation. Genotyping is performed using an HMM inspired by the Li-Stephens haplotype copying model [62]. This model calculates emission probabilities by comparing the observed *k*-mer counts in the sample to the expected counts for each variant haplotype. The likelihood of a genotype is proportional to the match between these counts, with penalties applied for mismatches or missing *k*-mers. If insufficient *k*-mer support exists for a specific variant, the model imputes the genotype by propagating information from nearby variants on the same haplotype. This integration of local context helps address uncertainties in the data, ensuring more accurate genotype calls even in regions of low coverage or complex variation. The genotyping model assigns a confidence measure to each output genotype by calculating the likelihoods of all possible genotypes and selecting the one with the highest probability. Confidence is determined by the difference between the highest probability and the next highest, with a larger difference indicating greater confidence.

#### Beagle

Beagle [41, 42] is a tool for phasing population genotypes without requiring a reference panel, provided the population size is sufficiently large. Additionally, Beagle imputes missing genotypes statistically by modeling linkage disequilibrium using the Li-Stephens HMM, thereby generating a reference panel suitable for imputing and phasing other samples. Beagle competes favorably with state-of-the-art solutions in terms of processing time and accuracy, efficiently handling datasets with hundreds of thousands of samples. Its capabilities have enabled the construction of reference panels for large-scale projects such as UK Biobank and TOPMed [42].

#### Sourmash, Snipe, and kSpider

Sourmash [60] is a tool designed for comparing genomes and sequencing datasets, with a particular emphasis on metagenomics. It creates compact FracMinHash sketches, which are probabilistic representations of the *k*-mer content in each dataset. Unlike traditional approaches that select a fixed number of hash values, FracMinHash uses a fraction-based sampling method, selecting hash values based on a predefined proportion of the hash function’s output. This approach allows for more precise and efficient comparisons of genomic datasets. Snipe [61] is a quality control tool that uses an alignment-free approach to compare the Sourmash sketches of next-generation sequencing datasets against a target reference, producing comprehensive quality metrics, including accurate calculations of sequence depth and coverage, contamination, sex determination, and genetic variance. kSpider [63] is a tool that supports lightweight clustering of thousands of Sourmash sketches, enabling efficient partitioning based on their sequence content, before MetaGraph indexing.

#### The Great Genotyper design

The Great Genotyper is designed to address the unique challenges of population genotyping, which differ significantly from single-sample approaches. Processing thousands of samples introduces scalability challenges, particularly in balancing memory requirements and runtime, but also offers opportunities to leverage population-level information. To solve these challenges, the Great Genotyper employs 2 independent workflows. The indexing workflow performs the heavy computational tasks upfront, creating a CCDG from raw SRS to represent the population. This CCDG can then be reused by the population genotyping workflow to efficiently genotype any new list of variants.

The indexing workflow starts by preprocessing of raw SRS to quantify, error-trim, and summarize their *k*-mer content by kmc [64] and Metagraph [38]. In the next step, a lightweight pipeline is deployed for alignment-free QC and partitioning of input samples using Snipe [61] and kSpider [63]. Please see "Short-read sample preprocessing and partitioning" in Methods for more details. Finally, each partition is indexed by Metagraph to generate a CCDG. The Great Genotyper tailors the indexing workflow of Metagraph to minimize the index size without sacrificing the genotyping accuracy. For example, logarithmic scaling of *k*-mer counts and graph simplification steps, which can reduce the size of the index, were avoided to prevent accuracy degradation. Further details on determining the best indexing parameters are discussed in Methods. Instead, memory requirements were addressed by splitting the index into multiple subindexes that could be processed independently in a map-reduce fashion, with results combined to make population-level decisions.

For the genotyping workflow, the Great Genotyper builds on the C++ codebase of PanGenie (a single-sample genotyper) [17] and implements new logic to tackle population-level challenges and opportunities. PanGenie’s original design parallelizes computation by chromosome and stores intermediate data in memory. In contrast, the Great Genotyper is designed to parallelize around samples instead of chromosomes, with the ability to write intermediate data to disk. This enables scaling across distributed systems and allows for controlled memory usage. In addition, PanGenie calculates *k*-mer counts and estimates genome coverage from these counts for a single input sample before genotyping. On the other hand, these sample-level statistics are calculated during the indexing step in the Great Genotyper to be reutilized with any new genotyping task. Moreover, the Great Genotyper has a novel module that makes use of the population-level information to filter out low-quality genotypes and utilizes Beagle’s state-of-the-art imputation and phasing algorithms to increase the recall in the final output.

Lastly, we developed a Snakemake workflow to integrate all the modules described above into the indexing and genotyping processes, ensuring ease of use (see Data and Code Availability).

### Short-read sample preprocessing and partitioning

Upon the download of each sample, kmc [64] is used for *k*-mer counting with a minimum count of 3 to filter out singletons and doubletons, which are likely sequencing errors. In addition, Metagraph [38] is utilized to identify the unitigs and retain only the average *k*-mer count per unitig, thus smoothing *k*-mer counts. This smoothing reduces the size of the *k*-mer counts to about one-tenth while maintaining high genotyping accuracy (see below). Subsequently, alignment-free quality control is done using Sourmash [60] and Snipe [61]. This process begins by downsampling raw sequences into representative summary sketches (i.e., FracMinHash sketches calculated using Sourmash). A Sourmash sketch is created for each sample using a *k* size of 51 and a subsampling scale of 10k, which entails keeping a single hash for every 10,000 *k*-mers. A similar sketch at the same scale is created for the GRCh38 reference genome. Snipe intersects both signatures to generate approximate estimates of the genome coverage and sequencing depth as well as sex confirmation (Supplementary Figs. S2 and S3). Subsequently, kSpider [63] calculates pairwise similarities between all samples based on their Sourmash sketches. To alleviate skew from the sex chromosomes, the sequence hashes of chrY are subtracted from all sketches. Hierarchical clustering is employed using the Scipy library [65] to construct a dendrogram that can visualized (see Supplementary Fig. S6) by iTOL [66]. From the dendrogram, clusters are extracted into separate partitions of closely related samples to minimize the genetic diversity per partition and hence the final index size enhancing scalability for large datasets.

### Determining the best indexing parameters

We investigated the influence of sample preprocessing on genotyping accuracy to determine the best parameters for optimal results. Multiple CCDGs were generated from subsamples of the HG00731 SRS at sequencing depths of 5×, 10×, 20×, and 30×. Each CCDG was constructed using a different set of parameters, which are summarized in Supplementary Table S2, along with the final sizes of the CCDGs. Benchmarking was done as described in Supplementary Fig. S4 and later in the Methods.

Results in Supplementary Fig. S7 and Table S2 indicate that preprocessing methods do not impact samples with coverage exceeding 20×. For coverages of 10× and 5×, logging the counts is the most influential, significantly decreasing both the *F*-score and the final CCDG size. On the other hand, smoothing leads to a nominal drop in the *F*-score but notably reduces the CCDG size. Cleaning had a moderate impact on the *F*-score and caused a slight reduction in the CCDG size. These findings are instrumental in guiding our final decision to use smoothing of *k*-mer counts as the only preprocessing for input samples.

### Genotyping workflow

The Great Genotyper implements 2 genotyping workflows: one for genotyping and imputing phased variants using *k*-mer counts and phasing information, similar to PanGenie, and a novel workflow for unphased variants using *k*-mer counts only. For the phased variants, the Great Genotyper employs the PanGenie HMM model, which is based on the Li-Stephens model, as explained earlier [62]. For the unphased variants, we rely solely on emission probabilities calculated by the model to determine the most probable genotype for each variant. Emission probabilities for the possible alleles are calculated for each sample in the index in parallel. To manage memory efficiently, the emission probabilities for 1 subindex are written to disk before processing the next subindex. Moreover, this step can be scaled up by loading each subindex on a different node in a distributed system to minimize the running time.

Unlike single-sample genotypers, the Great Genotyper leverages the power of having a large population in the CCDGs to filter low-quality genotypes. This is possible because the genotyping model yields a confidence measure for the output genotypes. The components driving these confidence measures can primarily be distilled into 2 factors: the number of unique *k*-mers discovered for each variant haplotype and the count of these *k*-mers in the sample. The first factor is a constant across all samples since it is determined only from the reference genome and the variant to be genotyped. However, the second factor varies per sample. Some samples may present robust evidence for a particular genotype, while others may not due to either low coverage of the region in the sample or the exhibition of a different haplotype not present in the input haplotypes. Therefore, the Great Genotyper introduces a new quality metric by calculating the median of genotype confidences for each genotype. Thereafter, the genotypes falling below this median are discarded. This approach allows the Great Genotyper to establish a variable threshold calculated using the results from all the samples, providing a balanced way to sift through the variants. For variants abundant in unique *k*-mers, this threshold will be high, while more challenging variants will have a lower threshold, accommodating the varying levels of confidence in different scenarios. The final output of this step is a reference panel comprising the high-confidence genotypes. Moreover, the Great Genotyper running on a distributed computational system has the option to write these confidence probabilities in intermediate files to the disk of each node handling a batch of samples. An aggregation function uses these files to run the population-level genotype filtering step.

Finally, Beagle [41, 42] is employed to statistically impute the filtered, low-confidence genotypes using this reference panel, simultaneously phasing the resultant variants, thereby yielding phased genotypes for all samples. It is crucial to note that Beagle employs a different HMM model, albeit very similar to the one used in the HMM workflow. In Beagle, linkage disequilibrium is computed statistically from the high-confidence genotypes within the created reference panel. In contrast, the model in the HMM workflow utilizes the phasing information provided by the user in the input variants. The synergy between these 2 imputation methods not only enhances the results of genotyping but also broadens the application scope for the higher-quality HMM model, enabling its usage when phasing information is absent in the input VCF, as described in the 2-pass workflow in Fig. 1B.

### Benchmark experiment design

This section outlines the experimental design for benchmarking experiments conducted to compare the accuracy of the Great Genotyper with state-of-the-art genotyping tools: PanGenie v3.0.1, GraphTyper v2.72, Paragraph v2.3, and GATK v4.1.3, as described previously [17]. The experiment is structured into 2 components. The first involves creating benchmarking datasets, including a query variant set and a truth variant set.

To prepare these datasets, variant calling for HG00731 and NA12878 was performed by aligning their haplotype-resolved assemblies against the GRCh38 reference genome using Minimap2 v2.22 [67], followed by variant calling with PAV tools v2.2.6 [68]. To ensure robust benchmarking, only variants within high-confidence regions were selected. These regions correspond to areas where only 1 segment of the assemblies maps, excluding segmental duplications and highly repetitive regions, such as centromeres, which are beyond the scope of the evaluated genotypers.

The variants of NA12878 were used to represent the query variant set, while the variants shared between both samples represented the truth variant set. To achieve this, the VCFs of both samples were merged using bcftools v1.16 [69]. Variants unique to HG00731 were filtered out, and the merged VCF was split into 2 files: the test VCF, where the NA12878 sample column was retained, and the truth VCF, where the HG00731 sample column was retained. Both files contained the same set of variants, differing only in the sample column.

The second component involves running the genotypers and benchmarking their performance. Various genotypers were executed on the test VCF (NA12878) and the SRS derived from the HG00731 sample at different coverages (5×, 10×, 20×, 30×). The genotyping results were compared against the truth VCF using RTG v3.12.1 vcfeval [70] without the –squash-ploidy option. This configuration evaluates each local haplotype separately, enforcing strict genotype comparisons and penalizing mismatched zygosity. A variant was counted as a false positive (FP) if it was called 1/1 while the truth set had it as 0/1. Conversely, it was counted as a false negative (FN) if it was called 0/1 but was 1/1 in the truth set. A variant was counted as a true positive (TP) only if genotypes matched in both VCFs. Precision, recall, and F1-score were calculated based on TP, FP, and FN counts as follows:


\begin{eqnarray*}
\text{Precision} = \frac{\text{TPs}}{\text{TPs} + \text{FPs}}.
\end{eqnarray*}

\begin{eqnarray*}
\text{Recall} = \frac{\text{TPs}}{\text{TPs} + \text{FNs}}.
\end{eqnarray*}

\begin{eqnarray*}
\text{F1-score} = 2 \times \frac{\text{Precision} \times \text{Recall}}{\text{Precision} + \text{Recall}}.
\end{eqnarray*}

The benchmarking results were stratified based on whether the variant was located in a repeat region. Additionally, results were classified by variant type and size: SNPs, small insertions/deletions (<50 bp), large insertions/deletions ($\ge$ 50 bp), and complex insertions/deletions. Complex variants were defined as those that generate more than 1 breakpoint.

## Availability of Supporting Source Code and Requirements

- Project name: The Great Genotyper
- Project homepage: https://github.com/dib-lab/TheGreatGenotyper
- Benchmarking code: https://github.com/dib-lab/TheGreatGenotyper_benchmark
- Operating system(s): Platform independent
- Programming language: C++, Python, C, Fortran
- Other requirements: none
- License: GPL 3.0
- RRID: SCR_025487
- bio.tools ID: TheGreatGenotyper

The workflow for building CCDGs using Metagraph is available at https://github.com/dib-lab/TheGreatGenotyper/tree/master/DatabaseBuilder. Additional workflows for a variety of use cases can be found at https://github.com/dib-lab/TheGreatGenotyper_usecases. In addition, a general-purpose workflow for genotyping of the HPRC pangenome [71] is available at https://github.com/dib-lab/TheGreatGenotyper/tree/master/pangenome_genotyping.

## Additional Files

**Supplementary Fig. S1**. Global distribution of samples. The map demonstrates the representational breadth of selected samples across global populations. Small circles denote samples from the SGDP and HGDP datasets, while the larger circles represent those from the 1kGP, providing an overview of the coverage of world populations by these samples.

**Supplementary Fig. S2**. Alignment-free estimation of the human genome coverage in the 1kGP samples.

**Supplementary Fig. S3**. Gender discrepancies in 4 samples of the 1kGP metadata. The figure shows an agreement between alignment-free (right) and alignment-based (left) gender detection while disagreeing with the online metadata. On the right, the alignment-free approach identifies the gender by calculating the containment between the sample’s signature and the signature of the reference chromosome Y. These 4 samples showed unexpected deviation from the mean of its gender containment ratio (0.65 with SD = 0.039 and 0.08 with SD = 0.003 for males and females, respectively). On the left, the average sequencing coverage of chrY was calculated from the cram files. The color of the bars represents the sex as provided in the metadata, with blue bars denoting male and pink bars denoting female. Ideally, blue bars should be larger than pink bars; however, inconsistencies arise due to errors in the metadata.

**Supplementary Fig. S4**. Benchmark genotyping accuracy workflow. The figure represents the workflow for the benchmarking experiment. The gold color represents sample HG00731, and the blue represents the NA12878 sample.

**Supplementary Fig. S5**. Coverage effect on genotyping *F*-score. The figure illustrates the effect of coverage on the *F*-scores of different genotyping methods, differentiating between small variants (under 50 bp) and SV (above 50 bp). *F*-scores for variants located in repeated regions are shown as bars, while those in nonrepeated regions are shown as circles. Additionally, variants are categorized based on the complexity of their genomic location.

**Supplementary Fig. S6**. Addressing chrY bias in clustering. The figure depicts 2 attempts to create a dendrogram for the 4,271 samples. The outer circles consist of fine lines, each representing a sample, with the color of the lines signifying the population of the sample as per the metadata. The dendrogram is displayed within these circles, delineating the clusters. Excluding chrY hashes yields more homogeneous clusters, as illustrated in the left dendrogram.

**Supplementary Fig. S7**. *F*-score comparison between the different parameters of indexing. Metagraph’s preprocessing techniques influence the genotyping *F*-score. These techniques include Smoothing Counts, where only the average *k*-mer count per unitig for each sample (smooth10000000) is retained instead of preserving the individual *k*-mer counts (smooth1); Log Counts, which involves saving the log of *k*-mer counts to conserve space; and Clean, the error cleaning algorithm in the Metagraph documentation (https://metagraph.ethz.ch/static/docs/quick_start.html#graph-cleaning).

**Supplementary Fig. S8**. Computational performance of preprocessing steps. This figure illustrates the runtime and memory usage for preprocessing 1,000 samples. The preprocessing workflow involves 3 key steps: *k*-mer counting using KMC, constructing a compact de Bruijn graph with Metagraph, and graph traversal to generate unitigs. The x-axis represents the size of the dataset in CRAM format, and the y-axis shows the total wall-clock time (using 16 threads). Note that the tools allow for a configurable memory cap, which in this analysis is set to 20 GB. Selecting different parameters may impact both I/O performance and overall results.

**Supplementary Fig. S9**. Computational performance of creating a CCDG. This figure illustrates the runtime required to create a CCDG using Metagraph, assuming the preprocessing steps have already been completed. All samples are 30× coverage on average. The experiment was conducted on a server with 32 threads and 200 GB of memory. The primary factor influencing computational cost is the number of samples to be indexed in the CCDG. It is important to note that the workflow is highly parallelizable, and the wall-clock time can be significantly reduced if a high-performance computing (HPC) environment is available, allowing the use of multiple nodes.

**Supplementary Fig. S10**. Computational performance of initial genotyping per CCDG. This figure depicts the wall-clock time and memory usage required to genotype increasing number of variants in multiple subindexes with a different number of samples per index. The number of variants has a major impact on both time and memory demands compared to the number of samples per index. The experiment was conducted on a server with 32 threads.

**Supplementary Fig. S11**. Computational performance of population-level filtering and imputation. This figure depicts the wall-clock time and memory usage required to aggregate, filter, phase, and impute an increasing number of variants in multiple populations of samples. The time increases in a linear fashion with the number of samples but exponentially with the number of variants. On the other hand, the memory consumption does not show such a constant pattern because it depends on the Beagle’s partitioning of the chunk size that can be tweaked by the user. However, it was always less than 100 GB in all our experiments. All experiments were conducted on a server with 32 threads.

**Supplementary Fig. S12**. Comparison of structural variant allele counts in the 1kGP and 4K reference panels. This figure compares the distribution of structural variant allele counts per site in 2 datasets: the 1kGP panel (blue bars) and the 4K reference panel (green bars). The x-axis represents the number of structural variant alleles per site, while the y-axis displays the count of sites on a logarithmic scale. The 1kGP panel shows a narrower distribution with fewer multiallelic sites, as most sites contain 1 or 2 alleles, resulting in a total of 48,497 alleles across all sites. In contrast, the 4K reference panel exhibits a broader distribution, with a significant number of sites containing higher allele counts, contributing to a total of 80,275 alleles. This highlights the increased structural variant diversity captured in the 4K reference panel.

**Supplementary Fig. S13**. The Great Genotyper (GG) calculates the population allele frequencies of likely pathogenic variants identified in human genetic studies. Variants were selected from 14 rare genetic disease studies. A scatterplot shows a linear correlation between their allele frequencies in the gnomAD database (x-axis) versus the GG frequencies in 4,271 short-read whole-genome samples (y-axis) on a log–log scale.

**Supplementary Table S1**. Variant counts in the HPRC pangenome. The table details the number of variants from the decomposed VCF of the HPRC pangenome stratified by their type.

**Supplementary Table S2**. Impact of metagraph preprocessing on CCDG size. The table demonstrates how preprocessing methods like Smoothing Counts, Log Counts, and the Clean algorithm alter the size of the final CCDG. Smoothing Counts simplifies *k*-mer data to averages per unitig, Log Counts compresses the *k*-mer information logarithmic ally, and Clean removes errors.

**Supplementary Table S3**. Comparison of allele frequencies of likely pathogenic variants in human genetic studies in the gnomAD database and the Great Genotyper (GG).

**Supplementary Table S4**. Benchmarking Great Genotyper, PanGenie, and GraphTyper2 regarding core algorithms, applications, and runtime metrics. A single-sample genotyper like PanGenie is likely a better choice for genotyping a new sample for a defined list of variants. On the other hand, the Great Genotyper is designed for population-scale re-genotyping, which is best used for a quick calculation of population allele frequency of novel variants or genotyping a given population of samples many times for any new set of variants.

giaf112_Supplemental_Files

giaf112_Authors_Response_To_Reviewer_Comments_original_submission

giaf112_Authors_Response_To_Reviewer_Comments_Revision_1

giaf112_Authors_Response_To_Reviewer_Comments_Revision_2

giaf112_Authors_Response_To_Reviewer_Comments_Revision_3

giaf112_GIGA-D-24-00266_Original_Submission

giaf112_GIGA-D-24-00266_Revision_1

giaf112_GIGA-D-24-00266_Revision_2

giaf112_GIGA-D-24-00266_Revision_3

giaf112_GIGA-D-24-00266_Revision_4

giaf112_Reviewer_1_Report_Original_Submission Paul Horton -- 7/29/2024

giaf112_Reviewer_2_Report_Original_Submission Jana Ebler -- 8/6/2024

giaf112_Reviewer_2_Report_Revision_1 Jana Ebler -- 1/15/2025

giaf112_Reviewer_2_Report_Revision_2 Jana Ebler -- 5/7/2025

## Data Availability

All data are available through the GigaDB repository [72] and the UC Davis server, as follows: The GG indexes used in this study are at https://farm.cse.ucdavis.edu/~tahmed/GG_index/. The genotyped pangenome is at https://farm.cse.ucdavis.edu/~mshokrof/4k_reference_panel/. VCF output files from Truvari’s pairwise analysis of Chr22 SVs generated by imputation using the 4k reference panel or the 1000 Genome panel, or by SV calling using Manta, are at https://farm.cse.ucdavis.edu/~tahmed/truvari.tar.gz. List of the likely functional SVs in LD with GWAS SNPs can be found at https://farm.cse.ucdavis.edu/~mshokrof/GWAS_associations/. Lastly, the ClinVar genotyped data can be accessed at https://farm.cse.ucdavis.edu/~mshokrof/The_great_genotyper_clinvar/.

## Abbreviations

1kGP: 1000 Genomes Project; 4kRP: 4k reference panel; CCDG: Counting Colored de Bruijn Graph; GIAB: Genome in a Bottle; GWAS: genome-wide association studies; HGDP: Human Genome Diversity Project; HMM: hidden Markov model; HPC: high-performance computing; HPRC: Human Pangenome Reference Consortium; HWE: Hardy–Weinberg equilibrium; LD: linkage disequilibrium; LRS: long-read sequencing; PCA: principal component analysis; QC: quality control; SGDP: Simons Genome Diversity Project; SNP: single-nucleotide polymorphism; SNV: single-nucleotide variant; SRA: Sequence Read Archive; SRS: short-read sequencing; SV: structural variant; WGS: whole-genome sequencing.
