# Supplementary material for: The Great Genotyper: a graph-based method for population genotyping of small and structural variants
Source: Gigascience. 2025 Oct 3;14:giaf112. doi: 10.1093/gigascience/giaf112 (PMC12491952; doi:10.1093/gigascience/giaf112)
Supplement: giaf112_Supplemental_Files [file giaf112_supplemental_files.zip › Supplementary.docx]

**Supplementary Materials**


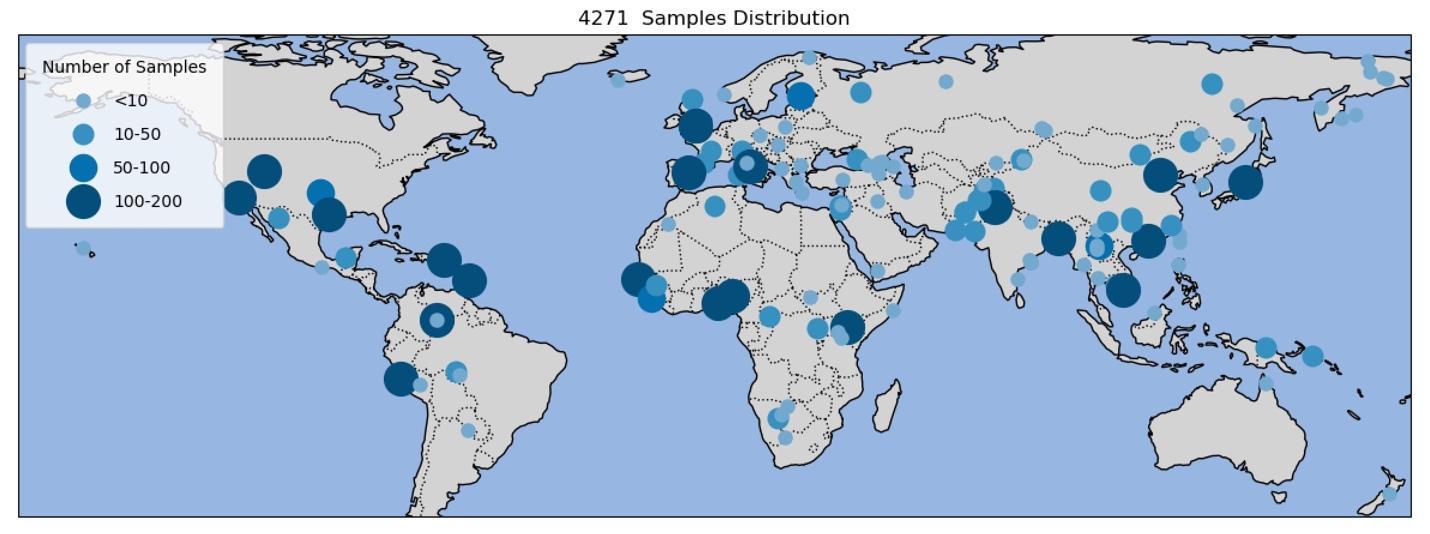


**Supplementary Figure 1:** **Global Distribution of Samples**: The map demonstrates the representational breadth of selected samples across global populations. Small circles denote samples from the SGDP and HGDP datasets, while the larger circles represent those from the 1KG project, providing an overview of the coverage of world populations by these samples.


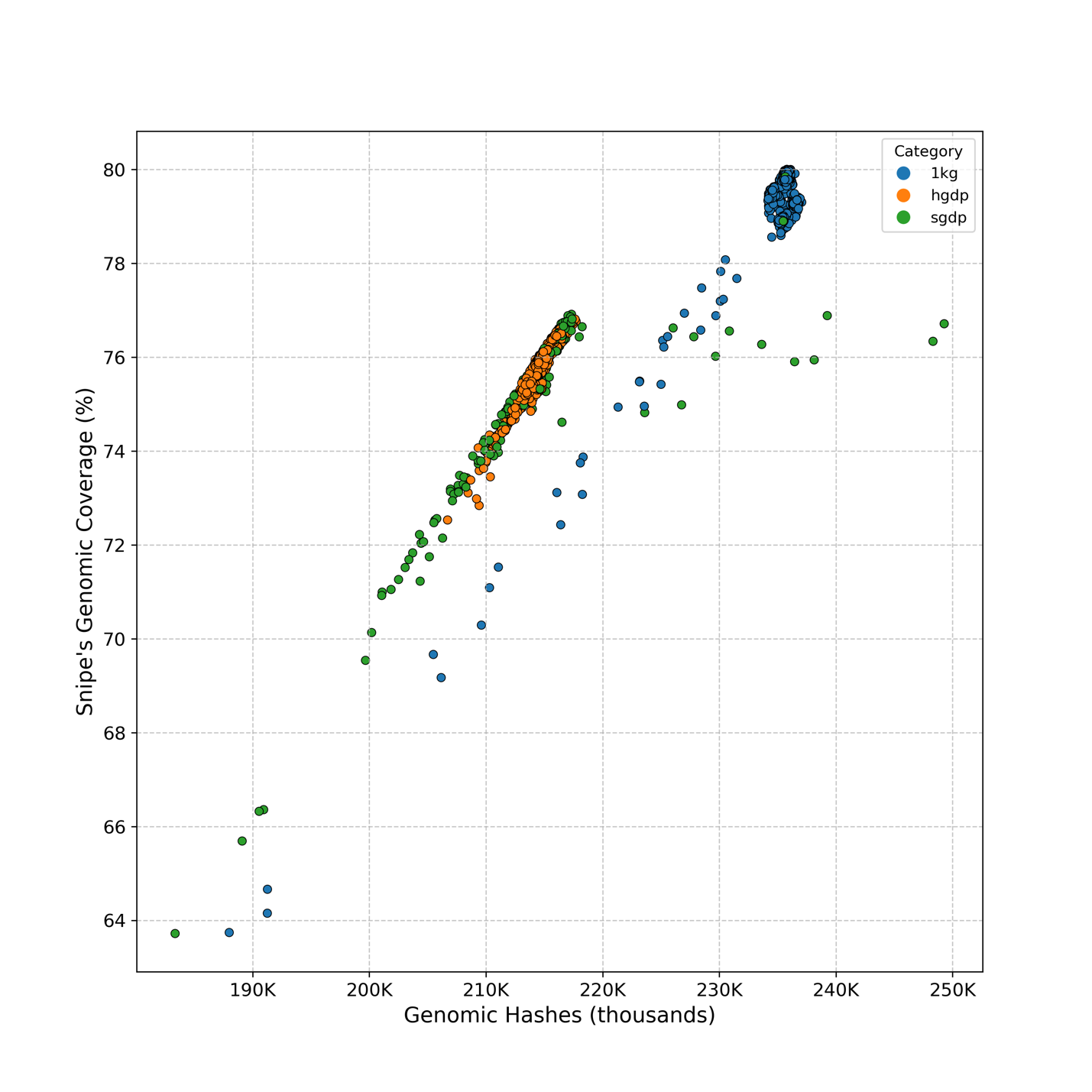


**Supplementary Figure 2: Alignment-free estimation of the human genome coverage in the 1kGP samples**


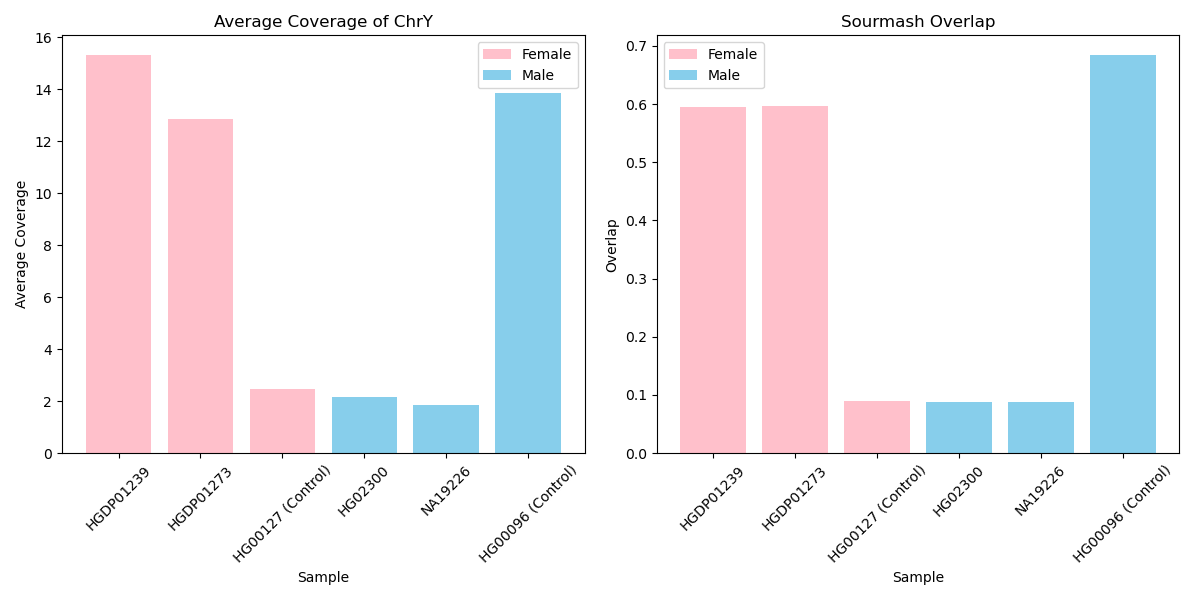


**Supplementary Figure 3:** **Gender discrepancies in four samples of the 1kGP metadata**. The figure shows an agreement between alignment-free (right panel) and alignment-based (left panel) gender detection while disagreeing with the online metadata. On the right, the alignment-free approach identifies the gender by calculating the containment between the sample’s signature and the signature of the reference chromosome Y. These four samples showed unexpected deviation from the mean of its gender containment ratio (0.65 with SD=0.039 and 0.08 with SD=0.003 for males and females respectively). On the left, the average sequencing coverage of chrY was calculated from the cram files. The color of the bars represents the sex as provided in the metadata, with blue bars denoting male and pink bars denoting female. Ideally, blue bars should be larger than pink bars; however, inconsistencies arise due to errors in the metadata.


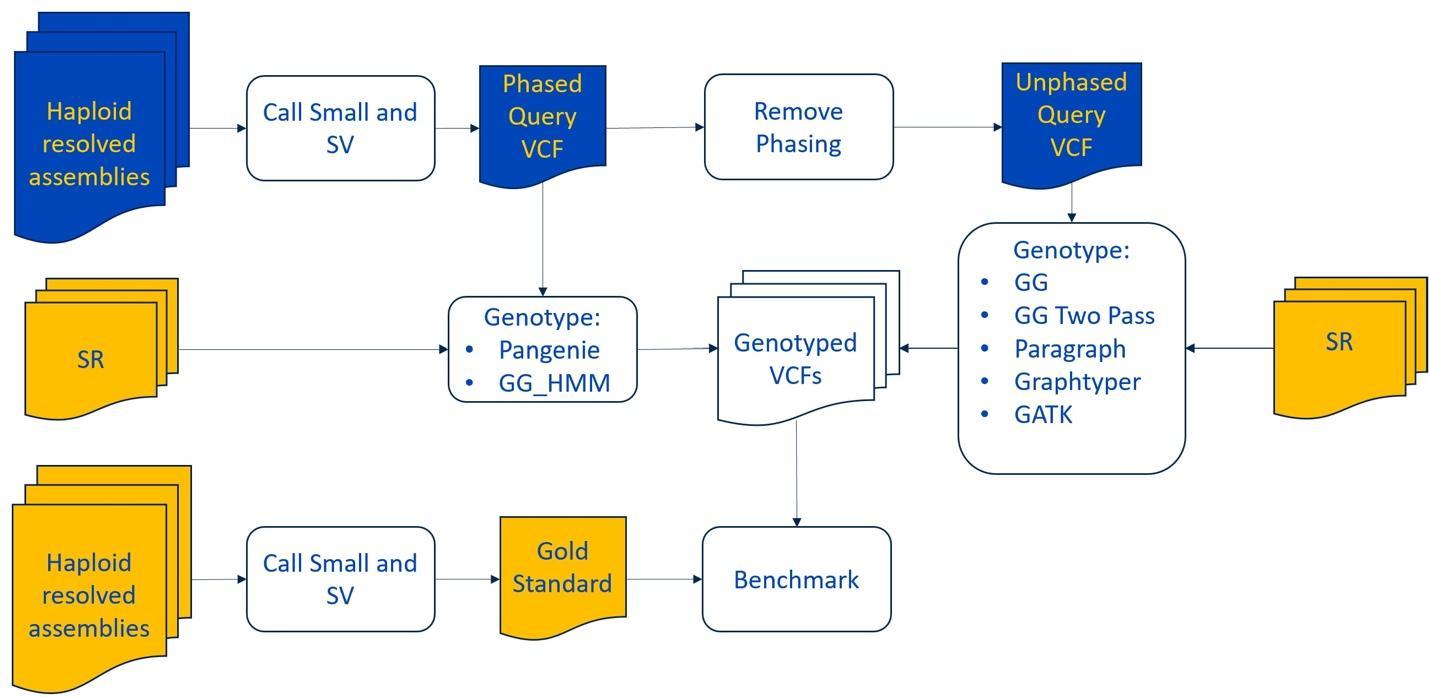


**Supplementary Figure 4.** **Benchmark Genotyping Accuracy Workflow:** The figure represents the workflow for the benchmarking experiment. The gold color represents sample HG00731 and the blue represents the NA12878 sample.


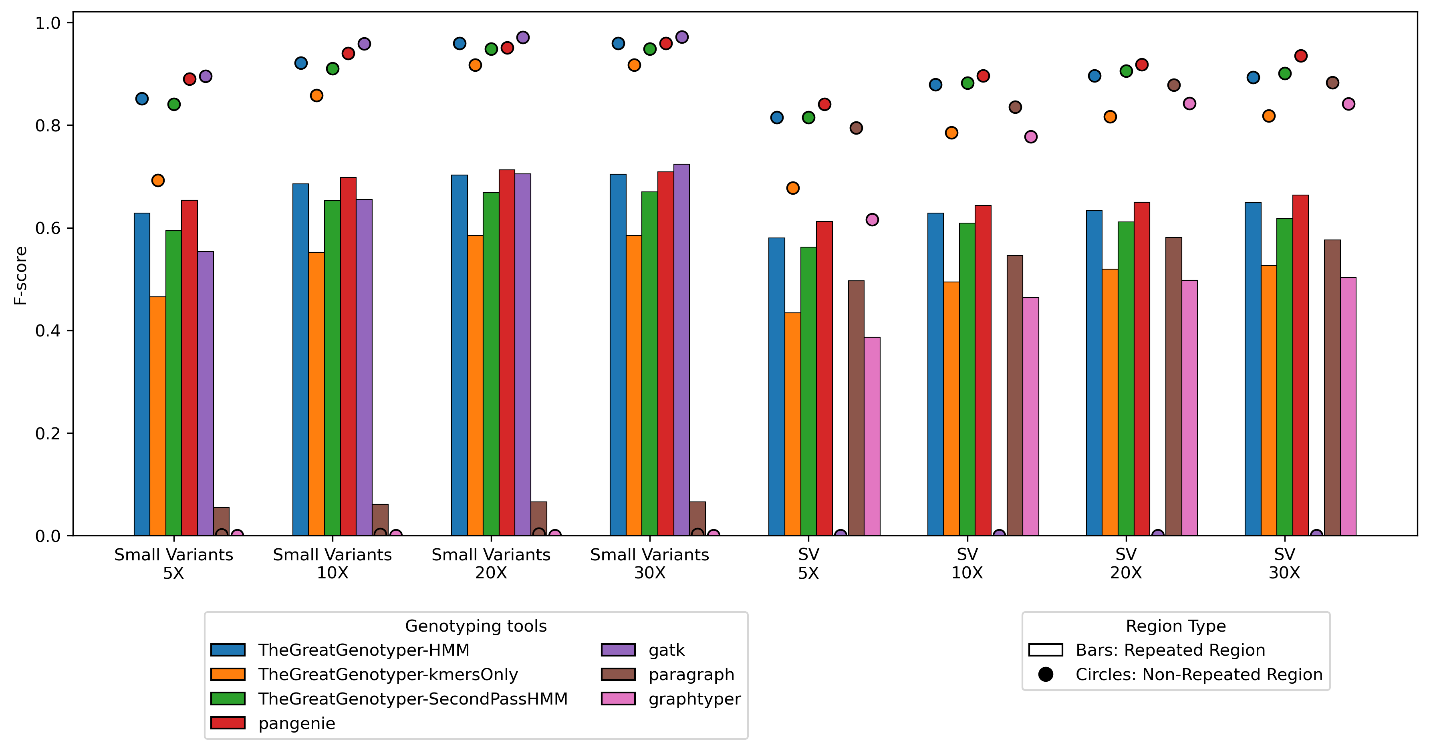


**Supplementary Figure 5.** **Coverage effect on genotyping F-score**: The figure illustrates the effect of coverage on the F-scores of different genotyping methods, differentiating between small variants (under 50 bp) and SV (above 50 bp). F-scores for variants located in repeated regions are shown as bars, while those in non-repeated regions are shown as circles. Additionally, variants are categorized based on the complexity of their genomic location.


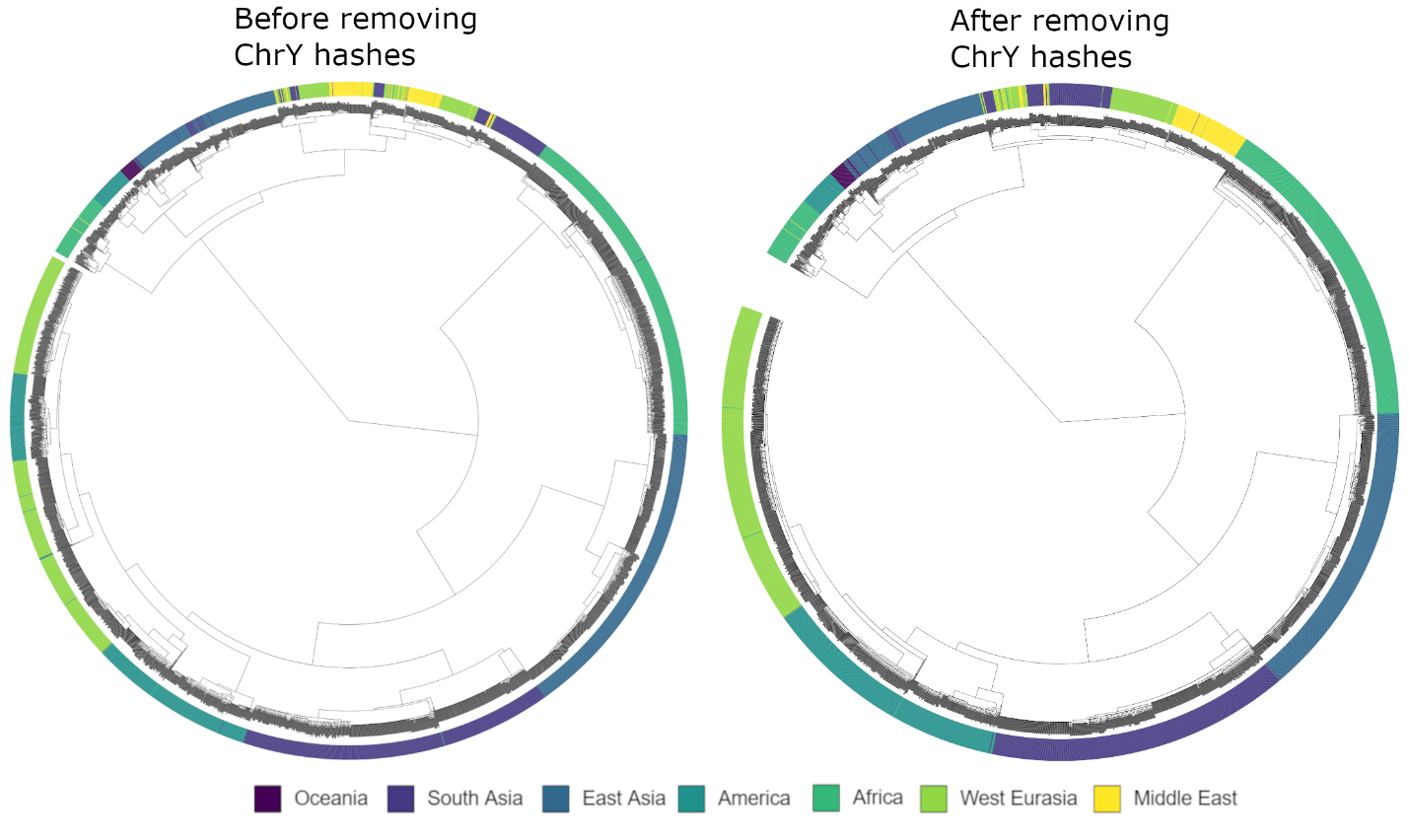


**Supplementary Figure 6. Addressing chrY Bias in Clustering**: The figure depicts two attempts to create a dendrogram for the 4271 samples. The outer circles consist of fine lines, each representing a sample, with the color of the lines signifying the population of the sample as per the metadata. The dendrogram is displayed within these circles, delineating the clusters. Excluding chrY hashes yields more homogeneous clusters, as illustrated in the left dendrogram.


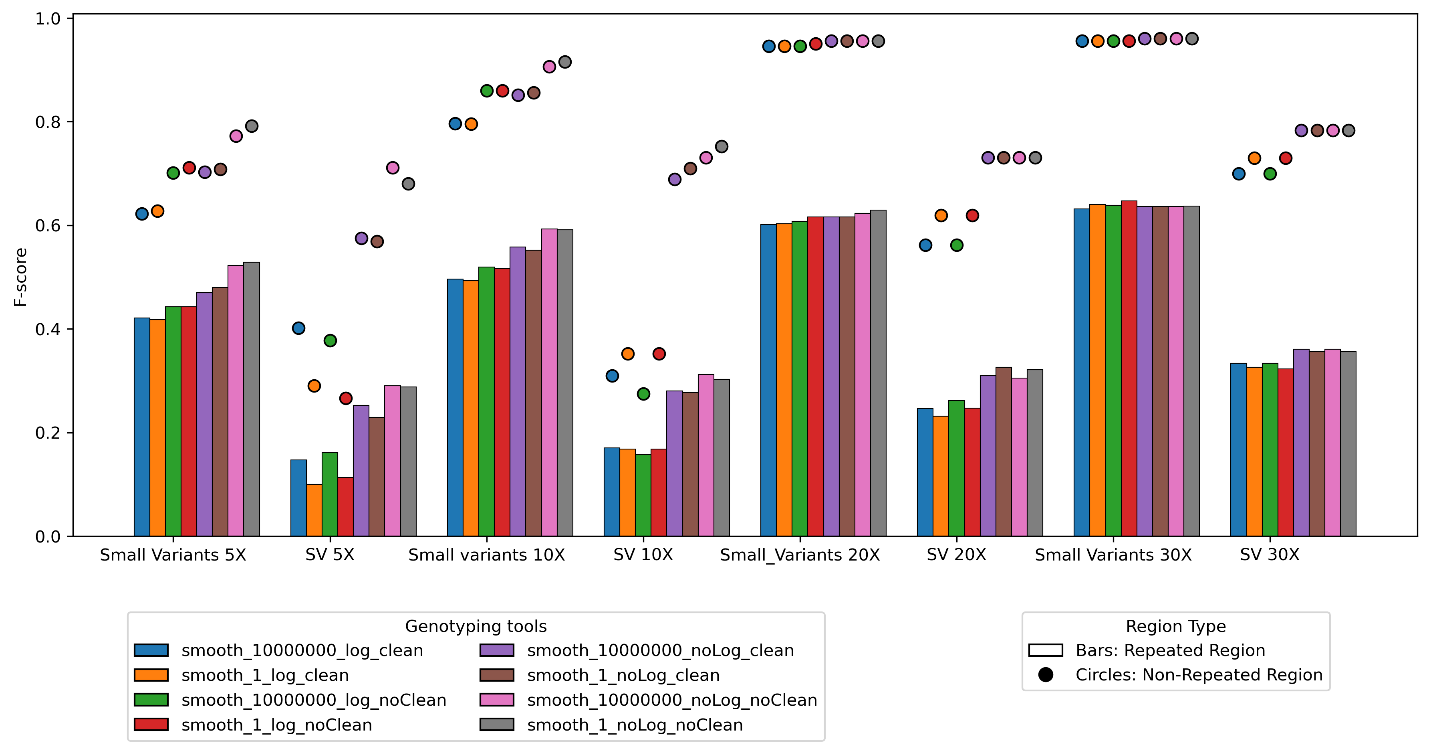


**Supplementary Figure 7.** **F-score comparison between The different parameters of indexing**: Metagraph's preprocessing techniques influence the genotyping f-score. These techniques include *Smoothing Counts*, where only the average kmer count per unitig for each sample(smooth10000000) is retained instead of preserving the individual kmer counts(smooth1); *Log Counts*, which involves saving the log of kmer counts to conserve space; and *Clean*, the error cleaning algorithm in the Metagraph documentation(<https://metagraph.ethz.ch/static/docs/quick_start.html#graph-cleaning>)


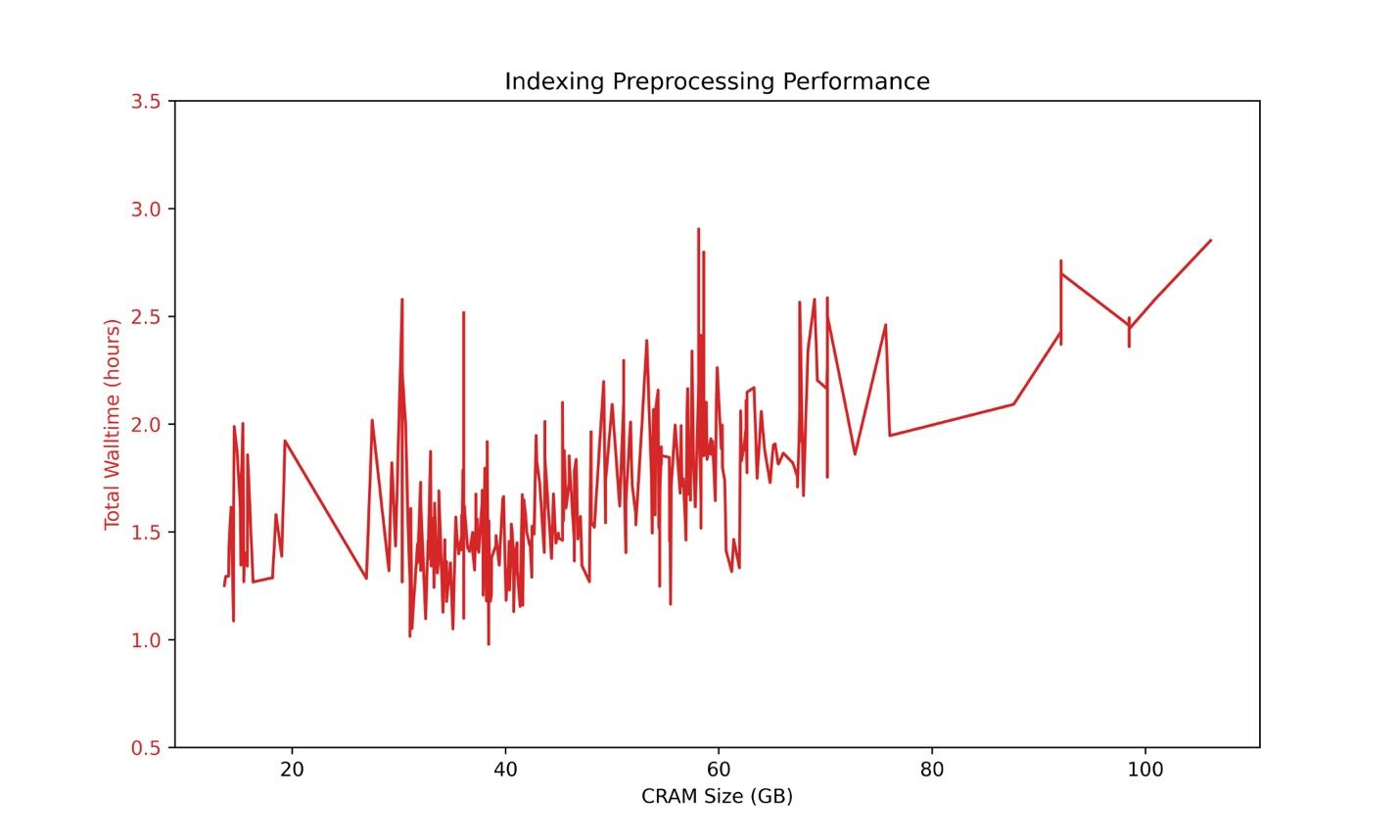


**Supplementary Figure 8: Computational Performance of Preprocessing Steps.** This figure illustrates the runtime and memory usage for preprocessing 1,000 samples. The preprocessing workflow involves three key steps: k-mer counting using KMC, constructing a compact De Bruijn graph with Metagraph, and graph traversal to generate unitigs. The x-axis represents the size of the dataset in CRAM format and the y-axis shows the total wall-clock time (using 16 threads). Note that the tools allow for a configurable memory cap, which in this analysis is set to 20 GB. Selecting different parameters may impact both I/O performance and overall results.


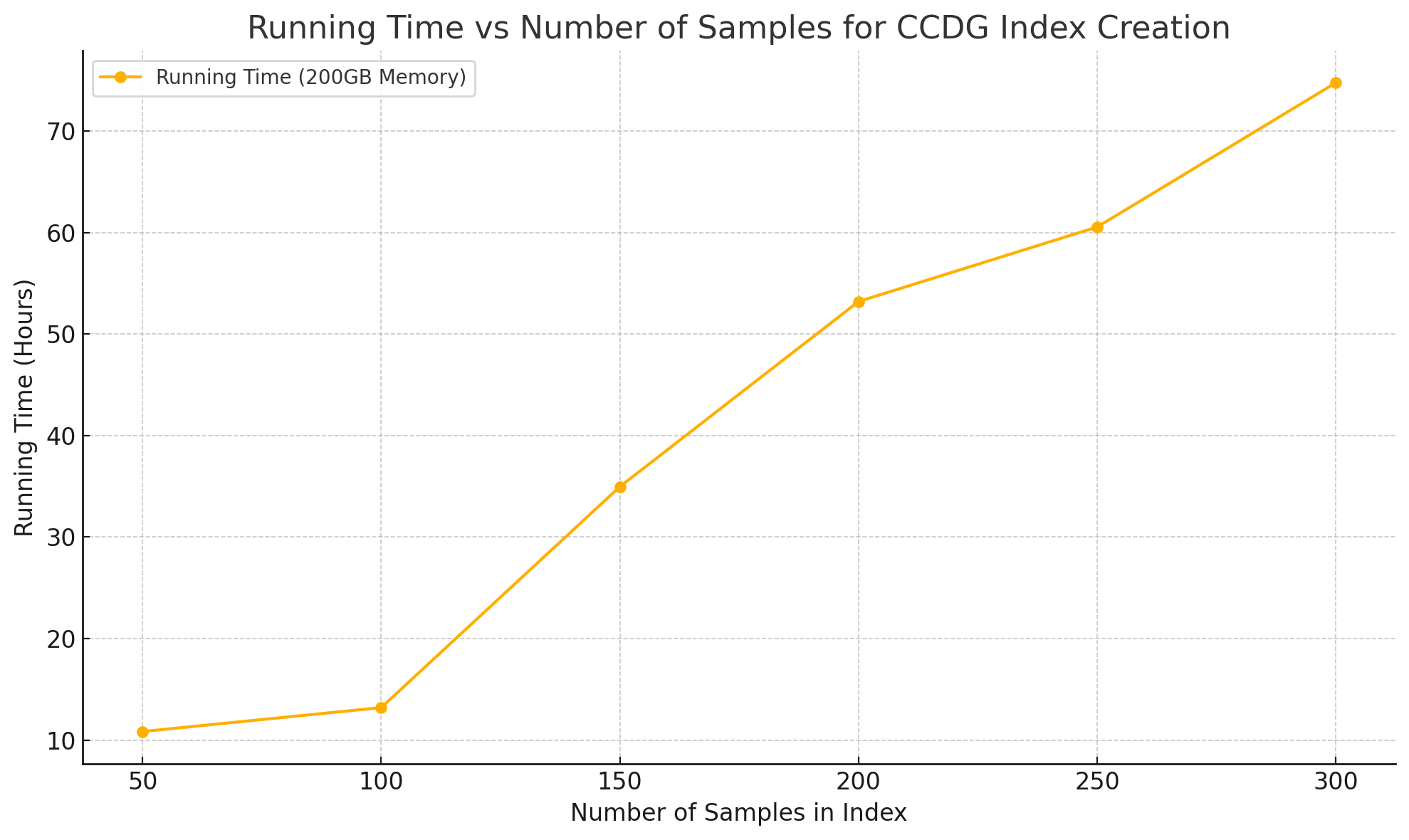


**Supplementary Figure 9: Computational Performance of Creating a CCDG.** This figure illustrates the runtime required to create a CCDG using Metagraph, assuming the preprocessing steps have already been completed. All samples are 30X coverage on average. The experiment was conducted on a server with 32 threads and 200 GB of memory. The primary factor influencing computational cost is the number of samples to be indexed in the CCDG. It is important to note that the workflow is highly parallelizable, and the wall-clock time can be significantly reduced if a high-performance computing (HPC) environment is available, allowing the use of multiple nodes.


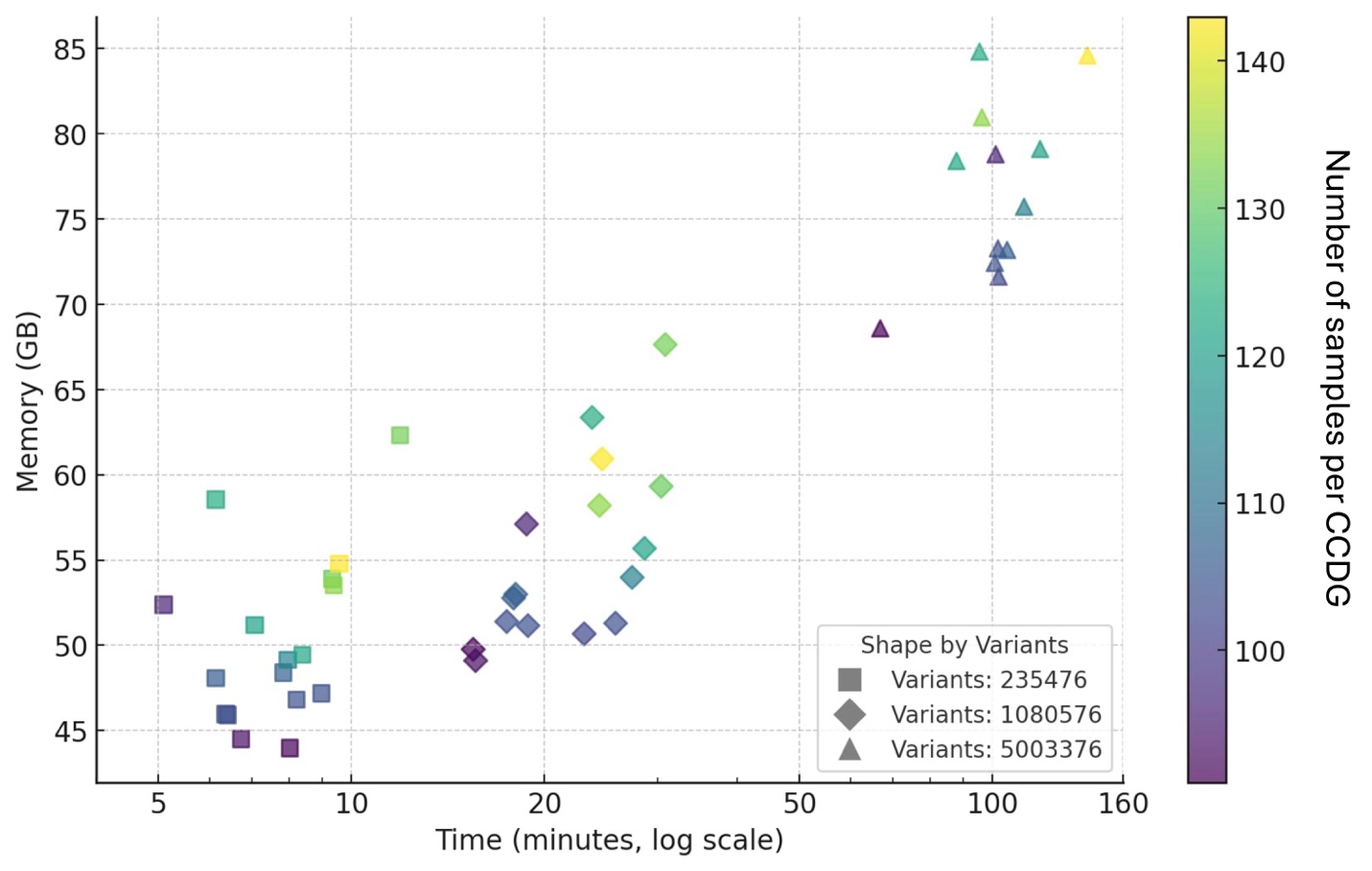


**Supplementary Figure 10: Computational Performance of Initial Genotyping per CCDG.** This figure depicts the wall-clock time and memory usage required to genotype increasing number of variants in multiple sub-indexes with different number of samples per index. The number of variants has the major impact on both time and memory demands compared to the number of samples per index. The experiment was conducted on a server with 32 threads.


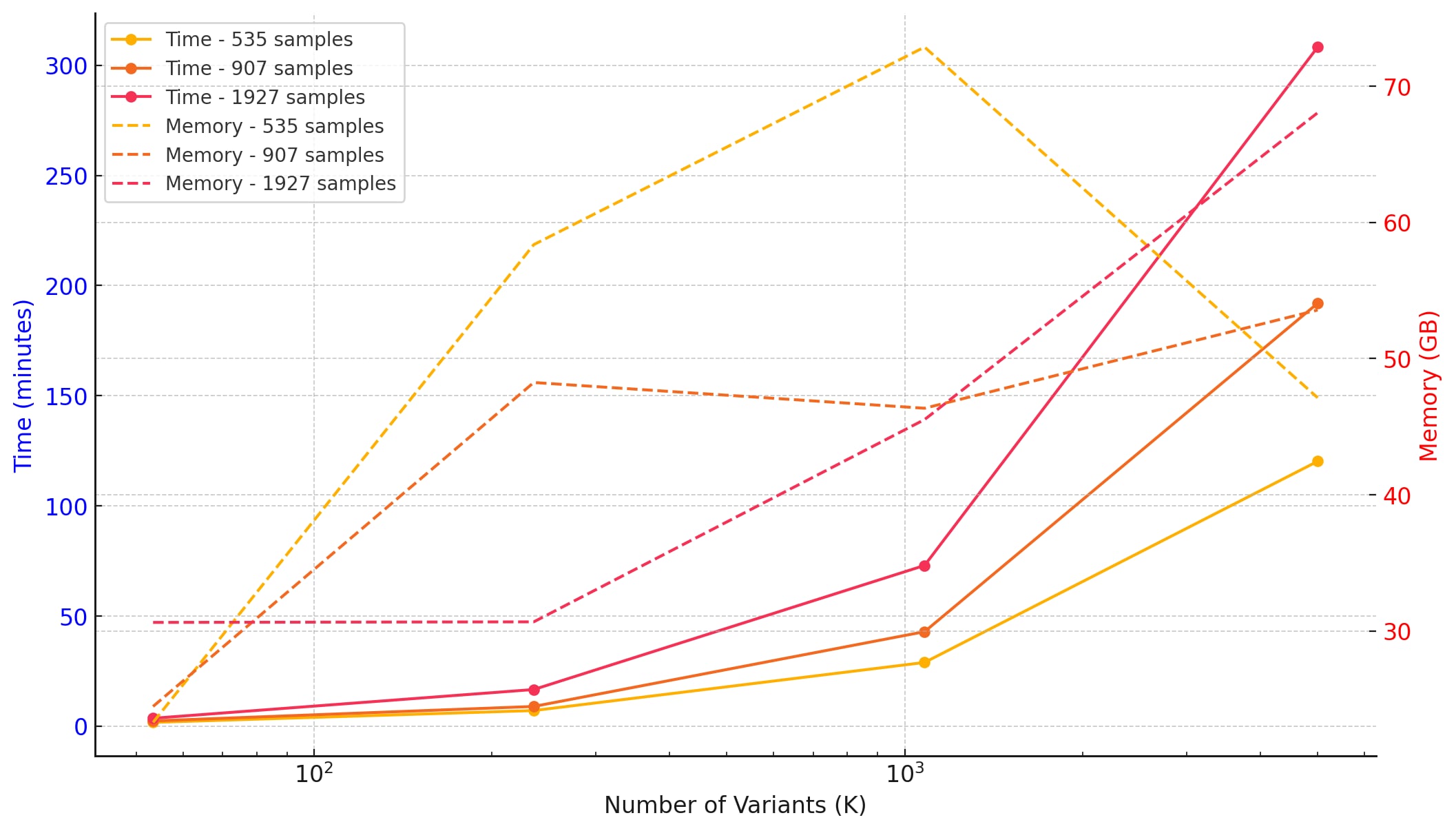


**Supplementary Figure 11: Computational Performance of Population-level filtering and Imputation:** This figure depicts the wall-clock time and memory usage required to aggregate, filter, phase and impute an increasing number of variants in multiple populations of samples. The time increases in a linear fashion with the number of samples but exponentially with the number of variants. On the other hand, the memory consumption does not show such a constant pattern because it depends on the Beagle’s partitioning of the chunk size that can be tweaked by the user. However, it was always less than 100 GB in all our experiments. All experiments were conducted on a server with 32 threads


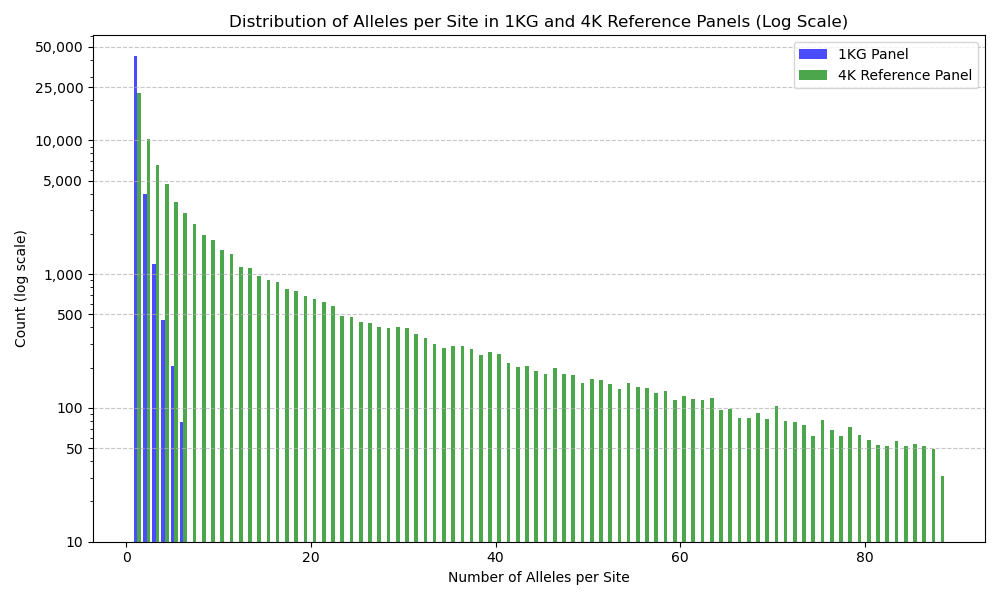


**Supplementary Figure 12**: **Comparison of structural variant allele counts in the 1KG and 4K Reference Panels.** This figure compares the distribution of structural variant allele counts per site in two datasets: the 1KG Panel (blue bars) and the 4K Reference Panel (green bars). The x-axis represents the number of structural variant alleles per site, while the y-axis displays the count of sites on a logarithmic scale. The 1KG Panel shows a narrower distribution with fewer multi-allelic sites, as most sites contain one or two alleles, resulting in a total of 48,497 alleles across all sites. In contrast, the 4K Reference Panel exhibits a broader distribution, with a significant number of sites containing higher allele counts, contributing to a total of 80,275 alleles. This highlights the increased structural variant diversity captured in the 4K Reference Panel


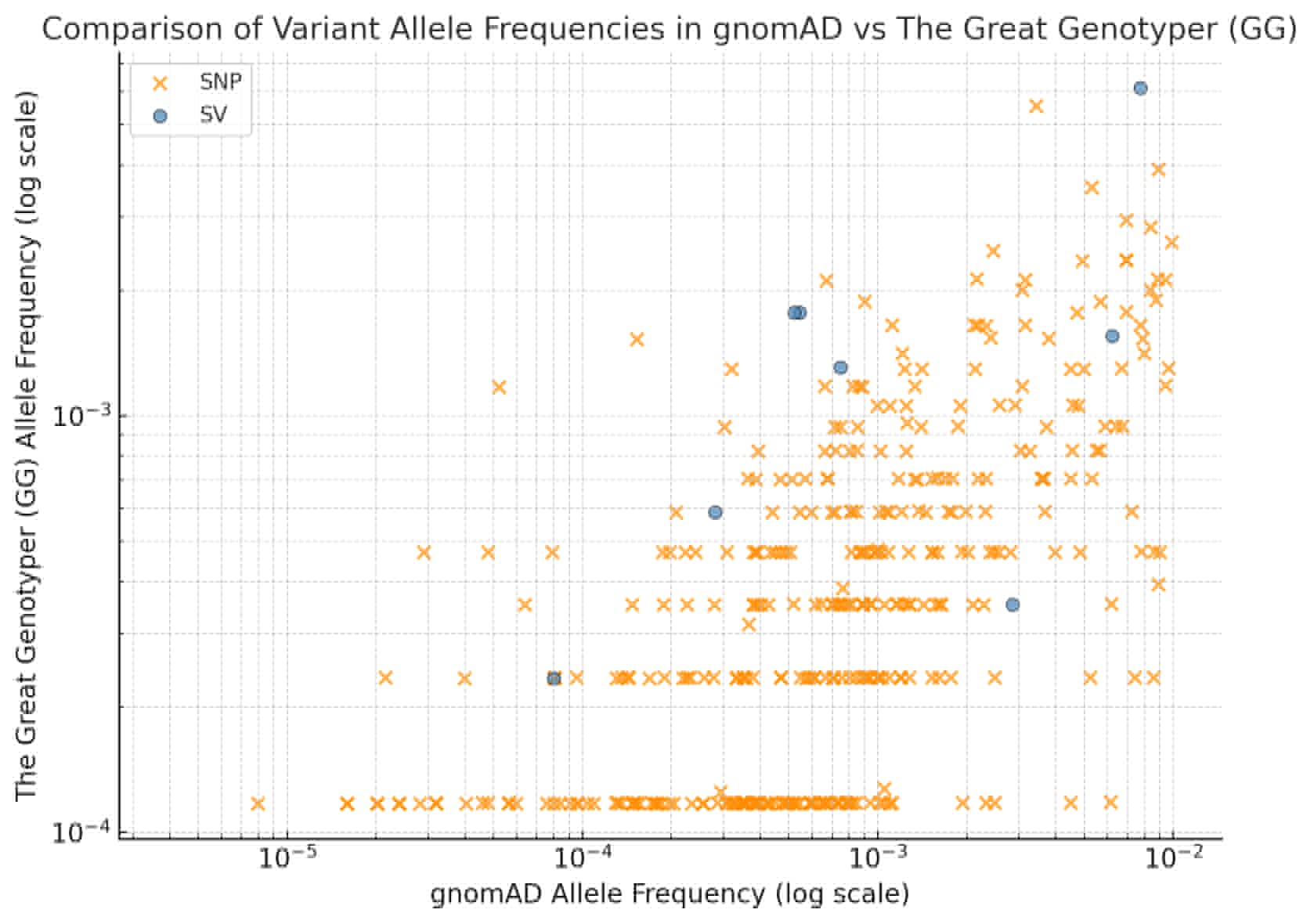


**Supplementary Figure 13: The Great Genotyper (GG) calculates the population allele frequencies of likely-pathogenic variants identified in human genetic studies**. Variants were selected from 14 rare genetic disease studies. A scatter plot shows a linear correlation between their allele frequencies in the gnomAD database (x-axis) versus The GG frequencies in 4,200 short-read whole-genome samples (y-axis) on a log-log scale.

| Variant Type | Count |
| --- | --- |
| SNV | 18.5M |
| Indels(<50 bp) | 7.3M |
| Insertions | 1.5M |
| Deletions | 1.8M |
| Complex | 4.1M |
| SV(>=50bp) | 904.7K |
| Insertions | 85.5K |
| Deletions | 23.4K |
| Complex | 795.8K |

**Supplementary Table 1.** **Variant Counts in HPRC Pangenome** The table details the number of variants from the decomposed VCF of the HPRC pangenome stratified by their type.

| Index Name | Smoothed Counts | Log Counts | Cleaned | CCDG size(GB) |
| --- | --- | --- | --- | --- |
| Smooth10000000_log_clean | Y | Y | Y | 1.42 |
| Smooth10000000_log_noClean | Y | Y | N | 1.59 |
| Smooth10000000_noLog_clean | Y | N | Y | 1.62 |
| Smooth10000000_noLog_noClean | Y | N | N | 1.80 |
| Smooth1_log_clean | N | Y | Y | 1.76 |
| Smooth1_log_noClean | N | Y | N | 1.92 |
| Smooth1_noLog_clean | N | N | Y | 3.00 |
| Smooth1_noLog_noClean | N | N | N | 3.30W |

**Supplementary Table 2.** **Impact of Metagraph Preprocessing on CCDG size:** The table demonstrates how preprocessing methods like Smoothing Counts, Log Counts, and the Clean algorithm alter the size of the final CCDG. Smoothing Counts simplifies kmer data to averages per unitig, Log Counts compresses kmer information logarithmic ally, and Clean removes errors.

| **Metric** | **Great Genotyper** | **PanGenie** | **GraphTyper** |
| --- | --- | --- | --- |
| **Core algorithm** | k-mer-plus-HMM genotyping using a haplotype panel with additional enhancement by population-level genotyping data. | k-mer-plus-HMM genotyping using a haplotype panel. | Read mapping / local realignment to variant graphs (mapping-based). |
| **Main use-case** | Calculation of population allele frequency of a new set of phased or unphased variants in a precomputed index for 1000s of short read samples | Genotype a catalogue of phased variants (e.g. a pangenome) in a new short-read sample(s). | Genotype a catalogue of variants (e.g. from a large population) in a *new* short-read sample(s). |
| **CPU Running time for genotyping new samples (30x coverage sample and 4.5 million variants from the human pangenome on a node with 32 threads)** | **Preprocessing**:  ~1 hour/sample  **Indexing**:  ~ 0.3 hour/sample (indexing 150 samples required 35 hours)    **Genotyping**:  ~ 1 min/sample (genotyping 150 samples required ~2.5 hours) | **Genotyping**: ~1 hour/sample | **Preprocessing** (mapping by BWA-MEM):  ~10.7 hour/sample  **Genotyping**: ~1.2 hour/sample |
| **CPU Running time for re-genotyping pre-processed samples** | ~ 1 min/sample (re-genotyping of 150 indexed samples requires ~2.5 hours) | ~1 hour/sample | ~1.2x hour/sample |

**Supplementary Table 4. Benchmarking Great Genotyper, PanGenie, and GraphTyper** **Regarding Core Algorithms, Applications, and Runtime Metrics**: A single-sample genotyper like Pangenie is likely a better choice for genotyping a new sample for a defined list of variants. On the other hand, GG is designed for population-scale re-genotyping which is best used for quick calculation of population allele frequency of novel variants or genotyping a given population of samples many times for any new set of variants.

* ***Note 1***: Re-genotyping a given sample for the same set of variants is not a practical use case, however, we are using the same set of variants for illustration)

* ***Note 2***: This benchmarking is for illustration only and not meant to be comprehensive. For example, we did not include download time of raw sequencing data. In addition, we did not consider many scenarios like different mapping algorithms which affect the preprocessing time of GraphTyper significantly. Also, the haplotype structure of genotyped variants is likely to affect the imputation steps of GG and PanGenie as well as the complexity of variant graphs needed in GraphTyper. Finally, we are using one computational configuration for the benchmarking, however GG has a modular pipeline which allows users to tailor compute resource usage based on their environment (See Supplementary figures 8,9 and 10).
