# Supplementary material for: The Great Genotyper: a graph-based method for population genotyping of small and structural variants
Source: Gigascience. 2025 Oct 3;14:giaf112. doi: 10.1093/gigascience/giaf112 (PMC12491952; doi:10.1093/gigascience/giaf112)
Supplement: giaf112_GIGA-D-24-00266_Revision_3 [file giaf112_giga-d-24-00266_revision_3.pdf]

GigaScience

The Great Genotyper: A Graph-Based Method for Population Genotyping of Small and Structural Variants

--Manuscript Draft--

Manuscript Number:	GIGA-D-24-00266R3
Full Title:	The Great Genotyper: A Graph-Based Method for Population Genotyping of Small and Structural Variants
Article Type:	Technical Note
Funding Information:	
Abstract:	Long-read sequencing (LRS) enables variant calling of high-quality structural variants (SVs). Genotypers of SVs utilize these precise call sets to increase the recall and precision of genotyping in short-read sequencing (SRS) samples. With the extensive growth in availabilty of SRS datasets in recent years, we should be able to calculate accurate population allele frequencies of SV. However, reprocessing hundreds of terabytes of raw SRS data to genotype new variants is impractical for population-scale studies, a computational challenge known as the N+1 problem. Solving this computational bottleneck is necessary to analyze new SVs from the growing number of pangenomes in many species, public genomic databases, and pathogenic variant discovery studies.<br>To address the N+1 problem, we propose The Great Genotyper, a population genotyping workflow. Applied to a human dataset, the workflow begins by preprocessing 4.2K short-read samples of a total of 183TB raw data to create an 867GB Counting Colored De Bruijn Graph (CCDG). The Great Genotyper uses this CCDG to genotype a list of phased or unphased variants, leveraging the CCDG population information to increase both precision and recall. The Great Genotyper offers the same accuracy as the state-of-the-art genotypers with the addition of unprecedented performance. It took 100 hours to genotype 4.5M variants in the 4.2K samples using one server with 32 cores and 145GB of memory. A similar task would take months or even years using single-sample genotypers.<br>The Great Genotyper opens the door to new ways to study SVs. We demonstrate its application in finding pathogenic variants by calculating accurate allele frequency for novel SVs. Also, a premade index is used to create a 4K reference panel by genotyping variants from the Human Pangenome Reference Consortium (HPRC). The new reference panel allows for SV imputation from genotyping microarrays. Moreover, we genotype the GWAS catalog and merge its variants with the 4K reference panel. We show 6.2K events of high linkage between the HPRC's SVs and nearby GWAS SNPs, which can help in interpreting the effect of these SVs on gene functions. This analysis uncovers the detailed haplotype structure of the human fibrinogen locus and revives the pathogenic association of a 28 bp insertion in the FGA gene with thromboembolic disorders.
Corresponding Author:	Tamer Mansour, Ph.D.<br>University of California Davis<br>Davis, UNITED STATES
Corresponding Author Secondary Information:	
Corresponding Author's Institution:	University of California Davis
Corresponding Author's Secondary Institution:	
First Author:	Moustafa Shokrof
First Author Secondary Information:	
Order of Authors:	Moustafa Shokrof
	Mohamed Abuelanin
	C. Titus Brown

Powered by Editorial Manager® and ProduXion Manager® from Aries Systems Corporation

	Tamer Mansour, Ph.D.
**Order of Authors Secondary Information:**	
**Response to Reviewers:**	We would like to thank the editor and the reviewers for their constructive comments and valuable feedback throughout the review process. In this round, we have addressed the remaining issues based on the editorial guidance provided during our last communication with Dr. Hans Zauner.<br><br>A) CPU Times for All Genotypers<br>Following the suggestion to provide a breakdown of CPU times for all genotypers, we have included a new Supplementary Table 4. This table presents benchmarking results for the Great Genotyper, PanGenie, and GraphTyper, including details of their core algorithms, application steps (e.g., indexing and genotyping), and runtime metrics. In addition, we updated the relevant paragraph on page 4, line 156, to clarify that the initial indexing step in the Great Genotyper makes one-time genotyping slower than with the other tools, and that its speed advantage becomes evident only in repeated genotyping scenarios. The new table is now cited in that paragraph as well as on page 7, line 294.<br><br>B) Variants Called by Manta but Missing in the 1K Panel<br>As explained in our last communication, we are now sharing the Truvari analysis outputs for all three panels tested in our evaluation. The following changes were made to the manuscript:<br>•Page 6, line 234: We added a pointer for readers to refer to the ‘Data and Code Availability’ section.<br>•Page 9, line 371: We included the following text in the ‘Data and Code Availability’ section: “The VCF output files of Truvari’s pairwise analysis comparing SVs on Chr22—imputed using the 4k reference panel, the 1000 Genomes panel, and SV calls from Manta—are available at: https://farm.cse.ucdavis.edu/~tahmed/truvari.tar.gz”<br>•Figure 3 caption (Panel D): We updated the description to: “Panel D shows the precision and recall of variant imputation using the 4k reference panel in comparison to imputation using the 1000 Genomes panel and SV calling using Manta.”<br><br>We appreciate the opportunity to improve our manuscript and look forward to your feedback.
**Additional Information:**	
**Question**	**Response**
Are you submitting this manuscript to a special series or article collection?	No
**Experimental design and statistics**<br><br>Full details of the experimental design and statistical methods used should be given in the Methods section, as detailed in our Minimum Standards Reporting Checklist. Information essential to interpreting the data presented should be made available in the figure legends.<br><br>Have you included all the information requested in your manuscript?	Yes
**Resources**	Yes

A description of all resources used, including antibodies, cell lines, animals and software tools, with enough information to allow them to be uniquely identified, should be included in the Methods section. Authors are strongly encouraged to cite Research Resource Identifiers (RRIDs) for antibodies, model organisms and tools, where possible.<br><br>Have you included the information requested as detailed in our Minimum Standards Reporting Checklist?	
**Availability of data and materials**<br><br>All datasets and code on which the conclusions of the paper rely must be either included in your submission or deposited in publicly available repositories (where available and ethically appropriate), referencing such data using a unique identifier in the references and in the “Availability of Data and Materials” section of your manuscript.<br><br>Have you have met the above requirement as detailed in our Minimum Standards Reporting Checklist?	Yes

Powered by Editorial Manager® and ProduXion Manager® from Aries Systems Corporation

Manuscript Click here to access/download;Manuscript;main.tex

The Great Genotyper: A Graph-Based Method for Population Genotyping of Small and Structural Variants

Moustafa Shokrof [1,2] Mohamed Abuelanin [1,2] C.Titus Brown [1]
Tamer A. Mansour [1,3]

[1] Department of Population Health and Reproduction, School of Veterinary Medicine, University of California, Davis, CA, USA
[2] Computer Science Graduate Group, University of California, Davis, CA, USA
[3] Department of Clinical Pathology, School of Medicine, Mansoura University, Mansoura, Egypt

July 19, 2025

1 Abstract

Long-read sequencing (LRS) enables variant calling of high-quality structural variants (SVs). Genotypers of SVs utilize these precise call sets to increase the recall and precision of genotyping in short-read sequencing (SRS) samples. With the extensive growth in availability of SRS datasets in recent years, we should be able to calculate accurate population allele frequencies of SV. However, reprocessing hundreds of terabytes of raw SRS data to genotype new variants is impractical for population-scale studies, a computational challenge known as the N+1 problem. Solving this computational bottleneck is necessary to analyze new SVs from the growing number of pangenomes in many species, public genomic databases, and pathogenic variant discovery studies.

To address the N+1 problem, we propose The Great Genotyper, a population genotyping workflow. Applied to a human dataset, the workflow begins by preprocessing 4.2K short-read samples of a total of 183TB raw data to create an 867GB Counting Colored De Bruijn Graph (CCDG). The Great Genotyper uses this CCDG to genotype a list of phased or unphased variants, leveraging the CCDG population information to increase both precision and recall. The Great Genotyper offers the same accuracy as the state-of-the-art genotypers with the addition of unprecedented performance. It took 100 hours to genotype 4.5M variants in the 4.2K samples using one server with 32 cores and 145GB of memory.

The Great Genotyper opens the door to new ways to study SVs. We demonstrate its application in finding pathogenic variants by calculating accurate allele frequency for novel SVs. Also, a premade index is used to create a 4K reference panel by genotyping variants from the Human Pangenome Reference Consortium (HPRC). The new reference panel allows for SV imputation from genotyping microarrays. Moreover, we genotype the GWAS catalog and merge its variants with the 4K reference panel. We show 6.2K events of high linkage between the HPRC's SVs and nearby GWAS SNPs, which can help in interpreting the effect of these SVs on gene functions. This analysis uncovers the detailed haplotype structure of the human fibrinogen locus and revives the pathogenic association of a 28 bp insertion in the FGA gene with thromboembolic disorders.

2 Introduction

Maya Angelou eloquently stated, "In diversity, there is beauty and there is strength." This principle is particularly relevant to genomics studies, emphasizing the importance of exploring genetic diversity across large cohorts and

populations. Such research is crucial for advancing our understanding of evolution [1, 2], genetic adaptations [3], and gene-disease associations [4, 5]. Genetic diversity originates from various mutations, including single nucleotide variants (SNVs), small insertions and deletions (less than 50 base pairs), and structural variants (greater than 50 base pairs). Notably, structural variants (SVs) enhance genomic diversity fifteen times more than SNVs [6] and significantly affect gene function [7]. However, SVs are understudied compared to smaller variants due to the limitations of short-read sequencing (SRS), which often yields high false positive rates and inconsistent recall, varying from 10% to 70% [8]. In contrast, long-read sequencing (LRS) provides more reliable precision and recall rates [8] and is used in both mapping [9, 10, 11] and assembly-based approaches [12], the latter of which helps mitigate mapping biases to a linear genome reference. Despite its advantages, LRS remains prohibitively expensive for comprehensive population-scale analysis, and the volume of LRS data available still pales in comparison to that of SRS. As a result, there is a pressing need to develop computational techniques that utilize the precise variant discovery capabilities of LRS while maximizing the extensive data produced by SRS.

To effectively utilize the abundant short-read sequencing data available, while addressing the limitations of short-read SV callers, specialized genotypers analyze the presence and genotype of SVs, whether identified through variant calling from SRS or LRS, in SRS samples [13, 14, 15, 16, 17]. Tools such as Paragraph [14] and Graphtyper2 [16] realign reads to a variation-aware graph, minimizing mapping bias and determining genotypes from this realignment. Pangenie [17] uses k-mers specific to all potential alleles to genotype phased variants from pangenomes, minimizing mapping bias. Furthermore, Pangenie integrates genotyping and imputation, utilizing the phasing information from the pangenome to infer genotypes in regions lacking coverage, thereby achieving superior performance compared to other SV genotypers. Unlike these single-sample genotypers, muCNV utilizes population data to refine genotyping by modeling read mapping statistics across multiple samples, enhancing genotyping accuracy [15].

SV genotypers generally achieve higher recall and precision compared to direct variant calling in SRS samples. For instance, Huddleston et al. [18] used LRS to analyze SVs in two human genomes and found that 90% of these SVs were missing in the 1000 Genomes call set, yet 61% could still be genotyped using SRS. Recent population-scale studies have therefore adopted a combined approach of variant calling and genotyping: initially, variants are identified from a few LRS samples or numerous SRS samples, and then the identified SVs are merged and genotyped in a larger SRS cohort [19]. For instance, Kirsche et al. [20] used Paragraph [14] to genotype variants from 31 LRS samples in a cohort of 1.3k SRS samples from the 1000 Genome Project (1kGP) [21]. Similarly, Graphtyper2 was employed to build graphs from SVs detected in 50k Icelandic SRS samples [16] or 2k dog SRS samples [22], which were then re-genotyped using the same SRS samples to improve recall. With the same concept in mind, the Human Pangenome Reference Consortium (HPRC) [23] applied Pangenie to genotype the pangenome variants in 3.2k SRS samples from 1kGP [21]. Similarly, Goo Jun et al. [24] used MuCNV to jointly genotype TopMed SVs in 139k SRS samples. These genotypers enable large-scale population genotyping of gene catalogs, pangenomes, and candidate disease-associating variants.

The current SV genotypers, while fast and scalable, face significant challenges at the population level. These genotypers require downloading and reprocessing all the raw SRS data to genotype even a single new variant, a demand that is increasingly impractical. This issue exemplifies a computational challenge known as the N+1 problem [25]. In today's era of extensive sequencing, new lists of variants emerge daily, and a reliable estimation of their allele frequencies is important for interpretation. For instance, the number of pangenomes for humans [23, 26] as well as numerous other species [27, 28, 29, 30] is increasing. Similarly, databases like dbVar [31], genomeAD [32], TopMed [33], and ClinVar [34] are constantly expanding their variant collections. The N+1 challenge also affects disease gene discovery studies in probands [35]. LRS can produce phased, high-quality SVs, and identifying pathogenic variants involves filtering out common variants and focusing on rare ones. However, matching these variants in public databases poses challenges, and the reliability of allele frequencies in SV catalogs is dubious when calculated in small or distinct subpopulations or when using methods with low recall. Therefore, solving this computational bottleneck is crucial to optimize the usage of genomic data for advancing precision medicine and enhancing our understanding of genetic diversity.

A new trend in genomics [36, 37] involves preprocessing raw sequencing data to create searchable indexes that

can be directly utilized by downstream applications. One state-of-the-art tool in this field is Metagraph, highly efficient software for indexing the k-mer content of massive sequencing datasets using a Counting Colored de Bruijn Graph (CCDG). A CCDG encodes k-mers along with an array that annotates their counts in each sample, preserving essential genotyping information in a compact format [38].

Building on this foundation, we introduce The Great Genotyper, an alignment-free genotyping tool designed for both structural and small variants. The Great Genotyper efficiently partitions raw sequencing data from thousands of samples then indexes them into CCDGs using Metagraph. The CCDG is used to genotype any set of variants, eliminating the need for raw data and solving the N+1 problem. The Great Genotyper leverages the Pangenie's genotyping model and population-derived data to enhance the genotyping accuracy.

As a use case, we used The Great Genotyper to partition and index 183 TB of raw sequences from 4.2K human samples into an 867 GB partitioned CCDG. The index is used to genotype 26.8 million variants from the human pangenome. Additionally, we demonstrated how these population-level genotypes can serve as an imputation panel for structural variants and enable the annotation of structural variants based on their linkage to nearby GWAS SNPs.

3 Result

3.1 The Great Genotyper: A Workflow for Genotyping Small and Structural Variants in Thousands of Short-Read Samples

The Great Genotyper solves the problem of genotyping a new list of variants in a given population (i.e., the N+1 problem) by deploying two independent workflows. The first is an indexing workflow that performs all the heavy lifting once by creating a CCDG using raw SRS to represent the population (Figure 1A). Once created, the CCDG can be reused by a population genotyping workflow (Figure 1B) to genotype a pangenome, phased variants, or unphased variants in the cohort of SRS samples.

The indexing workflow preprocesses raw sequencing datasets to extract k-mer content, perform alignment-free QC, and partition samples. An individual CCDG is generated for each partition. The genotyping workflow utilizes each sub-index independently to maximize parallelization. The outputs from genotyping one or more sub-indexes are eventually combined for final population-level QC and imputation.

The design of The Great Genotyper minimizes computational time while avoiding memory bottlenecks when genotyping thousands of samples, especially when distributed across high-performance computing (HPC) or cloud systems. Preprocessing of raw data can be executed independently for each sample and the runtime is primarily influenced by sample size, averaging approximately 2 hours per sample on a node with 16 threads and 20 GB of memory (Supplementary Figure 8). The time of a sub-index creation depends on the number of samples, as illustrated in Supplementary Figure 9. For instance, creating an index for 150 samples with an average coverage of 30X requires approximately 35 hours on a node with 32 threads and 200 GB of memory. In contrast, genotyping a sub-index depends primarily on the number of variants in a sub-index of a recommended range of samples. However, genotyping 5 million variants in a sub-index with 150 samples needs less than 2.5 hours on a server node with 120 GB of RAM and 32 threads (Supplementary Figure 10). Since sub-indexes are independent, their generation and genotyping can be distributed across multiple nodes, enabling efficient scaling. This design also facilitates the addition of new samples by creating a new independent sub-index. The final step which involves aggregating all VCFs and imputing missing genotypes depends primarily on Beagle software, which is known for its efficiency and scalability (Supplementary Figure 11).

In this study, the Great Genotyper was used to construct a CCDG for 140 human populations (Figure 1A and Supplementary Figure 1). This involved downloading 4.2K high-coverage (30x) whole-genome sequencing (WGS) samples from the 1000 Genomes Project (1KGP) [21], the Human Genome Diversity Project (HGDP) [39], and the Simons Genome Diversity Project (SGDP) [40]. Initial QC revealed seven samples with unexpectedly low genome coverage and four discrepancies between reported and predicted sex (Supplementary Figures 2 and 3). Subsequently,

a dendrogram of sample sketches was generated (Figure 1A.2), identifying 29 partitions, each encompasses between 100-350 closely related samples (Figure 1A.3).

Processing the 183 TB of raw data required approximately 21 days of preprocessing time using 16 nodes, each with 16 cores and 20 GB of RAM. The creation of 29 sub-indexes took an additional 10 days, using four 32-core servers with 200 GB of RAM each. The resulting sub-index sizes ranged from 16 GB to 68 GB, with a total combined size of 867 GB.

Building upon the CCDGs created in the indexing workflow, the genotyping workflow (Figure 1B) empowers the analysis of any variant list across all samples without requiring raw reads or mapping. It begins with three key inputs: a list of pre-generated CCDGs, a reference genome and a variant list (phased or unphased). Depending on needs, three different workflows can be chosen: A) k-mer-based workflow: Efficiently genotypes unphased variants. B) Hidden Markov model (HMM) workflow: Handles both genotyping and imputation for phased variants. C) Two-pass workflow: Genotypes and imputes unphased variants, leveraging population information to determine their phase and impute missing data.

Both the k-mer-based and HMM workflows start by extracting k-mers unique to the variant regions and querying their count data for all samples within the CCDGs (Figure 1B). The k-mer-based workflow determines initial genotypes by comparing the counts of unique k-mers to the average sample coverage for each sample. This identifies variants present in each sample without relying on phasing information. In contrast, the HMM workflow tackles phased variants by genotyping and imputing them using the Hidden Markov Model (HMM) implemented in Pangenie [17]. This enables the imputation of genotypes in regions with low coverage or complexity. Following initial genotyping, both workflows undergo a two-step refinement. The first step is to filter low-quality genotypes after comparing the genotype qualities for each variant across all samples. The second step utilizes Beagle [41, 42] to statistically impute low-confidence genotypes and phase the resulting variants.

The third workflow is a pipeline to genotype and impute unphased variants. It starts by running the k-mer-based workflow to create a reference panel using the input variants and samples in the CCDGs. This reference panel is then used to phase the input variants. After that, the HMM workflow is employed on the phased variants to obtain more precise genotypes in indexed population.

3.2 Achieving Population Genotyping in a Matter of Hours with no Decrease in Accuracy

The performance of the Great Genotyper was evaluated using its k-mer-based workflow (for unphased variants) and HMM-based workflow (for phased variants). After indexing the 4.2K WGS samples, the Great Genotyper genotyped 4.5 million variants across all samples in approximately 100 hours, utilizing 32 cores and 145 GB of memory, as shown in Figure 2A. For comparison, we ran PanGenie and GraphTyper2 on raw FASTQ data for a single sample. PanGenie required about 1 hour, while GraphTyper2 took roughly 12 hours on the same machine. Notably, PanGenie processes raw reads directly, whereas GraphTyper2 requires read alignment, which we performed using the BWA-MEM algorithm. Extrapolating these runtimes, both tools would require months to genotype all 4.2K samples for each new variant set. This comparison shows that, due to its one-time indexing cost, the Great Genotyper may not be faster than single-sample genotypers when analyzing a single variant set. However, it offers substantial speed advantages when genotyping large cohorts repeatedly across multiple variant sets (see Supplementary Table 4 for benchmarking of the Great Genotyper, PanGenie, and GraphTyper2 regarding core algorithms, applications, and runtime metrics).

For benchmarking of precision and recall of the Great Genotyper with other state-of-the-art genotypers, we genotyped SVs and small variants derived from the NA12878 haploid-resolved assemblies using the 30x SRS of HG00731 (See Methods and Supplementary Figure 4 for the design of benchmarking and Figure 2 for the detailed results).

The Great Genotyper's HMM and Pangenie exhibit superior F-scores for phased SVs, achieving 0.91 in non-repetitive regions. Paragraph and the k-mer-based workflow follow closely with F-scores of 0.88 and 0.87 for unphased

SVs. Intriguingly, the two-pass workflow accurately predicts the phasing information, boosting the F-score back to 0.91. In contrast, Graphtyper trails with an F-score of 0.80. The challenges increase in repetitive regions, where variability in results is more pronounced. Here, Pangenie and the HMM workflow score 0.63 and 0.61, respectively, followed by Paragraph and the k-mer-based workflow at 0.55. However, the two-pass workflow enhances the k-mer-based approach's F-score to 0.6, while Graphtyper lags with an F-score of 0.48.

For small variants, GATK leads, achieving F-scores of 0.97 and 0.70 in non-repetitive and repetitive regions, respectively. The Great Genotyper's HMM and Pangenie are close behind with F-scores of 0.95 in non-repetitive areas. The k-mer-based workflow scores 0.93, improving slightly to 0.94 with the two-pass workflow. In repetitive regions, Pangenie matches GATK's 0.70 F-score, while the HMM workflow slightly trails at 0.69. The k-mer-based workflow struggles in these regions and scores 0.6 but is improved to 0.65 by the two-pass workflow. Overall, the Great Genotyper consistently demonstrates competitive genotyping accuracy compared to Pangenie across most scenarios, and it represents the most accurate option for genotyping unphased SVs with the two-pass workflow.

Sequencing depth impacts the genotyping accuracy, as depicted in Figure 2B2. Notably, all genotypers exhibit reduced accuracy at sequencing depths of 10x and 5x. Genotypers that incorporate phasing information, such as The Great Genotyper's HMM and two-pass workflows, as well as Pangenie, show the smallest decrease in accuracy. For instance, the accuracy of SV genotyping by The Great Genotyper's HMM and Pangenie at 5x coverage drops by 8% and 9% in non-repetitive regions, and 7% and 5% in repetitive regions, respectively. The k-mer-based workflow experiences a decrease of 14% and 9%, which the two-pass model returns to 7% and 5% in non-repetitive and repetitive regions, respectively. Last, Graphtyper's accuracy diminishes by 12% and 22% in non-repetitive and repetitive regions, respectively.

The reduction in sequencing depth from 30x to 5x similarly affects the accuracy of small variant genotyping in both non-repetitive and repetitive regions. Pangenie exhibits the smallest accuracy decline, by 7% and 5%, followed by The Great Genotyper's HMM with 11% and 7%, and GATK with 8% and 17%. The k-mer-only model suffers a significant drop of 22% and 11%, but this is mitigated by the two-pass model to 10% and 7% in non-repetitive and repetitive regions, respectively.

3.3 Facilitating Population Studies for Small and Structural Variants

3.3.1 The Great Genotyper can help to find pathogenic variants

Filtering common variants is a widely used strategy in disease association studies. ClinVar, a public database, catalogs genomic variations in humans and their impact on health [34]. As a proof of concept, the k-mer-based workflow is applied to genotype the ClinVar database variants in the 4k samples of the CCDG index. Consistent with expectations, almost all pathogenic variants exhibit zero allele frequency in this healthy population, whereas benign variants display a broader range of frequencies (Figure 3A). This demonstrates that calculating allele frequencies for a list of suspected variants in this indexed cohort is a reliable metric for prioritizing rare variants in studies of their pathogenic potential.

An additional experiment was conducted to highlight the impact of efficient re-genotyping at the population scale. The Great Genotyper (GG) was used to genotype 790 likely pathogenic variants identified in 14 recent human genetic studies across a cohort of 4,000 individuals. GG successfully confirmed the population allele frequencies of 749 variants with reliable Hardy-Weinberg Equilibrium (HWE) significance threshold of $p > 10^{-6}$, showing a strong linear correlation with their frequencies in the gnomAD database (Supplementary Table 3 and Supplementary Figure 13).

3.3.2 Generation of 4k reference panel by Genotyping HPRC Variants in 4K Samples

The current HPRC pangenome, consisting of 88 haplotypes, decomposes into a phased VCF containing 26.8 million variants, as previously described [23] (see Supplementary Table 1 for a detailed summary of variant types). The HMM workflow is used to genotype these variants in the prebuilt CCDG. The resulting output is a phased VCF of

the HPRC variants in the indexed 4K samples, creating a new 4K reference panel. Principal Component Analysis (PCA) on the genetic variation within this 4k reference panel confirms the expected distribution of populations studied in the 1kGP, paving the way to generate cost-efficient similar panels for several other species (Figure 3B). Subsequent sections will explore how this panel can facilitate various genomic applications.

3.3.3 Impute SV by using the 4k reference panel

Genotype imputation is a statistical method that predicts unobserved genotypes using reference sequences, thereby enhancing the density and scope of genetic analyses at reduced costs. This technique is especially valuable in increasing the power and consistency of genetic studies, including genome-wide association studies (GWAS) and fine-mapping efforts [43]. The 4k reference panel may replace the panel generated by the 1kGP project [44] while enabling the imputation of structural variants (SVs). In this section, we demonstrate the precision and recall of imputing both small and structural variants using the 4k reference panel. Initially, pseudo-microarray variant calls are generated using the HG002 sample from the Genome in a Bottle (GIAB) project [45] by extracting variants at sites used in the Illumina Infinium OmniExpress-24, simulating microarray genotyping. The 4k reference panel is then employed to impute both small and structural variants. For benchmarking purposes, the 1kGP reference panel is used for imputing small variants and SVs. Also, SV calling from 30x SRS using Manta serves as another reference point. The output VCFs are compared against gold standard GIAB datasets using hap.py (v0.3.12) [46] for small variants, and truvari (v3.5.0) [47] for SVs (see the 'Data and Code Availability' section). The 4k reference panel exhibits commendable precision and recall for the imputation of both types of variants, as depicted in figure 3D. When compared to the 1kG reference panel, it displayed some reduced precision compensated by an increase in recall for SNPs and indel imputation. Conversely, the 4k reference panel shows remarkable recall of SV (86%) which surpasses the recall of not only the 1kGP reference panel but even SV calling from 30x SRS using Manta. The precision of 4k reference panel is also higher than the 1kGP reference but still obviously lower than the precision of variant calling. These results highlight how the 4k reference panel can be leveraged to augment microarray genotypes with common SVs.

We further investigated the reasons behind the performance differences between the 1kGP reference panel and the 4k reference panel. Our analysis revealed that the number variant sites and their alternative haplotypes in the pangenome (used to create the 4k reference panel) are much higher than those in the 1kGP panel. Specifically, the pangenome cover 80K variant sites while the 1kGP reference panel has only 48K 1kGP reference panel. Moreover, 72% of the sites in the HPRC pangenome have multiple alternative alleles with an average 10.6 alternative alleles per position, whereas only 22% of the sites in the in the 1kGP panel have multiple alternative alleles with an average 1.2 alleles per position (see Supplementary Figure 12). The more accurate representation of polymorphic regions in the 4k reference panel enabled the imputation tool to identify the correct haplotypes more effectively.

3.3.4 Fine Mapping of GWAS SNPS using SVs from the 4k reference panel

The 4k reference panel provides detailed insights into the structure of common haplotypes composed of small and structural variants. In particular, it allows the exploration of linkage disequilibrium (LD) between SVs and neighboring variants known to be associated with phenotypic changes. We initiate our investigation by annotating the SVs in the 4k reference panel using AnnotSV (v3.3.6) [48]. This reveals that approximately 463K SVs affect gene structures. Proceeding further, we compute the pairwise LD for each of these variants with all the variants located within a 1MB window surrounding them. Our analysis indicates that 91K SVs exhibit a strong association with a neighboring variant, having an r2 value greater than 0.8.

We utilize the identified associations to illuminate potential causal variants in GWAS studies. Among the 91K SVs, 6,253 are found in strong linkage with GWAS SNPs. We compiled a table that includes these SVs, their annotations, associated GWAS SNPs, and other relevant metadata (see the 'Data and Code Availability' section). This table should be a valuable resource elucidating the phenotypic effects of common SVs and help pinpoint some causal variants of the traits examined in these GWAS studies. We are using a Sankey plot in Figure 3E as a flow

diagram between the different categories of these associations based on the size SV, the impact of either the GWAS SNP or SV on coding regions, and the ability of the SV to cause a frameshift. Notably, 722 of these SVs impact the coding regions of genes, with 415 causing frameshift mutations.

We explore a specific example from our list in figure 3C, focusing on the Human fibrinogen locus on chromosome 4. This 50-kilobase region includes three fibrinogen genes: the central FGA gene encodes the alpha chain, flanked by FGB and FGG encoding the beta and gamma chains, respectively [49]. Our reference panel shows an insertion of 28 bp at chr4:154584089 (dbSNP: rs148317511; ClinVar: RCV000247066) in a high linkage (r2=0.98) with rs6050-C; a missense mutation in FGA associating with venous thromboembolism [50, 51, 52, 53, 52] and chronic thromboembolic pulmonary hypertension [53, 54]. The insertion is reported in ClinVar as a benign variant. Surprisingly, further digging in the literature shows that the variant was once known as the Taq I polymorphism because it created an additional restriction site for Taq I [55]. The allele was found to enhance the stability of FGA mRNA in vitro [51]. This was explained by the ability of the insertion to oppose the suppressive effect of has-miR-759 on the 3 UTR of FGA [54]. These findings suggest that the ClinVar information on the variant should be revised.

Interestingly, our panel is able to capture the haplotype structure of the fibrinogen locus and shows how the 28bp insertion fits in. For example, rs6050 is known to be in high linkage with rs7681423; SNP upstream to FGG and a peak of association with γ' Fibrinogen. Both SNPs are known to have no significant association with total fibrinogen levels and no linkage with rs1800789; SNP in FGB shows the strongest association with total fibrinogen level, but not with γ' fibrinogen [56]. The panel confirms these relationships between the three SNPs and shows that the insertion allele has some linkages (r2=76) to rs7681423 and no linkage to rs1800789. Also, the panel shows a unique haplotype (r2=96) of the insertion and rs2070011-A; an allele of CFA's promoter causing higher expression of the gene. This haplotype is different from the haplotype of rs6050 and rs7681423.

4 Discussion

The Great Genotyper serves as a practical solution for population genotyping at massive scales. It provides the ability to genotype a new set of variants, whether small or structural, in thousands of SRS samples in just a matter of hours. The Great Genotyper can do this by providing a novel solution for the chronic N+l problem by eliminating the need to download and process terabytes of raw sequencing data. Instead, the Great Genotyper operates using a prebuilt CCDG, effectively decoupling intensive data preprocessing from the actual genotyping process. Therefore, the Great Genotyper is best suited for regenotyping scenarios which allow for quick calculation of population allele frequencies for any new set of variants. Also, it enables instant genotyping of the same dataset samples for any new set of variants. With this design, genotyping a large population once for a given set of variants using The Great Genotyper is still computationally on par with leading state-of-the-art genotyping tools. However, thanks to its pre-built index, re-genotyping the same cohort for any new set of variants becomes a much easier computational task (Supplementary Table 4).

To add another dimension to the scalability of The Great Genotyper, it adopts a distributed design that allows its CCDG to be composed of multiple sub-indexes. Therefore, new samples can be appended easily as an additional sub-index without the need to recompute the entire structure. In terms of input, the Great Genotyper is versatile; it accepts any set of phased or unphased variants, along with the reference genome. The outcome is the phased genotypes of all input variants in the indexed samples. In this manuscript, 183 TB of SRA files for 4K human SRS samples are indexed to generate an 867 GB CCDG to enable unprecedented efficiency in calculating allele frequencies of any list variants in the human population. As a proof of concept, the index is used to genotype the HPRC pangenome variants as an example of phased variants, genotyping all unphased ClinVar variants, and candidate variants in 14 studies of human genetic diseases.

The Great Genotyper does not sacrifice quality for scalability. On the contrary, the scalability empowers the Great Genotyper to jointly genotype thousands of samples, which, in turn, enhances the genotyping quality even more. K-mer-based genotypers such as Nebula and Pangenie have previously demonstrated the potential of k-mers

for precise genotyping. They leverage the specificity of variant-specific k-mers, using shifts in the counts of these k-mers as indicators to genotype the variants. The Great Genotyper reinforces this approach, considering the counts of these k-mers across an entire population of samples. This innovation facilitates the calculation of a confidence measure for each genotype based on the collective population data. Furthermore, the tool is equipped to impute missed genotypes through a two-tiered approach. Initially, imputation is rooted in the phasing information of the variants, either provided as input or derived from the large cohort genotypes. Subsequently, the Great Genotyper integrates Beagle, leveraging the high-confidence genotypes within the population to further impute genotypes. This dual-phase imputation process ensures that the Great Genotyper can deliver performance on par with Pangenie, even if some data is compromised during the k-mer count preprocessing while indexing to enable better data compression as described in Supplementary Figure 7.

The enhanced accuracy and scalability of the Great Genotyper paves the way for valuable downstream applications in genomics. For instance, accurate allele frequencies can now be directly derived from sequences rather than merging information from sparse studies or variation databases that rely on variant calling in SRS studies. Such accurate determination of allele frequencies can play a pivotal role in pinpointing causal variants in disease-gene discovery studies (Supplementary table 3). This is even more important for species that lack human-level variance annotation. Furthermore, simultaneous genotyping and phasing of common variants enables dramatically improved resolution for understanding the haplotype structure within and across populations. As an example, genotyping the HPRC pangenome variants in 4k samples produces what we call "the 4k reference panel (4kRP)". We show how the 4kRP can be used to impute common SVs with a recall rate that surpasses some short-read callers like Manta.

Taking our analysis further, we explore the 4kRP for SVs in high LD with known GWAS SNPs. We limit our focus to 91K SV variants impacting gene structures. Intriguingly, we discover that approximately half of these SVs exhibit strong associations with at least one GWAS SNP. We are optimistic that our findings will contribute to a deeper comprehension of the relationship between genotype and phenotype concerning these structural variants.

Although the Great Genotyper is effective in generating high-quality genotypes for both small and structural variants, it does have certain limitations. First, some variants cannot produce specific k-mers because the k-mers from the alternate sequences may also be present in other parts of the genome. Such variants cannot be genotyped precisely by k-mer-based approaches. This limitation, however, is partially offset through imputation. Furthermore, genotyping copy number variants is beyond the capabilities of the current version of the Great Genotyper. While it is not an insurmountable challenge, it requires development of a dedicated genotyping model. Another constraint is that the Great Genotyper utilizes two separate imputation models, as they are implemented in two distinct tools, Pangenie and Beagle. A unified model tailored specifically for imputing genotypes using the k-mers in the CCDG could both enhance the accuracy and boost the performance.

The Great Genotyper opens many doors for future genomic applications. Creating more CCDGs to represent specific subpopulations or individuals exhibiting specific traits, like autism, is crucial for understanding the role of genomics in these cohorts. Moreover, while most population studies have been conducted on humans [57], this approach is applicable to many other organisms. The Sequence Read Archive (SRA) [58] is a vast reservoir of short-read samples for non-human organisms. Generating CCDGs for these samples will facilitate population-scale studies for other species.

The current CCDG for the human population, and the additional CCDGs to be created for other cohorts, are invaluable resources with potential applications that extend beyond genotyping. For instance, variants can be directly called from the graph using methods such as Corticall [59]. Additionally, it can aid in subsetting pangenomes by selecting segments of the pangenome that have k-mers present in a specific population, thereby creating a more streamlined pangenome tailored to that population. We encourage the community to explore and uncover more ways to harness the extensive genomic diversity revealed by the CCDG.

5 Conclusion

The Great Genotyper can transform population genotyping into a routine task using a flexible CCDG representation of populations. Its scalability allows the improvement of genotyping quality by using population information. The tool's practicality aids in expanding variant lists into broader dimensions, revealing complex genomic details. We demonstrate its potential in applications such as creating SV imputation panels, finding SV associations with variants from databases like the GWAS catalog, and accurately calculating population allele frequencies. The CCDG, comprising 4.2K human samples, contains a vast genomic variation spectrum, accessible through The Great Genotyper or other methods, leading to enhanced genomic insights. Producing more CCDGs for additional cohorts or species will further optimize the use of existing SRS samples.

6 Data and Code Availability

The code for The Great Genotyper is publicly available on GitHub at the following URL: `https://github.com/dib-lab/TheGreatGenotyper`. The benchmarking code used in our study can also be found on GitHub at this URL: `https://github.com/dib-lab/TheGreatGenotyper_benchmark`. The indexes used in our project are hosted on our server and can be accessed at this URL: `https://farm.cse.ucdavis.edu/~tahmed/GG_index/`.

Additionally, the workflow for building CCDGs using Metagraph is available at: `https://github.com/dib-lab/TheGreatGenotyper/tree/master/DatabaseBuilder`. The workflow for general-purpose pangenome genotyping can be found at: `https://github.com/dib-lab/TheGreatGenotyper/tree/master/pangenome_genotyping`.

We have also provided several use cases, which can be found at this URL: `https://github.com/dib-lab/TheGreatGenotyper_usecases`. The genotyped pangenomes are available at this URL: `https://farm.cse.ucdavis.edu/~mshokrof/4k_reference_panel/`. The VCF output files of the Truvari's pairwise analysis comparing SVs of Chr22 imputed using the 4k reference panel and the 1000 Genome panel, and SV calling using Manta can be found at this URL: https://farm.cse.ucdavis.edu/ tahmed/truvari.tar.gz The LD list and the GWAS SV Associations can be found at this URL: `https://farm.cse.ucdavis.edu/~mshokrof/GWAS_associations/`. Lastly, the ClinVar genotyped data can be accessed at this URL: `https://farm.cse.ucdavis.edu/~mshokrof/The_great_genotyper_clinvar/`.

7 Methods

7.1 Design overview and foundational software

The Great Genotyper is built on the foundation of multiple powerful tools including Metagraph [38], for creating compressed, searchable indexes (CCDG); Pangenie [17], for its advanced haplotype-aware genotyping capabilities; Beagle [41, 42], recognized as one of the best imputation tools; Sourmash[60] and Snipe [61], which provides a fast and efficient method for quality checking and partitioning. In this section, we will highlight the key features of these tools

7.1.1 MetaGraph

MetaGraph [38] is a software designed for indexing billions of k-mers using compressed data structures. At its core is the Counting Colored Compact De Bruijn Graph (CCDG), which extends the traditional De Bruijn graph structure. Nodes represent unique k-mers, and edges capture k-1 overlaps between them, with each node annotated by an array containing k-mer counts across individual datasets. This design supports efficient sequence-based queries or graph traversal, returning both the datasets containing the sequence and the associated k-mer counts

7.1.2 Pangenie

PanGenie [17] genotypes phased small and structural variants in SRS samples using a k-mer-based approach. The method begins by extracting a set of k-mers unique to the variants targeted for genotyping, ensuring specificity in variant representation. Genotyping is performed using a Hidden Markov Model (HMM) inspired by the Li-Stephens haplotype copying model [62]. This model calculates emission probabilities by comparing the observed k-mer counts in the sample to the expected counts for each variant haplotype. The likelihood of a genotype is proportional to the match between these counts, with penalties applied for mismatches or missing k-mers. If insufficient k-mer support exists for a specific variant, the model imputes the genotype by propagating information from nearby variants on the same haplotype. This integration of local context helps address uncertainties in the data, ensuring more accurate genotype calls even in regions of low coverage or complex variation. The genotyping model assigns a confidence measure to each output genotype by calculating the likelihoods of all possible genotypes and selecting the one with the highest probability. Confidence is determined by the difference between the highest probability and the next highest, with a larger difference indicating greater confidence.

7.1.3 Beagle

Beagle [41, 42] is a tool for phasing population genotypes without requiring a reference panel, provided the population size is sufficiently large. Additionally, Beagle imputes missing genotypes statistically by modeling linkage disequilibrium using the Li-Stephens Hidden Markov Model (HMM), thereby generating a reference panel suitable for imputing and phasing other samples. Beagle competes favorably with state-of-the-art solutions in terms of processing time and accuracy, efficiently handling datasets with hundreds of thousands of samples. Its capabilities have enabled the construction of reference panels for large-scale projects such as UK Biobank and TOPMed [42].

7.1.4 Sourmash, Snipe and kSpider

Sourmash [60] is a tool designed for comparing genomes and sequencing datasets, with a particular emphasis on metagenomics. It creates compact FracMinHash sketches, which are probabilistic representations of the k-mer content in each dataset. Unlike traditional approaches that select a fixed number of hash values, FracMinHash uses a fraction-based sampling method, selecting hash values based on a predefined proportion of the hash function's output. This approach allows for more precise and efficient comparisons of genomic datasets. Snipe [61] is a quality control tool that uses an alignment-free approach to compare the sourmash sketches of next-generation sequencing datasets against a target reference, producing comprehensive quality metrics, including accurate calculations of sequence depth and coverage, contamination, sex determination, and genetic variance. kSpider [63] is tool that supports lightweight clustering of thousands of Sourmash sketches, enabling efficient partitioning based on their sequence content, before MetaGraph indexing.

7.1.5 The Great Genotyper Design

The Great Genotyper is designed to address the unique challenges of population genotyping, which differ significantly from single-sample approaches. Processing thousands of samples introduces scalability challenges, particularly in balancing memory requirements and runtime, but also offers opportunities to leverage population-level information. To solve these challenges, The Great Genotyper employs two independent workflows. The indexing workflow performs the heavy computational tasks upfront, creating a CCDG from raw SRS to represent the population. This CCDG can then be reused by the population genotyping workflow to efficiently genotype any new list of variants.

The indexing workflow starts by preprocessing of raw SRS to quantify, error-trim and summarize their k-mer content by kmc [64] and Metagraph [38]. In the next step, a lightweight pipeline is deployed for alignment-free QC and partitioning of input samples using Snipe [61] and kSpider [63]. Please see 7.2 for more details. Finally, each partition is indexed by Metagraph to generate a CCDG. The Great Genotyper tailors the indexing workflow of

Metagraph to minimize the index size without sacrificing the genotyping accuracy. For example, logarithmic scaling of k-mer counts and graph simplification steps which can reduce the size of the index were avoided to prevent accuracy degradation. Further details on indexing parameters are discussed in 7.3. Instead, memory requirements were addressed by splitting the index into multiple sub-indexes that could be processed independently in a map-reduce fashion, with results combined to make population-level decisions.

For the genotyping workflow, The Great Genotyper builds on the C++ codebase of Pangenie (a single-sample genotyper) [17] and implements new logic to tackle population-level challenges and opportunities. Pangenie's original design parallelizes computation by chromosome and stored intermediate data in memory. In contrast, The Great Genotyper is designed to parallelize around samples instead of chromosomes, with the ability to write intermediate data to disk. This enables scaling across distributed systems and allows for controlled memory usage. In addition, Pangenie calculates k-mer counts and estimates genome coverage from these counts for a single input sample before genotyping. On the other hand, these sample-level statistics are calculated during the indexing step in the Great genotyper to be reutilized with any new genotyping task. Moreover, The Great Genotyper has a novel module that make uses of the population-level information to filter out low quality genotypes and utilizes Beagle's state-of-the-art imputation and phasing algorithms to increase the recall in the final output. A details of the genotyping algorithm in The Great Genotyper is discussed in 7.4.

Lastly, we developed a Snakemake workflow to integrate all the modules described above into the indexing and genotyping processes, ensuring ease of use (see Data and Code Availability).

7.2 Short Read Samples Preprocessing and Partitioning

Upon the download of each sample, kmc [64] is used for k-mer counting with a minimum count of 3 to filter out singletons and doubletons, which are likely sequencing errors. In addition, Metagraph [38] is utilized to identify the unitigs and retain only the average k-mer count per unitig, thus smoothing k-mer counts. This smoothing reduces the size of the k-mer counts to about one-tenth while maintaining high genotyping accuracy (see below). Subsequently, alignment-free quality control is done using Sourmash [60] and Snipe [61]. This process begins by downsampling raw sequences into representative summary sketches (i.e. FracMinHash sketches calculated using Sourmash). A sourmash sketch is created for each sample using a k size of 51 and a subsampling scale of 10k, which entails keeping a single hash for every 10,000 k-mers. A similar sketch at the same scale is created for the GRCh38 reference genome. Snipe intersects both signatures to generate approximate estimates of the genome coverage and sequencing depth as well as sex confirmation (Supplementary Figures 2 and 3). Subsequently, kSpider [63] calculated pairwise similarities between all samples based on their sourmash sketches. To alleviate skew from the sex chromosomes, the sequence hashes of chrY are subtracted from all sketches. Hierarchical clustering is employed using the Scipy library [65] to construct a dendrogram that can visualized (see Supplementary Figure 6) by iTOL [66]. From the dendrogram, clusters are extracted into separate partitions of closely related samples to minimize the genetic diversity per partition and hence the final index size enhancing scalability for large datasets.

7.3 Determining The Best Indexing Parameters

We investigated the influence of sample preprocessing on genotyping accuracy to determine the best parameters for optimal results. Multiple CCDGs were generated from sub-samples of the HG00731 SRS at sequencing depths of 5x, 10x, 20x, and 30x. Each CCDG was constructed using a different set of parameters, which are summarized in Supplementary Table 2, along with the final sizes of the CCDGs. Benchmarking was done as described in Supplementary Figure 4 and later in the methods.

Results in Supplementary Figure 7 and Table 2 indicate that preprocessing methods do not impact samples with coverage exceeding 20x. For coverages of 10x and 5x, logging the counts is the most influential, significantly decreasing both the F-score and the final CCDG size. On the other hand, smoothing leads to a nominal drop in the F-score but notably reduces the CCDG size. Cleaning had a moderate impact on the F-score and caused a slight

reduction in the CCDG size. These findings are instrumental in guiding our final decision to use smoothing of k-mer counts as the only preprocessing for input samples.

7.4 Genotyping Workflow

The Great Genotyper implements two genotyping workflows; one for genotyping and imputing phased variants using k-mer counts and phasing information like Pangenie and introduced a novel workflow for unphased variants using k-mer counts only. For the phased variants, the Great Genotyper employs the Pangenie HMM model, which is based on the Li-Stephen model as explained earlier [62]. For the unphased variants, we rely solely on emission probabilities calculated by the model to determine the most probable genotype for each variant. Emission probabilities for the possible alleles are calculated for each sample in the index in parallel. To manage memory efficiently, the emission probabilities for one sub-index are written to disk before processing the next sub-index. Moreover, this step can be scaled up by loading each sub-index on a different node in a distributed system to minimize the running time.

Unlike single-sample genotypers, The Great Genotyper leverages the power of having a large population in the CCDGs to filter low-quality genotypes. This is possible because the genotyping model yields a confidence measure for the output genotypes. The components driving these confidence measures can primarily be distilled into two factors: the number of unique k-mers discovered for each variant haplotype and the count of these k-mers in the sample. The first factor is a constant across all samples since it is determined only from the reference genome and the variant to be genotyped. However, the second factor varies per sample. Some samples may present robust evidence for a particular genotype, while others may not due to either low coverage of the region in the sample or the exhibition of a different haplotype not present in the input haplotypes. Therefore, The Great Genotyper introduces a new quality metric by calculating the median of genotype confidences for each genotype. Thereafter, the genotypes falling below this median are discarded. This approach allows the Great Genotyper to establish a variable threshold calculated using the results from all the samples, providing a balanced way to sift through the variants. For variants abundant in unique k-mers, this threshold will be high, while more challenging variants will have a lower threshold, accommodating the varying levels of confidence in different scenarios. The final output of this step is a reference panel comprised of the high-confidence genotypes. Moreover, The Great Genotyper running on a distributed computational system, has the option to write these confidence probabilities in intermediate files to the disk of each node handling a batch of samples. An aggregation function use these files to run the population-level genotype filtering step.

Finally, Beagle [41, 42] is employed to statistically impute the filtered, low confidence genotypes using this reference panel, simultaneously phasing the resultant variants, thereby yielding phased genotypes for all samples. It is crucial to note that Beagle employs a different HMM model, albeit very similar to the one used in the HMM workflow. In Beagle, linkage disequilibrium is computed statistically from the high-confidence genotypes within the created reference panel. In contrast, the model in the HMM workflow utilizes the phasing information provided by the user in the input variants. The synergy between these two imputation methods does not only enhance the results of genotyping but also broadens the application scope for the higher quality HMM model, enabling its usage when phasing information is absent in the input VCF, as described in the two-pass workflow in Figure 1B.

7.5 Benchmark Experiment Design

This section outlines the experimental design for benchmarking experiments conducted to compare the accuracy of The Great Genotyper with state-of-the-art genotyping tools: Pangenie v3.0.1, GraphTyper v2.72, Paragraph v2.3, and GATK v4.1.3, as described previously [17]. The experiment is structured into two components. The first involves creating benchmarking datasets, including a query variant set and a truth variant set.

To prepare these datasets, variant calling for HG00731 and NA12878 was performed by aligning their haplotype-resolved assemblies against the GRCh38 reference genome using Minimap2 v2.22 [67], followed by variant calling with PAV tools v2.2.6 [68]. To ensure robust benchmarking, only variants within high-confidence regions were selected.

These regions correspond to areas where only one segment of the assemblies maps, excluding segmental duplications and highly repetitive regions, such as centromeres, which are beyond the scope of the evaluated genotypers.

The variants of NA12878 were used to represent the query variant set, while the variants shared between both samples represented the truth variant set. To achieve this, the VCFs of both samples were merged using bcftools v1.16 [69]. Variants unique to HG00731 were filtered out, and the merged VCF was split into two files: the test VCF, where the NA12878 sample column was retained, and the truth VCF, where the HG00731 sample column was retained. Both files contained the same set of variants, differing only in the sample column.

The second component involves running the genotypers and benchmarking their performance. Various genotypers were executed on the test VCF (NA12878) and the SRS derived from the HG00731 sample at different coverages (5x, 10x, 20x, 30x). The genotyping results were compared against the truth VCF using RTG v3.12.1 vcfeval [70] without the –squash-ploidy option. This configuration evaluates each local haplotype separately, enforcing strict genotype comparisons and penalizing mismatched zygosity. A variant was counted as a false positive (FP) if it was called 1/1 while the truth set had it as 0/1. Conversely, it was counted as a false negative (FN) if it was called 0/1 but was 1/1 in the truth set. A variant was counted as a true positive (TP) only if genotypes matched in both VCFs. Precision, recall, and F1-score were calculated based on TP, FP, and FN counts as follows:

$$\text{Precision} = \frac{\text{TPs}}{\text{TPs} + \text{FPs}}.$$

$$\text{Recall} = \frac{\text{TPs}}{\text{TPs} + \text{FNs}}.$$

$$\text{F1-score} = 2 \times \frac{\text{Precision} \times \text{Recall}}{\text{Precision} + \text{Recall}}.$$

The benchmarking results were stratified based on whether the variant was located in a repeat region. Additionally, results were classified by variant type and size: single nucleotide polymorphisms (SNPs), small insertions/deletions (<50 bp), large insertions/deletions ($\geq$ 50 bp), and complex insertions/deletions. Complex variants were defined as those that generate more than one breakpoint.

8 Author Contributions

M.S. and T.M. conceptualized the study, interpreted the results, and wrote the main draft. M.S. was responsible for the implementation of the software. T.M. supervised the work and participated in the data analysis. M.A. contributed to the experiment on reference-free QC. T.B. provided valuable feedback on the study design and reviewed the manuscript.

9 Availability of supporting source code and requirements

Project name: The Great Genotyper
Project home page: `https://github.com/dib-lab/TheGreatGenotyper`
Operating system(s): Linux
Programming language: C++
Other requirements: conda and cmake
License: GNU GPLv3
RRID: NA
bio.tools ID: TheGreatGenotyper

References

[1] Harris A. Lewin, Gene E. Robinson, W. John Kress, William J. Baker, Jonathan Coddington, Keith A. Crandall, Richard Durbin, Scott V. Edwards, Félix Forest, M. Thomas P. Gilbert, Melissa M. Goldstein, Igor V. Grigoriev, Kevin J. Hackett, David Haussler, Erich D. Jarvis, Warren E. Johnson, Aristides Patrinos, Stephen Richards, Juan Carlos Castilla-Rubio, Pamela S. Soltis, Xun Xu, Huanming Yang, and Guojie Zhang. Earth BioGenome Project: Sequencing life for the future of life. 115(17):4325–4333, 2018.

[2] Klaus-Peter Koepfli and Benedict Paten. The Genome 10K Project: A Way Forward. 3(1):57–111.

[3] Cheng Quan, Yuanfeng Li, Xinyi Liu, Yahui Wang, Jie Ping, Yiming Lu, and Gangqiao Zhou. Characterization of structural variation in Tibetans reveals new evidence of high-altitude adaptation and introgression. 22(1):159, 2021.

[4] Kaoru Fujinami, Rupert W. Strauss, John Pei-Wen Chiang, Isabelle S. Audo, Paul S. Bernstein, David G. Birch, Samantha M. Bomotti, Artur V. Cideciyan, Ann-Margret Ervin, Meghan J. Marino, José-Alain Sahel, Saddek Mohand-Said, Janet S. Sunness, Elias I. Traboulsi, Sheila West, Robert Wojciechowski, Eberhart Zrenner, Michel Michaelides, Hendrik P. N. Scholl, ProgStar Study Group, and ProgStar Study Group. Detailed genetic characteristics of an international large cohort of patients with Stargardt disease: ProgStar study report 8. 103(3):390–397.

[5] Hakhamanesh Mostafavi, Tomaz Berisa, Felix R. Day, John R. B. Perry, Molly Przeworski, and Joseph K. Pickrell. Identifying genetic variants that affect viability in large cohorts. 15(9):e2002458, 2017.

[6] Andy W. Pang, Jeffrey R. MacDonald, Dalila Pinto, John Wei, Muhammad A. Rafiq, Donald F. Conrad, Hansoo Park, Matthew E. Hurles, Charles Lee, J. Craig Venter, Ewen F. Kirkness, Samuel Levy, Lars Feuk, and Stephen W. Scherer. Towards a comprehensive structural variation map of an individual human genome. 11(5):R52, 2010.

[7] Colby Chiang, Alexandra J. Scott, Joe R. Davis, Emily K. Tsang, Xin Li, Yungil Kim, Tarik Hadzic, Farhan N. Damani, Liron Ganel, Stephen B. Montgomery, Alexis Battle, Donald F. Conrad, and Ira M. Hall. The impact of structural variation on human gene expression. 49(5):692–699.

[8] Medhat Mahmoud, Nastassia Gobet, Diana Ivette Cruz-Dávalos, Ninon Mounier, Christophe Dessimoz, and Fritz J. Sedlazeck. Structural variant calling: The long and the short of it. 20(1):246, 2019.

[9] Fritz J. Sedlazeck, Philipp Rescheneder, Moritz Smolka, Han Fang, Maria Nattestad, Arndt Von Haeseler, and Michael C. Schatz. Accurate detection of complex structural variations using single-molecule sequencing. 15(6):461–468, 2018.

[10] Kez Cleal and Duncan M Baird. Dysgu: Efficient structural variant calling using short or long reads. 50(9):e53, 2022.

[11] Medhat Mahmoud, Harshavardhan Doddapaneni, Winston Timp, and Fritz J. Sedlazeck. PRINCESS: Comprehensive detection of haplotype resolved SNVs, SVs, and methylation. 22(1):268, 2021.

[12] Haoyu Cheng, Gregory T. Concepcion, Xiaowen Feng, Haowen Zhang, and Heng Li. Haplotype-resolved de novo assembly using phased assembly graphs with hifiasm. 18(2):170–175.

[13] Parsoa Khorsand and Fereydoun Hormozdiari. Nebula: Ultra-efficient mapping-free structural variant genotyper. 49(8):e47, 2021.

[14] Sai Chen, Peter Krusche, Egor Dolzhenko, Rachel M. Sherman, Roman Petrovski, Felix Schlesinger, Melanie Kirsche, David R. Bentley, Michael C. Schatz, Fritz J. Sedlazeck, and Michael A. Eberle. Paragraph: A graph-based structural variant genotyper for short-read sequence data. 20(1), 2019.

[15] Goo Jun, Fritz Sedlazeck, Qihui Zhu, Adam English, Ginger Metcalf, Hyun Min Kang, Human Genome Structural Variation Consortium (HGSVC), Charles Lee, Richard Gibbs, and Eric Boerwinkle. muCNV: Genotyping structural variants for population-level sequencing. 37(14):2055–2057, 2021.

[16] Hannes P. Eggertsson, Snaedis Kristmundsdottir, Doruk Beyter, Hakon Jonsson, Astros Skuladottir, Marteinn T. Hardarson, Daniel F. Gudbjartsson, Kari Stefansson, Bjarni V. Halldorsson, and Pall Melsted. GraphTyper2 enables population-scale genotyping of structural variation using pangenome graphs. 10(1):1–8, 2019.

[17] Jana Ebler, Peter Ebert, Wayne E. Clarke, Tobias Rausch, Peter A. Audano, Torsten Houwaart, Yafei Mao, Jan O. Korbel, Evan E. Eichler, Michael C. Zody, Alexander T. Dilthey, and Tobias Marschall. Pangenome-based genome inference allows efficient and accurate genotyping across a wide spectrum of variant classes. 54(4):518–525.

[18] John Huddleston, Mark J. P. Chaisson, Karyn Meltz Steinberg, Wes Warren, Kendra Hoekzema, David Gordon, Tina A. Graves-Lindsay, Katherine M. Munson, Zev N. Kronenberg, Laura Vives, Paul Peluso, Matthew Boitano, Chen-Shin Chin, Jonas Korlach, Richard K. Wilson, and Evan E. Eichler. Discovery and genotyping of structural variation from long-read haploid genome sequence data. 27(5):677–685, 2017.

[19] Cheng Quan, Hao Lu, Yiming Lu, and Gangqiao Zhou. Population-scale genotyping of structural variation in the era of long-read sequencing. 20:2639–2647, 2022.

[20] Melanie Kirsche, Gautam Prabhu, Rachel Sherman, Bohan Ni, Alexis Battle, Sergey Aganezov, and Michael C. Schatz. Jasmine and Iris: Population-scale structural variant comparison and analysis. 20(3):408–417.

[21] 1000 Genomes Project Consortium, Adam Auton, Lisa D. Brooks, Richard M. Durbin, Erik P. Garrison, Hyun Min Kang, Jan O. Korbel, Jonathan L. Marchini, Shane McCarthy, Gil A. McVean, and Gonçalo R. Abecasis. A global reference for human genetic variation. 526(7571):68–74, 2015.

[22] Jennifer R. S. Meadows, Jeffrey M. Kidd, Guo-Dong Wang, Heidi G. Parker, Peter Z. Schall, Matteo Bianchi, Matthew J. Christmas, Katia Bougiouri, Reuben M. Buckley, Christophe Hitte, Anthony K. Nguyen, Chao Wang, Vidhya Jagannathan, Julia E. Niskanen, Laurent A. F. Frantz, Meharji Arumilli, Sruthi Hundi, Kerstin Lindblad-Toh, Catarina Ginja, Kadek Karang Agustina, Catherine André, Adam R. Boyko, Brian W. Davis, Michaela Drögemüller, Xin-Yao Feng, Konstantinos Gkagkavouzis, Giorgos Iliopoulos, Alexander C. Harris, Marjo K. Hytönen, Daniela C. Kalthoff, Yan-Hu Liu, Petros Lymberakis, Nikolaos Poulakakis, Ana Elisabete Pires, Fernando Racimo, Fabian Ramos-Almodovar, Peter Savolainen, Semina Venetsani, Imke Tammen, Alexandros Triantafyllidis, Bridgett vonHoldt, Robert K. Wayne, Greger Larson, Frank W. Nicholas, Hannes Lohi, Tosso Leeb, Ya-Ping Zhang, and Elaine A. Ostrander. Genome sequencing of 2000 canids by the Dog10K consortium advances the understanding of demography, genome function and architecture. 24(1):187, 2023.

[23] Wen-Wei Liao, Mobin Asri, Jana Ebler, Daniel Doerr, Marina Haukness, Glenn Hickey, Shuangjia Lu, Julian K. Lucas, Jean Monlong, Haley J. Abel, Silvia Buonaiuto, Xian H. Chang, Haoyu Cheng, Justin Chu, Vincenza Colonna, Jordan M. Eizenga, Xiaowen Feng, Christian Fischer, Robert S. Fulton, Shilpa Garg, Cristian Groza, Andrea Guarracino, William T. Harvey, Simon Heumos, Kerstin Howe, Miten Jain, Tsung-Yu Lu, Charles Markello, Fergal J. Martin, Matthew W. Mitchell, Katherine M. Munson, Moses Njagi Mwaniki, Adam M. Novak, Hugh E. Olsen, Trevor Pesout, David Porubsky, Pjotr Prins, Jonas A. Sibbesen, Jouni Sirén, Chad Tomlinson, Flavia Villani, Mitchell R. Vollger, Lucinda L. Antonacci-Fulton, Gunjan Baid, Carl A. Baker, Anastasiya Belyaeva, Konstantinos Billis, Andrew Carroll, Pi-Chuan Chang, Sarah Cody, Daniel E. Cook, Robert M. Cook-Deegan, Omar E. Cornejo, Mark Diekhans, Peter Ebert, Susan Fairley, Olivier Fedrigo, Adam L. Felsenfeld,

Giulio Formenti, Adam Frankish, Yan Gao, Nanibaa' A. Garrison, Carlos Garcia Giron, Richard E. Green, Leanne Haggerty, Kendra Hoekzema, Thibaut Hourlier, Hanlee P. Ji, Eimear E. Kenny, Barbara A. Koenig, Alexey Kolesnikov, Jan O. Korbel, Jennifer Kordosky, Sergey Koren, HoJoon Lee, Alexandra P. Lewis, Hugo Magalhães, Santiago Marco-Sola, Pierre Marijon, Ann McCartney, Jennifer McDaniel, Jacquelyn Mountcastle, Maria Nattestad, Sergey Nurk, Nathan D. Olson, Alice B. Popejoy, Daniela Puiu, Mikko Rautiainen, Allison A. Regier, Arang Rhie, Samuel Sacco, Ashley D. Sanders, Valerie A. Schneider, Baergen I. Schultz, Kishwar Shafin, Michael W. Smith, Heidi J. Sofia, Ahmad N. Abou Tayoun, Françoise Thibaud-Nissen, Francesca Floriana Tricomi, Justin Wagner, Brian Walenz, Jonathan M. D. Wood, Aleksey V. Zimin, Guillaume Bourque, Mark J. P. Chaisson, Paul Flicek, Adam M. Phillippy, Justin M. Zook, Evan E. Eichler, David Haussler, Ting Wang, Erich D. Jarvis, Karen H. Miga, Erik Garrison, Tobias Marschall, Ira M. Hall, Heng Li, and Benedict Paten. A draft human pangenome reference. 617(7960):312–324.

[24] Goo Jun, Adam C. English, Ginger A. Metcalf, Jianzhi Yang, Mark JP Chaisson, Nathan Pankratz, Vipin K. Menon, William J. Salerno, Olga Krasheninina, Albert V. Smith, John A. Lane, Tom Blackwell, Hyun Min Kang, Sejal Salvi, Qingchang Meng, Hua Shen, Divya Pasham, Sravya Bhamidipati, Kavya Kottapalli, Donna K. Arnett, Allison Ashley-Koch, Paul L. Auer, Kathleen M. Beutel, Joshua C. Bis, John Blangero, Donald W. Bowden, Jennifer A. Brody, Brian E. Cade, Yii-Der Ida Chen, Michael H. Cho, Joanne E. Curran, Myriam Fornage, Barry I. Freedman, Tasha Fingerlin, Bruce D. Gelb, Lifang Hou, Yi-Jen Hung, John P. Kane, Robert Kaplan, Wonji Kim, Ruth J. F. Loos, Gregory M. Marcus, Rasika A. Mathias, Stephen T. McGarvey, Courtney Montgomery, Take Naseri, S. Mehdi Nouraie, Michael H. Preuss, Nicholette D. Palmer, Patricia A. Peyser, Laura M. Raffield, Aakrosh Ratan, Susan Redline, Sefuiva Reupena, Jerome I. Rotter, Stephen S. Rich, Michiel Rienstra, Ingo Ruczinski, Vijay G. Sankaran, David A. Schwartz, Christine E. Seidman, Jonathan G. Seidman, Edwin K. Silverman, Jennifer A. Smith, Adrienne Stilp, Kent D. Taylor, Marilyn J. Telen, Scott T. Weiss, L. Keoki Williams, Baojun Wu, Lisa R. Yanek, Yingze Zhang, Jessica Lasky-Su, Marie Claude Gingras, Susan K. Dutcher, Evan E. Eichler, Stacey Gabriel, Soren Germer, Ryan Kim, Karine A. Viaud-Martinez, Deborah A. Nickerson, NHLBI Trans-Omics for Precision Medicine (TOPMed) Consortium, James Luo, Alex Reiner, Richard A. Gibbs, Eric Boerwinkle, Goncalo Abecasis, and Fritz J. Sedlazeck. Structural variation across 138,134 samples in the TOPMed consortium, 2023.

[25] Christian Bauer and Gavin King. *Java Persistence with Hibernate*. Manning Publications Co.

[26] Yang Gao, Xiaofei Yang, Hao Chen, Xinjiang Tan, Zhaoqing Yang, Lian Deng, Baonan Wang, Shuang Kong, Songyang Li, Yuhang Cui, Chang Lei, Yimin Wang, Yuwen Pan, Sen Ma, Hao Sun, Xiaohan Zhao, Yingbing Shi, Ziyi Yang, Dongdong Wu, Shaoyuan Wu, Xingming Zhao, Binyin Shi, Li Jin, Zhibin Hu, Yan Lu, Jiayou Chu, Kai Ye, and Shuhua Xu. A pangenome reference of 36 Chinese populations. 619(7968):112–121.

[27] Xuelei Dai, Peipei Bian, Dexiang Hu, Funong Luo, Yongzhen Huang, Shaohua Jiao, Xihong Wang, Mian Gong, Ran Li, Yudong Cai, Jiayue Wen, Qimeng Yang, Weidong Deng, Hojjat Asadollahpour Nanaei, Yu Wang, Fei Wang, Zijing Zhang, Benjamin D. Rosen, Rasmus Heller, and Yu Jiang. A Chinese indicine pangenome reveals a wealth of novel structural variants introgressed from other Bos species. 33(8):1284–1298, 2023.

[28] Yang Zhou, Lv Yang, Xiaotao Han, Jiazheng Han, Yan Hu, Fan Li, Han Xia, Lingwei Peng, Clarissa Boschiero, Benjamin D. Rosen, Derek M. Bickhart, Shujun Zhang, Aizhen Guo, Curtis P. Van Tassell, Timothy P. L. Smith, Liguo Yang, and George E. Liu. Assembly of a pangenome for global cattle reveals missing sequences and novel structural variations, providing new insights into their diversity and evolutionary history. 32(8):1585–1601, 2022.

[29] Ran Li, Mian Gong, Xinmiao Zhang, Fei Wang, Zhenyu Liu, Lei Zhang, Qimeng Yang, Yuan Xu, Mengsi Xu, Huanhuan Zhang, Yunfeng Zhang, Xuelei Dai, Yuanpeng Gao, Zhuangbiao Zhang, Wenwen Fang, Yuta Yang, Weiwei Fu, Chunna Cao, Peng Yang, Zeinab Amiri Ghanatsaman, Niloufar Jafarpour Negari, Hojjat Asadollahpour Nanaei, Xiangpeng Yue, Yuxuan Song, Xianyong Lan, Weidong Deng, Xihong Wang, Chuanying Pan,

Ruidong Xiang, Eveline M. Ibeagha-Awemu, Pat (J S.) Heslop-Harrison, Benjamin D. Rosen, Johannes A. Lenstra, Shangquan Gan, and Yu Jiang. A sheep pangenome reveals the spectrum of structural variations and their effects on tail phenotypes. 33(3):463–477, 2023.

[30] Yue Huang, Jiaxian He, Yuantao Xu, Weikang Zheng, Shaohua Wang, Peng Chen, Bin Zeng, Shuizhi Yang, Xiaolin Jiang, Zishuang Liu, Lun Wang, Xia Wang, Shengjun Liu, Zhihao Lu, Ziang Liu, Huiwen Yu, Jianqiang Yue, Junyan Gao, Xianyan Zhou, Chunrui Long, Xiuli Zeng, Yong-Jie Guo, Wen-Fu Zhang, Zongzhou Xie, Chunlong Li, Zhaocheng Ma, Wenbiao Jiao, Fei Zhang, Robert M. Larkin, Robert R. Krueger, Malcolm W. Smith, Ray Ming, Xiuxin Deng, and Qiang Xu. Pangenome analysis provides insight into the evolution of the orange subfamily and a key gene for citric acid accumulation in citrus fruits. pages 1–12, 2023.

[31] Ilkka Lappalainen, John Lopez, Lisa Skipper, Timothy Hefferon, J. Dylan Spalding, John Garner, Chao Chen, Michael Maguire, Matt Corbett, George Zhou, Justin Paschall, Victor Ananiev, Paul Flicek, and Deanna M. Church. DbVar and DGVa: Public archives for genomic structural variation. 41(D1), 2013.

[32] Siwei Chen, Laurent C. Francioli, Julia K. Goodrich, Ryan L. Collins, Masahiro Kanai, Qingbo Wang, Jessica Alföldi, Nicholas A. Watts, Christopher Vittal, Laura D. Gauthier, Timothy Poterba, Michael W. Wilson, Yekaterina Tarasova, William Phu, Mary T. Yohannes, Zan Koenig, Yossi Farjoun, Eric Banks, Stacey Donnelly, Stacey Gabriel, Namrata Gupta, Steven Ferriera, Charlotte Tolonen, Sam Novod, Louis Bergelson, David Roazen, Valentin Ruano-Rubio, Miguel Covarrubias, Christopher Llanwarne, Nikelle Petrillo, Gordon Wade, Thibault Jeandet, Ruchi Munshi, Kathleen Tibbetts, gnomAD Project Consortium, Anne O'Donnell-Luria, Matthew Solomonson, Cotton Seed, Alicia R. Martin, Michael E. Talkowski, Heidi L. Rehm, Mark J. Daly, Grace Tiao, Benjamin M. Neale, Daniel G. MacArthur, and Konrad J. Karczewski. A genome-wide mutational constraint map quantified from variation in 76,156 human genomes, 2022.

[33] Daniel Taliun, Daniel N. Harris, Michael D. Kessler, Jedidiah Carlson, Zachary A. Szpiech, Raul Torres, Sarah A. Gagliano Taliun, André Corvelo, Stephanie M. Gogarten, Hyun Min Kang, Achilleas N. Pitsillides, Jonathon LeFaive, Seung-been Lee, Xiaowen Tian, Brian L. Browning, Sayantan Das, Anne-Katrin Emde, Wayne E. Clarke, Douglas P. Loesch, Amol C. Shetty, Thomas W. Blackwell, Albert V. Smith, Quenna Wong, Xiaoming Liu, Matthew P. Conomos, Dean M. Bobo, François Aguet, Christine Albert, Alvaro Alonso, Kristin G. Ardlie, Dan E. Arking, Stella Aslibekyan, Paul L. Auer, John Barnard, R. Graham Barr, Lucas Barwick, Lewis C. Becker, Rebecca L. Beer, Emelia J. Benjamin, Lawrence F. Bielak, John Blangero, Michael Boehnke, Donald W. Bowden, Jennifer A. Brody, Esteban G. Burchard, Brian E. Cade, James F. Casella, Brandon Chalazan, Daniel I. Chasman, Yii-Der Ida Chen, Michael H. Cho, Seung Hoan Choi, Mina K. Chung, Adolfo Correa, Joanne E. Curran, Brian Custer, Dawood Darbar, Michelle Daya, Dawn L. DeMeo, Susan K. Dutcher, Patrick T. Ellinor, Leslie S. Emery, Celeste Eng, Diane Fatkin, Tasha Fingerlin, Lukas Forer, Myriam Fornage, Nora Franceschini, Christian Fuchsberger, Stephanie M. Fullerton, Soren Germer, Mark T. Gladwin, Daniel J. Gottlieb, Xiuqing Guo, Michael E. Hall, Jiang He, Nancy L. Heard-Costa, Susan R. Heckbert, Marguerite R. Irvin, Jill M. Johnsen, Andrew D. Johnson, Robert Kaplan, Sharon L. R. Kardia, Tanika Kelly, Shannon Kelly, Eimear E. Kenny, Douglas P. Kiel, Robert Klemmer, Barbara A. Konkle, Charles Kooperberg, Anna Köttgen, Leslie A. Lange, Jessica Lasky-Su, Daniel Levy, Xihong Lin, Keng-Han Lin, Chunyu Liu, Ruth J. F. Loos, Lori Garman, Robert Gerszten, Steven A. Lubitz, Kathryn L. Lunetta, Angel C. Y. Mak, Ani Manichaikul, Alisa K. Manning, Rasika A. Mathias, David D. McManus, Stephen T. McGarvey, James B. Meigs, Deborah A. Meyers, Julie L. Mikulla, Mollie A. Minear, Braxton D. Mitchell, Sanghamitra Mohanty, May E. Montasser, Courtney Montgomery, Alanna C. Morrison, Joanne M. Murabito, Andrea Natale, Pradeep Natarajan, Sarah C. Nelson, Kari E. North, Jeffrey R. O'Connell, Nicholette D. Palmer, Nathan Pankratz, Gina M. Peloso, Patricia A. Peyser, Jacob Pleiness, Wendy S. Post, Bruce M. Psaty, D. C. Rao, Susan Redline, Alexander P. Reiner, Dan Roden, Jerome I. Rotter, Ingo Ruczinski, Chloé Sarnowski, Sebastian Schoenherr, David A. Schwartz, Jeong-Sun Seo, Sudha Seshadri, Vivien A. Sheehan, Wayne H. Sheu, M. Benjamin Shoemaker, Nicholas L. Smith, Jennifer A.

Smith, Nona Sotoodehnia, Adrienne M. Stilp, Weihong Tang, Kent D. Taylor, Marilyn Telen, Timothy A. Thornton, Russell P. Tracy, David J. Van Den Berg, Ramachandran S. Vasan, Karine A. Viaud-Martinez, Scott Vrieze, Daniel E. Weeks, Bruce S. Weir, Scott T. Weiss, Lu-Chen Weng, Cristen J. Willer, Yingze Zhang, Xutong Zhao, Donna K. Arnett, Allison E. Ashley-Koch, Kathleen C. Barnes, Eric Boerwinkle, Stacey Gabriel, Richard Gibbs, Kenneth M. Rice, Stephen S. Rich, Edwin K. Silverman, Pankaj Qasba, Weiniu Gan, George J. Papanicolaou, Deborah A. Nickerson, Sharon R. Browning, Michael C. Zody, Sebastian Zöllner, James G. Wilson, L. Adrienne Cupples, Cathy C. Laurie, Cashell E. Jaquish, Ryan D. Hernandez, Timothy D. O'Connor, and Gonçalo R. Abecasis. Sequencing of 53,831 diverse genomes from the NHLBI TOPMed Program. 590(7845):290–299.

[34] Melissa J. Landrum, Jennifer M. Lee, George R. Riley, Wonhee Jang, Wendy S. Rubinstein, Deanna M. Church, and Donna R. Maglott. ClinVar: Public archive of relationships among sequence variation and human phenotype. 42:D980–D985, 2014.

[35] Francesco Kumara Mastrorosa, Danny E. Miller, and Evan E. Eichler. Applications of long-read sequencing to Mendelian genetics. 15(1):42, 2023.

[36] Camille Marchet, Christina Boucher, Simon J. Puglisi, Paul Medvedev, Mikaël Salson, and Rayan Chikhi. Data structures based on k-mers for querying large collections of sequencing data sets. 31(1):1–12, 2021.

[37] Rayan Chikhi, Brice Raffestin, Anton Korobeynikov, Robert Edgar, and Artem Babaian. Logan: Planetary-Scale Genome Assembly Surveys Life's Diversity, 2024.

[38] Mikhail Karasikov, Harun Mustafa, Daniel Danciu, Marc Zimmermann, Christopher Barber, Gunnar Rätsch, and André Kahles. MetaGraph: Indexing and Analysing Nucleotide Archives at Petabase-scale, 2020.

[39] Anders Bergström, Shane A. McCarthy, Ruoyun Hui, Mohamed A. Almarri, Qasim Ayub, Petr Danecek, Yuan Chen, Sabine Felkel, Pille Hallast, Jack Kamm, Hélène Blanché, Jean-François Deleuze, Howard Cann, Swapan Mallick, David Reich, Manjinder S. Sandhu, Pontus Skoglund, Aylwyn Scally, Yali Xue, Richard Durbin, and Chris Tyler-Smith. Insights into human genetic variation and population history from 929 diverse genomes. 367(6484):eaay5012, 2020.

[40] Swapan Mallick, Heng Li, Mark Lipson, Iain Mathieson, Melissa Gymrek, Fernando Racimo, Mengyao Zhao, Niru Chennagiri, Susanne Nordenfelt, Arti Tandon, Pontus Skoglund, Iosif Lazaridis, Sriram Sankararaman, Qiaomei Fu, Nadin Rohland, Gabriel Renaud, Yaniv Erlich, Thomas Willems, Carla Gallo, Jeffrey P. Spence, Yun S. Song, Giovanni Poletti, Francois Balloux, Irene Gallego Romero, Aashish R. Jha, Doron M. Behar, Claudio M. Bravi, Cristian Capelli, Tor Hervig, Andres Moreno-Estrada, Olga L. Posukh, Elena Balanovska, Oleg Balanovsky, Sena Karachanak-Yankova, Hovhannes Sahakyan, Draga Toncheva, Levon Yepiskoposyan, Chris Tyler-Smith, Yali Xue, M. Syafiq Abdullah, Andres Ruiz-Linares, Cynthia M. Beall, Anna Di Rienzo, Choongwon Jeong, Elena B. Starikovskaya, Ene Metspalu, Jüri Parik, Richard Villems, Brenna M. Henn, Ugur Hodoglugil, Robert Mahley, Antti Sajantila, George Stamatoyannopoulos, Joseph T. S. Wee, Rita Khusainova, Elza Khusnutdinova, Sergey Litvinov, George Ayodo, David Comas, Michael F. Hammer, Toomas Kivisild, William Klitz, Cheryl A. Winkler, Damian Labuda, Michael Bamshad, Lynn B. Jorde, Sarah A. Tishkoff, W. Scott Watkins, Mait Metspalu, Stanislav Dryomov, Rem Sukernik, Lalji Singh, Kumarasamy Thangaraj, Svante Pääbo, Janet Kelso, Nick Patterson, and David Reich. The Simons Genome Diversity Project: 300 genomes from 142 diverse populations. 538(7624):201–206.

[41] Brian L. Browning, Ying Zhou, and Sharon R. Browning. A One-Penny Imputed Genome from Next-Generation Reference Panels. 103(3):338–348, 2018.

[42] Brian L. Browning, Xiaowen Tian, Ying Zhou, and Sharon R. Browning. Fast two-stage phasing of large-scale sequence data. 108(10):1880–1890, 2021.

[43] Qingbo S. Wang and Hailiang Huang. Methods for statistical fine-mapping and their applications to autoimmune diseases. 44(1):101–113.

[44] Marta Byrska-Bishop, Uday S. Evani, Xuefang Zhao, Anna O. Basile, Haley J. Abel, Allison A. Regier, André Corvelo, Wayne E. Clarke, Rajeeva Musunuri, Kshithija Nagulapalli, Susan Fairley, Alexi Runnels, Lara Winterkorn, Ernesto Lowy, Evan E. Eichler, Jan O. Korbel, Charles Lee, Tobias Marschall, Scott E. Devine, William T. Harvey, Weichen Zhou, Ryan E. Mills, Tobias Rausch, Sushant Kumar, Can Alkan, Fereydoun Hormozdiari, Zechen Chong, Yu Chen, Xiaofei Yang, Jiadong Lin, Mark B. Gerstein, Ye Kai, Qihui Zhu, Feyza Yilmaz, Chunlin Xiao, Paul Flicek, Soren Germer, Harrison Brand, Ira M. Hall, Michael E. Talkowski, Giuseppe Narzisi, and Michael C. Zody. High-coverage whole-genome sequencing of the expanded 1000 Genomes Project cohort including 602 trios. 185(18):3426–3440.e19, 2022.

[45] Justin M. Zook, Nancy F. Hansen, Nathan D. Olson, Lesley Chapman, James C. Mullikin, Chunlin Xiao, Stephen Sherry, Sergey Koren, Adam M. Phillippy, Paul C. Boutros, Sayed Mohammad E. Sahraeian, Vincent Huang, Alexandre Rouette, Noah Alexander, Christopher E. Mason, Iman Hajirasouliha, Camir Ricketts, Joyce Lee, Rick Tearle, Ian T. Fiddes, Alvaro Martinez Barrio, Jeremiah Wala, Andrew Carroll, Noushin Ghaffari, Oscar L. Rodriguez, Ali Bashir, Shaun Jackman, John J. Farrell, Aaron M. Wenger, Can Alkan, Arda Soylev, Michael C. Schatz, Shilpa Garg, George Church, Tobias Marschall, Ken Chen, Xian Fan, Adam C. English, Jeffrey A. Rosenfeld, Weichen Zhou, Ryan E. Mills, Jay M. Sage, Jennifer R. Davis, Michael D. Kaiser, John S. Oliver, Anthony P. Catalano, Mark J. P. Chaisson, Noah Spies, Fritz J. Sedlazeck, and Marc Salit. A robust benchmark for detection of germline large deletions and insertions. 38(11):1347–1355.

[46] Peter Krusche, Len Trigg, Paul C. Boutros, Christopher E. Mason, Francisco M. De La Vega, Benjamin L. Moore, Mar Gonzalez-Porta, Michael A. Eberle, Zivana Tezak, Samir Lababidi, Rebecca Truty, George Asimenos, Birgit Funke, Mark Fleharty, Brad A. Chapman, Marc Salit, and Justin M. Zook. Best practices for benchmarking germline small-variant calls in human genomes. 37(5):555–560.

[47] Adam C. English, Vipin K. Menon, Richard Gibbs, Ginger A. Metcalf, and Fritz J. Sedlazeck. Truvari: Refined Structural Variant Comparison Preserves Allelic Diversity, 2022.

[48] Véronique Geoffroy, Yvan Herenger, Arnaud Kress, Corinne Stoetzel, Amélie Piton, Hélène Dollfus, and Jean Muller. AnnotSV: An integrated tool for structural variations annotation. 34(20):3572–3574, 2018.

[49] J. A. Kant, A. J. Fornace, D. Saxe, M. I. Simon, O. W. McBride, and G. R. Crabtree. Evolution and organization of the fibrinogen locus on chromosome 4: Gene duplication accompanied by transposition and inversion. 82(8):2344–2348.

[50] A. M. Carter, A. J. Catto, H. P. Kohler, R. A. Ariëns, M. H. Stickland, and P. J. Grant. Alpha-fibrinogen Thr312Ala polymorphism and venous thromboembolism. 96(3):1177–1179, 2000.

[51] Yu-Lin Ko, Lung-An Hsu, Tsu-Shiu Hsu, Chia-Ti Tsai, Ming-Sheng Teng, Semon Wu, Chi-Jen Chang, and Ying-Shiung Lee. Functional polymorphisms of FGA, encoding alpha fibrinogen, are associated with susceptibility to venous thromboembolism in a Taiwanese population. 119(1-2):84–91.

[52] Laura J. Rasmussen-Torvik, Mary Cushman, Michael Y. Tsai, Yan Zhang, Susan R. Heckbert, Wayne D. Rosamond, and Aaron R. Folsom. The association of alpha-fibrinogen Thr312Ala polymorphism and venous thromboembolism in the LITE study. 121(1):1–7.

[53] Grégoire Le Gal, Bénédicte Delahousse, Karine Lacut, Vincent Malaviolle, Sandra Regina, Marie-Thérèse Blouch, Francis Couturaud, Dominique Mottier, Emmanuel Oger, Yves Gruel, and Groupe d'Etudes sur la Thrombose des Hôpitaux Universitaires du Grand Ouest. Fibrinogen Aalpha-Thr312Ala and factor XIII-A Val34Leu polymorphisms in idiopathic venous thromboembolism. 121(3):333–338.

[54] Zhiyong Chen, Toshiaki Nakajima, Nobuhiro Tanabe, Kunihiko Hinohara, Seiichiro Sakao, Yasunori Kasahara, Koichiro Tatsumi, Yoshinori Inoue, and Akinori Kimura. Susceptibility to chronic thromboembolic pulmonary hypertension may be conferred by miR-759 via its targeted interaction with polymorphic fibrinogen alpha gene. 128(4):443–452.

[55] J. A. Remijn, prefix=de useprefix=true family=Groot, given=P. G., and prefix=van useprefix=true family=Solinge, given=W. W. Nature of the fibrinogen Aalpha gene TaqI polymorphism. 86(3):935–936.

[56] Rehana S. Lovely, Qiong Yang, Joseph M. Massaro, Jing Wang, Ralph B. D'Agostino, Christopher J. O'Donnell, Jackilen Shannon, and David H. Farrell. Assessment of genetic determinants of the association of γ' fibrinogen in relation to cardiovascular disease. 31(10):2345–2352.

[57] Ivan Pokrovac and Zeljka Pezer. Recent advances and current challenges in population genomics of structural variation in animals and plants. 13.

[58] Rasko Leinonen, Hideaki Sugawara, and Martin Shumway. The Sequence Read Archive. 39:D19–D21.

[59] Kiran V. Garimella, Zamin Iqbal, Michael A. Krause, Susana Campino, Mihir Kekre, Eleanor Drury, Dominic Kwiatkowski, Juliana M. Sá, Thomas E. Wellems, and Gil McVean. Detection of simple and complex de novo mutations with multiple reference sequences. 30(8):1154–1169, 2020.

[60] C. Titus Brown and Luiz Irber. Sourmash: A library for MinHash sketching of DNA. 1(5):27, 2016.

[61] Mohamed Abuelanin and Tamer Mansour. Snipe, 2024.

[62] Na Li and Matthew Stephens. Modeling Linkage Disequilibrium and Identifying Recombination Hotspots Using Single-Nucleotide Polymorphism Data. 165(4):2213–2233, 2003.

[63] kSpider.

[64] Marek Kokot, Maciej Dlugosz, and Sebastian Deorowicz. KMC 3: Counting and manipulating k-mer statistics. 33(17):2759–2761, 2017.

[65] SciPy documentation — SciPy v1.11.3 Manual.

[66] Ivica Letunic and Peer Bork. Interactive Tree Of Life (iTOL) v5: An online tool for phylogenetic tree display and annotation. 49(W1):W293–W296, 2021.

[67] Heng Li. Minimap2: Pairwise alignment for nucleotide sequences. 34(18):3094–3100, 2018.

[68] Peter Ebert, Peter A. Audano, Qihui Zhu, Bernardo Rodriguez-Martin, David Porubsky, Marc Jan Bonder, Arvis Sulovari, Jana Ebler, Weichen Zhou, Rebecca Serra Mari, Feyza Yilmaz, Xuefang Zhao, PingHsun Hsieh, Joyce Lee, Sushant Kumar, Jiadong Lin, Tobias Rausch, Yu Chen, Jingwen Ren, Martin Santamarina, Wolfram Höps, Hufsah Ashraf, Nelson T. Chuang, Xiaofei Yang, Katherine M. Munson, Alexandra P. Lewis, Susan Fairley, Luke J. Tallon, Wayne E. Clarke, Anna O. Basile, Marta Byrska-Bishop, André Corvelo, Uday S. Evani, Tsung-Yu Lu, Mark J. P. Chaisson, Junjie Chen, Chong Li, Harrison Brand, Aaron M. Wenger, Maryam Ghareghani, William T. Harvey, Benjamin Raeder, Patrick Hasenfeld, Allison A. Regier, Haley J. Abel, Ira M. Hall, Paul Flicek, Oliver Stegle, Mark B. Gerstein, Jose M. C. Tubio, Zepeng Mu, Yang I. Li, Xinghua Shi, Alex R. Hastie, Kai Ye, Zechen Chong, Ashley D. Sanders, Michael C. Zody, Michael E. Talkowski, Ryan E. Mills, Scott E. Devine, Charles Lee, Jan O. Korbel, Tobias Marschall, and Evan E. Eichler. Haplotype-resolved diverse human genomes and integrated analysis of structural variation. 372(6537):eabf7117, 2021.

[69] Petr Danecek, James K Bonfield, Jennifer Liddle, John Marshall, Valeriu Ohan, Martin O Pollard, Andrew Whitwham, Thomas Keane, Shane A McCarthy, Robert M Davies, and Heng Li. Twelve years of SAMtools and BCFtools. 10(2):giab008, 2021.

[70] John G. Cleary, Ross Braithwaite, Kurt Gaastra, Brian S. Hilbush, Stuart Inglis, Sean A. Irvine, Alan Jackson, Richard Littin, Sahar Nohzadeh-Malakshah, Mehul Rathod, David Ware, Len Trigg, and Francisco M. De La Vega. Joint variant and de novo mutation identification on pedigrees from high-throughput sequencing data. 21(6):405–419, 2014.

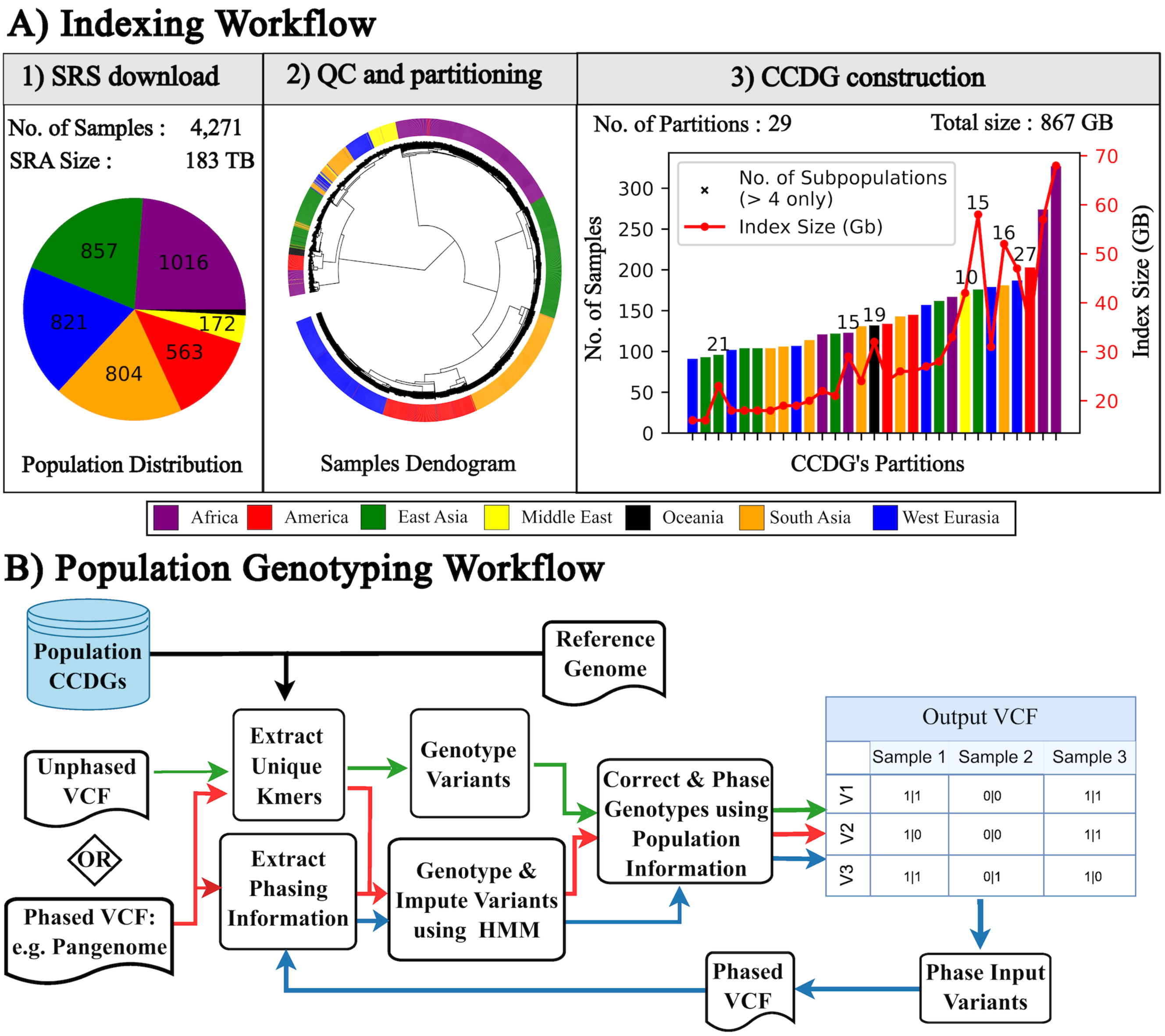

Figure 1: **The Great Genotyper workflows** The indexing workflow (A) depicts the high-level pipeline for creating Population CCDGs. The workflow downloads and computes the unitigs of each sample individually (A1). A sourmash signature is calculated for each sample to be used for alignment-free quality control and sample partitioning (A2). Lastly, a subgraph is created for each partition of samples (A3). The genotyping workflow (B) describes three population genotyping workflows illustrated with a different color of arrows: The HMM workflow (red) genotypes and imputes phased variants using a high-quality HMM model, the k-mer-based workflow (green) rapidly genotypes unphased variants, and the two-pass workflow (blue) enhances the recall of the k-mer-based workflow by genotyping its output phased variants using the HMM workflow.

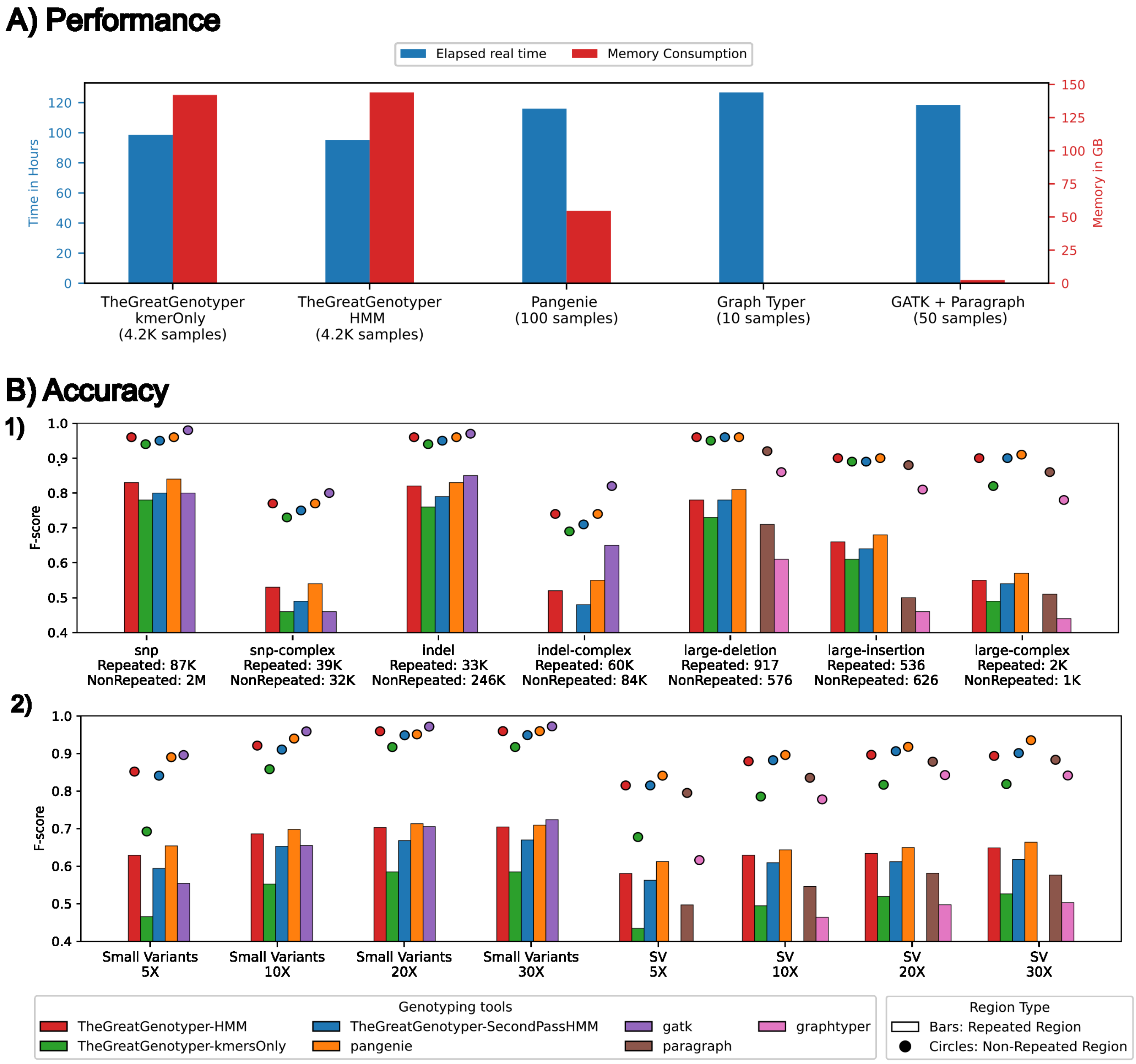

Figure 2: **The Great Genotyper provides unparalleled performance compared to the state of the art, with no compromise on accuracy. Panel A** shows the running time and memory usage of different tools used to genotype 4.5 million phased variants (including structural variants and small variants). The Great Genotyper is currently genotyping 4,200 samples at 30x coverage assuming the indexing of the samples was already built, while the other genotypers are handling 10 to 100 samples. **Panel B1** illustrates the F-scores of different genotyping methods for different classes of variants. **Panel B2** illustrates the effect of coverage on the F-scores of different genotyping methods for small and structural variants. In both Panels B1 and B2, the variants are categorized based on the complexity of the genomic loci into variants located in repeated (shown as bars) and non-repeated regions (shown as circles)

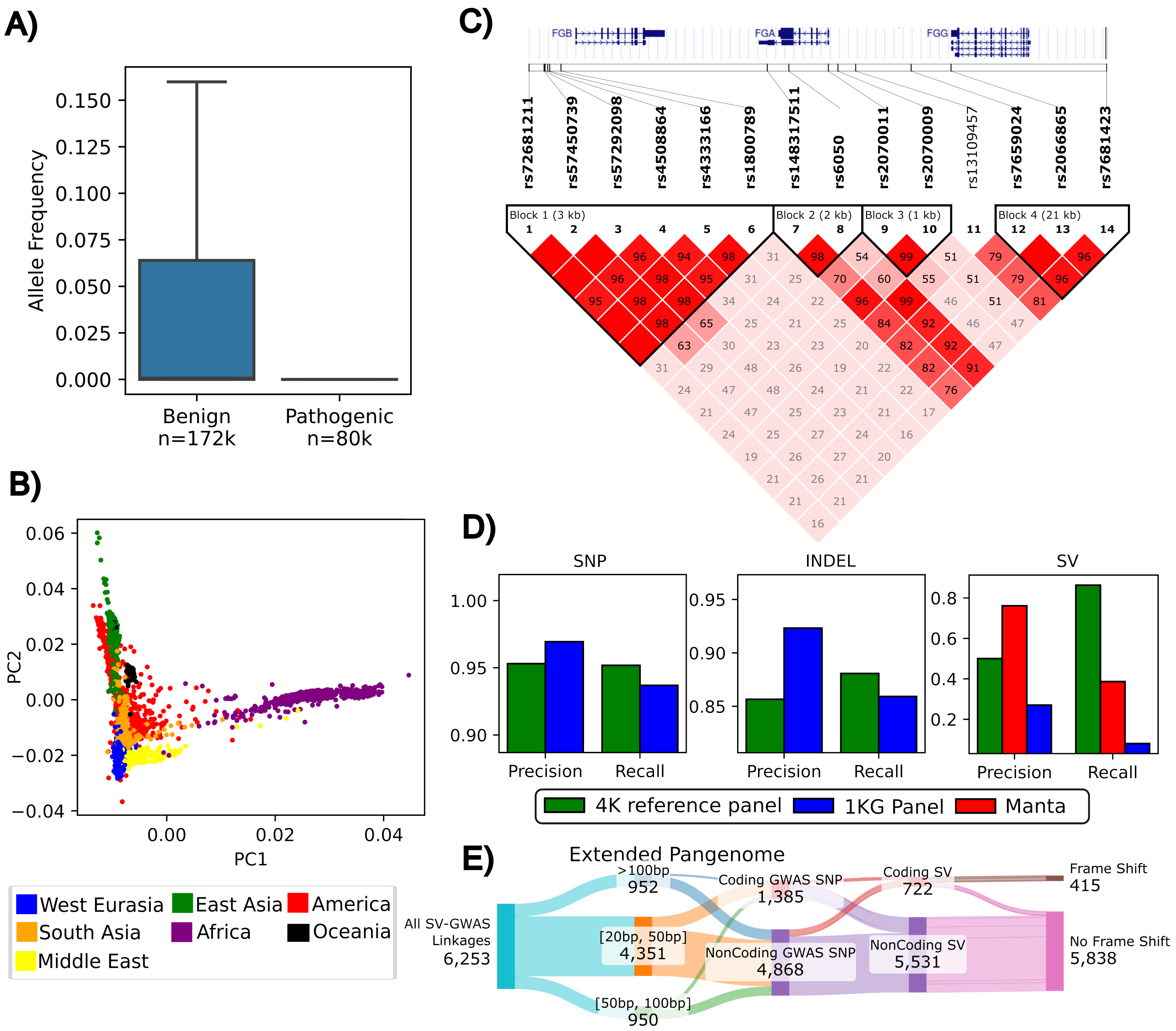

Figure 3: **Applications of The Great Genotyper:** We used the Great Genotyper to genotype all ClinVar and HPRC pangenome variants in 4k human samples. **Panel A** is a box plot of the distinctive distributions of population allele frequencies for ClinVar variants when stratified by the pathogenicity of the variants (outliers are not displayed). **Panel B** is a plot of the first two principal components from a PCA for the genotypes of the HPRC pangenome variants; the 4k samples are colored by their ancestry. **Panel C** is an LD heatmap that highlights the associations of an insertion (dbSNP: rs148317511) and multiple GWAS SNPs including rs6050-C; a peak associating SNP in a GWAS study of the circulating fibrinogen. **Panel D** shows the precision and recall of variant imputation using the 4k reference panel in comparison to variant imputation using the 1000 Genome panel and SV calling using Manta. **Panel E** presents a Sankey plot summarizing 6.2K linkage associations between SVs from the HPRC pangenome and the GWAS catalog. The columns stratify linkages based on various traits of both SVs and GWAS SNPs: SV size, GWAS SNP impact on coding regions, SV impact on coding regions, and SV-induced frameshifts.

Supplementary Table 3

Click here to access/download

Supplementary Material

Supplementary table 3.xlsx

Click here to access/download
Supplementary Material
Supplementary.docx
