# Supplementary material for: The Great Genotyper: a graph-based method for population genotyping of small and structural variants
Source: Gigascience. 2025 Oct 3;14:giaf112. doi: 10.1093/gigascience/giaf112 (PMC12491952; doi:10.1093/gigascience/giaf112)
Supplement: giaf112_Authors_Response_To_Reviewer_Comments_original_submission [file giaf112_authors_response_to_reviewer_comments_original_submission.pdf]

Author's Response To Reviewer Comments

Dear Reviewers,
We would like to sincerely thank you for your thoughtful and constructive feedback on our manuscript. Your questions and comments have been invaluable in helping us improve the quality and clarity of our work. We appreciate the time and effort you invested in reviewing our submission, and we have carefully addressed each of your suggestions in the revised manuscript. Please see below for our responses to each comment.

Reviewer #1:

Comment1:
I would like to see a more explicit summary of the innovations introduced by the authors, and a more clear positioning of this work and MetaGraph (see below for more on this).

Response 1:
We appreciate the reviewer's suggestion of explicitly summarizing the innovation in the current manuscript and positioning the tool among the foundational software used to build it. Therefore, we addressed this issue in multiple sections of the manuscript conceptually and technically.

A) conceptually:
1. The first two paragraphs of the discussion now explain how The Great Genotyper solves the N+1 problem of genotyping and how it compares to the current state of art genotypers.

2. In addition, the last paragraph of the introduction was transformed into three new paragraphs. The first one highlights the role of Metgraph as a foundational concept for our software. The next paragraph summarizes our pipeline and highlights the novelties. The last paragraph describes the human population genotyping as a proof of concept.

3. Also, we updated the 1st paragraph of the results (Section 3.1) to make it clear that our most important novelty is providing a solution for the N+1 problem of genotyping a new set of variants in a big population cohort.

B) Technically
A whole new section was added at the beginning of the Methods (section 7.1). This new section allows clear positioning of our software among the key software packages that we used to implement the indexing and genotyping workflows. Then we have a section on all technical innovations in the Great Genotyper

Comment 2:
Starting with the abstract, the "Counting Colored De Bruijn Graph" (CCDG) data structure is mentioned several places in the manuscript without detailed explanation. In particular, just reading the Methods section alone does not make it completely clear
that CCDG here refers to the data structure employed by Metagraph [M Karasikov et al. 2020].
In Results section 3.1, the authors do clearly state that Metagraph is used to construct CCDGs, but I would expect to find that information in the Methods section. Moreover it seems that perhaps Metagraph is rather central to this work(?)
If so, then it should be given more prominence in the manuscript.

Response 2:
Thank you for the thoughtful feedback. We have considered your comments and made edits to address your concerns:
In the Introduction, we updated the closing section to provide a detailed explanation of the concept of indexing. This includes clarifying the general principles of indexing, with specific focus on its application in

solving the N+1 problem (lines 83-92). Additionally, we introduced Metagraph and highlighted its foundational role in our approach, ensuring that readers can appreciate its significance from the outset.
In the Methods Section (7.1), we added a new subsection to explicitly detail all the tools employed in the study. This includes a comprehensive description of Metagraph and the Counting Colored De Bruijn Graphs (CCDGs). Furthermore, this section outlines a technical gap analysis, which identifies limitations in existing methodologies, and provides an overview of the genotyper design, showcasing the innovative aspects of our methodology.

Comment 3:
Incidentally, "sourmash sketch" is another non-self-explanatory term which comes up in multiple spots in the manuscript and is partially explained in section 3.1 as a "FracMinHash"
calculated by sourmash [cite] used "for quality control including calculation of sequencing depth, ...". Other than this description belonging under methods, it is useful as far as it goes, and yes the reader can refer to the citation for a detailed description. However as a reader I would have appreciated a line or two more giving a rough explanation of what a sourmash sketch or FracMinHash actually is.

Response 3:
Thank you for pointing out that Sourmash was not adequately explained, which could hinder the understanding of the manuscript. To address this, we have added subsection 7.1.4 to provide a detailed explanation of Sourmash and the concept of FracMinHash.

Comment 4:
In general, much of the content of section 3.1 seems to be more suitably placed under Methods rather than Results.

Response 4:
Thank you for your suggestion. We agree that the content of section 3.1 was too technical therefore we did the following:

1) A whole new section was added at the beginning of the Methods (section 7.1) with an overview of the Great Genotyper design and a detailed description of the different software packages we use in our workflows.

2) We updated Section 3.1 to remove technical details. However, in our manuscript, as a Technical Note, the software itself is a product that we need to present in the Results. Therefore, we present the tool conceptually in 3.1 while presenting the deeper technical details in the Methods

Comment 5:
IMPORTANT BUT EASILY FIXED
- In the References section, all citations seem to be missing the year field.
- "Figure 3E summarizes of the associations" --> "Figure 3E summarizes the associations"
- Misspellings in Suppl. Fig. 3 legend

Response 5:
Thank you for the careful review. We updated the text as advised.

Comment 6:
The text does not directly refer to subfigure 3D. I think it depicts results described in Section 3.3.3 Impute SV by using the 4k reference panel. I would be more sure if the main text referred to that subfigure at the appropriate spot.

Response 6:
The citation of subfigure 3D is now in line 224

Comment 7:
When reading the legend of Figure 3E, I did not know what a "Sankey plot" was; and whether is it a general category of plot or something specific to genomics. So an explanation or reference might be in order. On the other hand; Wikipedia does have entries for "Sankey plot" and "alluvial diagram", so I would leave this suggestion to the discretion of the authors.

Response 7:
Thank you for your feedback. We agree that both the "Sankey plot" and the "alluvial diagram" can be used to describe our results here. We were inclined toward using a Sankey plot over an alluvial diagram because it better emphasizes the concept of direction, which is more suitable to our case where the illustrated categories are better seen from left to right.

To assist readers unfamiliar with Sankey diagrams, we have added more explanation to the result section about what the Sankey plot is.

Reviewer #2:

Comment 1:
1) The genotyping workflow is designed specifically for cases where the set of SRS samples is fixed. While there might be applications for which this is the case, I think the much more frequent scenario is that the set of SRS samples is variable, as new SRS datasets are being produced continuously. Is it possible to efficiently update the CCDG in such cases, i.e. can new SRS datasets be efficiently added to an existing CCDG, or does the whole index need to be rebuilt in such cases? Furthermore, I think many real-world scenarios require building a CCDG from scratch, e.g. if one wants to genotype a special cohort of samples, samples sequenced with a specific coverage, newly sequenced samples, etc. In practice, I'm not sure if there are many cases that really benefit from the strength of the method (being able to reuse a precomputed index for a specific population). Therefore, I think it would be good to highlight such use cases more and give concrete examples to convince the
reader why it is useful.

Response 1:
- We agree with the reviewer that building a CCDG from scratch is a common scenario. It seems that our initial text did not clarify that enough. We are presenting the 4.2k human samples index as a valuable resource that can be used for genotyping. However, this index is only a use case that can be replicated with any new cohort. We indicated this now in multiple locations. Here are some pointers:
1. The last paragraph of the Introduction clearly indicates now that this human index is just a use case.
2. Discussion section (lines 323-328) suggests that creating more indexes for other organisms and human subpopulations is doable

Also, we added a link in the code availability section to direct the users to the appropriate github base to do the indexing on any cohort of samples.

In addition, we added more benchmark work on the scalability of indexing to guide the user in choosing the right parameter for their dataset.

- Regarding adding more samples to an already existing index is another important application. The distributed design of our index allows adding any new samples as an additional sub-index. We highlighted this scenario in multiple locations e.g. in the second paragraph of the discussion section(lines 281-288). Also, in section 3.1 (3rd paragraph: lines 117-120).

- Our section of use cases highlights the applications of a pre-computed index. For example, (Section 3.3.1) any genetic study trying to uncover the causative variants of a disease would typically use the population allele frequency of the novel variants detected in the cases to exclude the likely non-pathogenic variants. The Great Genotyper with the pre-computed index provides a first-of-kind resource to calculate the population allele frequency of SVs. In addition, as in section 3.3.2, with more haploid assemblies, The Great Genotyper provides an easy way to incorporate the variants of these assemblies in a big reference panel with a more accurate imputation power.

In the introduction, we motivated the idea of population genotyping by citing previous research(Huddleston et al) where true SV (confirmed by long-read data) is missed by short-read SV callers but captured by genotypers.

Comment 2:
2) In line 133 the authors state: "The Great Genotyper could genotype 4.5 million variants across 4.2K WGS samples in approximately 100 hours, utilizing 32 cores and 145 GB of memory, as depicted in Figure 2A. To put this performance into context, Pangenie and GraphTyper2 required nearly an hour and 12 hours, respectively, to genotype the same 4.5 million variants in a single sample using the same machine." From what I understand, these time / memory requirements do not include the time needed to construct the CCDG index structure. I think all three genotyping workflows should be compared based on their runtime and memory usages starting from the raw reads, because this is what matters in practice. Currently, the resources needed to build the CCDG are not included. The authors must add the runtime and memory requirements of the indexing step on a single 32 core machine with 145 GB of RAM (as used for the other two tools) because this step has to be done, even if it is just
once, so it should not be neglected.

Response 2:
We agree with the reviewer's judgment. The real advantage of our software is the genotyping of the same cohort of new sets of variants. Therefore, we applied several changes to make this clear:

1. As advised by the reviewer, a new paragraph describing the time and memory requirements to construct the CCDG index from raw data for the 4.2K WGS was added to the Results (Lines: 130-133).

2. We also updated the paragraph mentioned by the reviewer (now lines: 156-162) to make it clear that computational resources of indexing should be considered in the first run.

3. Finally, we updated the first paragraph in the discussion (Line 277-280) to make our message clear. This sentence says “With this design, genotyping a large population once for a given set of variants using The Great Genotyper is still computationally on par with leading state-of-the-art genotyping tools. However, thanks to its pre-built index, re-genotyping the same cohort for any new set of variants becomes a much easier computational task.”

Comment 3:
3) In addition to adding detailed time and memory usage of the indexing workflow (see comment above), how does the construction of the index and the genotyping scale in terms of runtime and memory usage for larger sets of SRS samples? And how does the size of the index scale? Would the workflow be applicable to large collections of SRS data, e.g. the UK biobank? What is the limit in terms of sample size?

Response 3:
We would like to thank the reviewer for highlighting the importance of a comprehensive analysis of scalability, which is crucial to estimate the requirements required by The Great Genotyper for different datasets.

The Great Genotyper is designed to be scalable for large populations by the distributed structure of its

index where the index is composed of multiple sub-indexes that each can be constructed and genotyped independently (i.e. map and reduce design) then results can be merged in one final step. The users can design these sub-indexes to match their computational resources. The larger the sub-index, the more memory is needed for both construction and genotyping with a trade in the time needed to genotype the whole population. We originally presented the 4K index composed of 29 sub-indexes with variable sizes to show the recommended range of samples that can be indexed in one partition. The size of the sub-indexes relative to the number of samples was presented in Figure 1, Panel A3. This concept of distributed structure and its importance in scalability was not clear enough. Therefore, we further highlighted this concept in the methods (Lines:411-416, 464-466, and 480-483, results (Lines:105-108), and discussion (Lines: 281-283) sections
In addition, and to make it more clear, we conducted multiple new experiments to test the scalability analysis of both the indexing and genotyping workflows and generated supplementary figures 8-11 to show these results. We summarized these results in a new paragraph in the Results section (lines 109–122). Users can extrapolate these results to any population size. Here is a brief description of these experiments:

a) The indexing workflow is divided into two components: preprocessing samples independently followed by creating sub-indexes, each including up to a few hundred of samples. Therefore, we tested the time needed for preprocessing time for 1,000 human samples of variable sizes to calculate an average for a sample with 30x coverage (Supplementary Figure 8). We also conducted an experiment to assess the scalability of creating sub-indexes from preprocessed samples. The results are shown in Supplementary Figure 9. Sub-indexes were generated with varying numbers of samples to help researchers determine the optimal number of samples for their use case.
b) The genotyping workflow includes two main components: genotyping with the Great Genotyper and imputation/phasing with Beagle. Supplementary Figure 10 demonstrates that genotyping time and memory requirements are primarily influenced by the number of variants, while the number of samples in the sub-indexes has less impact. For imputation, Supplementary Figure 11 shows a similar trend: processing time increases exponentially with the number of variants but grows linearly with the number of samples.

Comment 4:
4) I think the benchmarking experiments were well designed, but some more details should be provided. How are precision/recall/F-score metrics computed exactly? Do these metrics evaluate the actual genotypes or just presence/absence signals? i.e. if a variant is genotyped as 0/1 while being homozygous (1/1) in the ground truth, would this error be counted? The versions of different tools used in the study should also be mentioned. Was the latest version of each genotyping tool used for the benchmarking experiments?

Response 4:
The reviewer highlighted an important issue in benchmarking VCF files. Therefore, we restructured the benchmarking section to show a detailed description of the benchmarking process and how truth and query variant sets were created. In addition, we explained our configuration of the RTG vcfeval tool and how this configuration affects the matching between homozygous and heterozygous calls. In brief, these calls will be considered as a mismatch. It is counted as a false positive if the truth set is 0/1 and counted as a false negative if the truth set is 1/1 and added definitions of precision/recall/F-score metrics

We also added the version numbers to all software packages used in benchmarking

Comment 5:
5) In line 197 it is stated that no similar reference panel is available for SVs. There is in fact a more recent, improved reference panel available for the 1kg cohort which does include SVs as well, see: Byrska-Bishop et al, PMID: 36055201. How do the results compare to this panel?

Response 5:
We thank the reviewers for their suggestions. We used this panel for SNPs and INDELS but mistakenly

cited an older 1K Genome paper. Now, we fixed the citation and used it for benchmarking SVs as well. We evaluated the precision and recall for the 1K Genome panel and found that both metrics lag behind those of our 4K reference panel, as shown in the updated Figure 3D.
We investigated the reasons behind the superior performance of the new panel and identified two key factors. First, the number of structural variants discovered in the pangenomes is significantly greater than in the 1K Genome panel. Second, the number of alleles per site is much larger in the pangenome. These findings have been integrated into lines 230–239 and are further supported by Supplementary Figure 12.

Comment 6:
6) The description of how the genotyping algorithm works needs to be more elaborate, i.e. which/how components are used from other tools like PanGenie and Beagle, and which parts have been newly developed. How exactly do the steps work? I think the Methods section as it is right now is not detailed enough for the reader to understand how the method works. Also, more details on the evaluation metrics should be given (see my comment further above).

Response 6:
Thank you for your feedback. We have restructured the Methods section and added more details to improve clarity. Section 7.1 has been added to begin with a description of the available tools, providing users with an overview of what is already implemented. Subsection 7.1.5 explains the design of the Great Genotyper in greater detail, elaborating on the gap analysis and highlighting the contributions of this work, including the development of new C++-based tools and Snakemake workflows.
Section 7.4 has been expanded to provide a more comprehensive explanation of the genotyping workflow, with a focus on the interaction between its various components.
Additionally, Section 7.5 has been rewritten to clarify the benchmarking workflow. We have added more information about the evaluation metrics, the tools used for evaluation, and the versions of all tools.

Comment 7:
7) Is the pipeline to generate the index CCDG publicly available? The code provided here https://github.com/dib-lab/TheGreatGenotyper/tree/master seems to only contain the genotyper. Furthermore, documentation and instructions on how to construct indexes need to be provided.

Response 7:
Thank you for pointing out that the indexing workflow folder was not obvious. We already had a Snakemake workflow for indexing and a wiki explaining how to use it (https://github.com/dib-lab/TheGreatGenotyper/tree/master/DatabaseBuilder). We also updated the Data and Code Availability section to include direct links to the indexing workflow, making it more accessible to interested readers.

Comment 8:
Minor Comments:
1) Line 180: the HPRC citation is missing
2) Line 345: Pangenine -> Pangenie (typo)

Response 8:
Text was updated as advised

Thank you once again for your dedication to improving the quality of scientific research.

Cookie Preference Center

We use cookies which are necessary to make our site work. We may also use additional cookies to analyze, improve and personalize our content and your digital experience. For more information, see our Cookie Policy.

You may choose not to allow some types of cookies. However, blocking some types may impact your experience of our site and the services we are able to offer. See the different category headings below to find out more or change your settings.

Allow all

Manage Consent Preferences

Strictly Necessary Cookies

Always active

These cookies are necessary for the website to function and cannot be switched off in our systems. They are usually only set in response to actions made by you which amount to a request for services, such as setting your privacy preferences, logging in or filling in forms. You can set your browser to block or alert you about these cookies, but some parts of the site will not then work.

Functional Cookies

These cookies enable the website to provide enhanced functionality and personalisation. They may be set by us or by third party providers whose services we have added to our pages. If you do not allow these cookies then some or all of these services may not function properly.

Performance Cookies

These cookies allow us to count visits and traffic sources so we can measure and improve the performance of our site. They help us to know which pages are the most and least popular and see how visitors move around the site.

Targeting Cookies

These cookies may be set through our site by our advertising partners. They may be used by those companies to build a profile of your interests and show you relevant adverts on other sites. If you do not allow these cookies, you will experience less targeted advertising.

Cookie List

Clear

Apply Cancel

Consent Leg.Interest

Confirm my choices

Powered by onetrust
