# Supplementary material for: The Great Genotyper: a graph-based method for population genotyping of small and structural variants
Source: Gigascience. 2025 Oct 3;14:giaf112. doi: 10.1093/gigascience/giaf112 (PMC12491952; doi:10.1093/gigascience/giaf112)
Supplement: giaf112_Authors_Response_To_Reviewer_Comments_Revision_1 [file giaf112_authors_response_to_reviewer_comments_revision_1.pdf]

Author's Response To Reviewer Comments

Dear Reviewer,

We would like to thank you for your constructive feedback on our manuscript. We have carefully addressed each of your suggestions/questions in the revised manuscript. Please see below for our responses to each comment below.

In addition, we think it is important to highlight the position of the Great Genotyper among the short-read genotypes. We believe GG is distinct from and complementary to the state-of-art single-sample genotypers. These genotypers like Pangenie and Paragraph are the best choice for genotyping a new sample for a defined list of variants. On the other hand, GG is best suited for:
1. Quick calculation of population allele frequency: This is typically needed after a genetic study discovering a variant in a set of samples. In this use case, accurate calculation of population allele frequency is very important to confirm the novelty and/or detecting population specificity of the variant. We added a new experiment to 3.1 section to calculate the population allele frequency of the outcome of 14 genomic studies. If the authors of these studies used a tool like Pangenie or Paragraph, they would have used the same computational resources 14 times. On the other hand, the GG needed minimal resources for genotyping once the index is built once.
One point that we would like to add here is that we used human data in this manuscript where benchmarking datasets are highly curated. However, given the limited genetic resources available for non-human species, our tool may be the only feasible option for such analyses.
2. Regenotyping a given population for new sets of variants which is needed in many situations including but not limited to:
a. Creating a new pangenome, adding more samples to the already existing one, or even changing the parameters of decomposing its bubbles would result in new VCFs with different variants.
b. A new tool of short read variant calling my results in detecting new set of variants
c. Variant calling of new samples in the population especially if using long read sequencing.

In this version of the manuscript, we made all the necessary updates to highlight these use-cases of GG as well as to make it clear to the readers that The Great Genotyper is not faster than single-sample tools in genotyping new samples for one set of variants.

Detailed response for each commont:

Comment 1:
What I was trying to say with my original comment was that I think cases where the variant set is fixed and the set of samples changes are much more common in practice (due to the ever increasing number of SRS datasets produced) than the N+1 case that the presented genotyper solves. In most use cases, genotyping happens just once per sample (possible adding additional samples afterwards). So in such a case, indexing the samples and running The Great Genotyper does not seem to be any faster than other tools (based on the provided runtimes for the indexing step, also see my response to Comment 2).

Response to Comment 1:
We hope that our earlier explanation in this rebuttal eliminates any confusion in the use cases of GG and how GG is distinct and has a complementary role to single-sample genotypers. To make the unique impact of GG clearer to the readers, we add a new experiment in section 3.3.1. This experiment is meant to highlight why the need for a tool to solve the N+1 cases is critical. We selected 14 recent human genetic studies that presented 790 likely pathogenic variants. With the efficiency of GG in re-genotyping, we were able to calculate the population allele frequency of most of these variants with high confidence. We believe this experiment shows the unique position of GG among other genotypes. The results were added to the manuscript at lines 205 -210. As well as Supplementary figure 13 and Supplementary table 3

We agree with the reviewer that The Great Genotyper is not faster than single-sample tools in genotyping a given set of samples for a defined set of variants once. We made several changes to the text throughout the manuscript to highlight this fact (See below in the comment addressing the benchmarking)

Comment 2:
Maybe I misunderstood the experiments presented but it is unclear to me why they demonstrate such a N+1 case. It seems that for the experiments described in 3.3.2, 3.3.3 and 3.3.4 the HPRC variants are genotyped in all 4k samples only one single time and all downstream analyses happen on the resulting genotypes. Or are additional runs of genotyping necessary to conduct the analyses described in these three paragraphs?

Response to Comment 2:
All the experiments described in 3.3.2, 3.3.3 and 3.3.4 genotyped the HPRC variants in the 4k samples once. However, these experiments are meant to show how easily resources like our 4k panel can be developed using the GG index for any new set of variants. Also, these experiments prove how reliable and useful these resources are. Given the increase in the number of pangenomes created since the HPRC, representing different ethnicities, and the increase in samples in the HPRC, as well as the expected pangenomes for non-human species, GG will enable easy creation and update of similar resources.

Comment 3:
The authors made some adjustments and provided more information on the resources needed for the indexing step. Given these numbers however, Figure 2A and the following statements made in the abstract (line 23) and Section 3.2 (line 159) are misleading:

"It took 100 hours to genotype 4.5M variants in the 4.2K samples using one server with 32 cores and 145GB of memory. A similar task would take months or even years using single-sample genotypers."

"To put this performance into context, Pangenie and GraphTyper2 required nearly an hour and 12 hours, respectively, to genotype the same 4.5 million variants in a single sample using the same machine. Extrapolating this duration, these state-of-the-art genotypers would take months to complete the genotyping process each time a new variant dataset needs analysis."

In order to provide a proper comparison of runtimes to justify these statements, (single-core) CPU times should be provided of all steps (preprocessing/indexing/genotyping) to reflect the total time needed by each tool from raw reads to genotypes. I understand that the index construction has to happen only once and does not need to be repeated when re-genotyping the cohort, but it still has to happen once, so the runtime cannot be ignored.

The numbers that the authors added still make a comparison between the tools difficult, since each step / tool was run on different machines with different numbers of cores. Given the currently provided runtimes, it seems that preprocessing/indexing took around 21 * 24 * 16 * 16 = 129024 single-core hours, subindexing took 10 * 24 * 4 * 32 = 30720 hours and genotyping 3200 hours. So given this, the total runtime is 162944 hours which is equal to 6789 days. PanGenie however needs 1 * 32 * 4200 = 134400 hours, which is 5600 days. So if this estimate is accurate, The Great Genotyper would actually be slower than PanGenie for genotyping a set of variants once across a cohort (the experiment described here). Only with more rounds of genotyping (on the same index), it would eventually be faster compared to genotyping the same set multiple times with PanGenie, since an additional genotyping step with The Great Genotyper would only add around 3200 hours, while the total time needed
for PanGenie doubles (2*134400 hours). However, the text as it is written now (as well as Figure 2A), creates the impression that even the first round is faster already, which, from the numbers currently provided, does not seem to be true.

Since speed is one of the selling points of this method, it is important that the authors provide a clear and detailed comparison of performances of the tools and quantify how big the speedup is for multiple rounds of genotyping on the same index. One way to do this would be to show total (single-core) runtimes for all tools for 1,2,3, … rounds of genotyping a fixed set of samples. While the first round (which requires indexing) would be comparable to the other tools (assuming my calculations above are correct), additional rounds of genotyping would demonstrate a big speedup as the indexing step can be skipped for The Great Genotyper, while the other tools need to start from scratch.

Response to Comment 3:
Unlike single-sample genotypers, GG is made for population allele frequency estimation and regenotyping scenarios. Therefore, benchmarking design is very challenging. To design a benchmarking experiment for the capabilities of GG, we need to assume scenarios matching its use-cases. For example, in the new experiment we added in this rebuttal, we genotyped the likely pathogenic variants identified in 14 studies. If we benchmarked this scenario, we should multiply the resources needed by single-sample genotypers by 14 (which is similar to the suggestion of the reviewer). However, we think this is unfair and unrealistic because these genotypers were never meant to address this scenario. Therefore, we see that the separation of computational assessment of indexing and genotyping is more fair and practical. Because users can have proper computational estimation based on their needs and their scenario of analysis.

However, we agree with the reviewer that this message should be clear to the users. Also, we agree that it is important to highlight that GG with the indexing step is not faster than single-sample tools in genotyping a given set of samples for a defined set of variants. Therefore, we addressed all the suggested points of confusion:

1. We updated the legend of Fig2A to indicate that the computational resources of indexing is not a part of the compression: "The Great Genotyper is currently genotyping 4,200 samples at 30x coverage assuming the indexing of the samples was already built"
2. we removed the line mentioned by the reviewer from the abstract at line 23: "A similar task would take months or even years using single-sample genotypers"
3. We believe that the statement quoted from Section 3.2 (line 159) has a clear context. For example, the preceding sentence clearly indicates that the resources included for the index were excluded "Once the indexing of the 4.2K WGS samples was done". Also, the statement itself says that these resources are required for "a new variant dataset". However, we added an extra sentence to this paragraph to avoid any misleading conclusions by the readers: "However, having the computational cost of creating the index, The Great Genotyper is not faster than single-sample genotypers in genotyping a given dataset only once."

In addition, we further updated our first paragraph of discussion to explain the scenarios that best fit GG and made a clear distinction from single-sample genotypes (lines 288-289):
"Therefore, the Great Genotyper is best suited for regenotyping scenarios which allow for quick calculation of population allele frequencies for any new set of variants. Also, it enables instant genotyping of the same dataset samples for any new set of variants. With this design, genotyping a large population once for a given set of variants using The Great Genotyper is still computationally on par with leading state-of-the-art genotyping tools. However, thanks to its pre-built index, re-genotyping the same cohort for any new set of variants becomes a much easier computational task."

Comment 4:
Thank you for adding the SV results for the 1kGP panel. I agree with the authors that the lower performance observed for SVs is expected, but I am still surprised that it is this low for 1KG Panel, especially compared to Manta, given that the ground truth are GIAB callsets which I assume mainly cover regions well accessible by short reads. Why are there so many variants covered by Manta that could not be imputed with the 1KG Panel? Where are these missing variants located?

Response to Comment 4:
As mentioned by the reviewer, the ground truth are GIAB callset which covers regions well accessible by short reads. This actually explains the high performance of menta which requires good alignment of short reads to achieve its best performance.

Regarding the 1KG Panel, we believe its performance as an imputation panel is low because of a couple reasons. First, the panel is missing many structural variants that we were able to identify in the pangenomes because the SVs in this panel were detected by short-read variant calling then were likely subjected to aggressive filtration to keep high quality SVs only. Second, proper imputation requires representations of all possible haplotypes which seems to be lacking from the 1KG panel because they are not performing well at the multiallelic sites which have a crucial role in imputation. These points are highlighted in the results (lines 236–243) and supported by Supplementary Figure 12.

Editorial Manager®

Cookie Preference Center

We use cookies which are necessary to make our site work. We may also use additional cookies to analyze, improve and personalize our content and your digital experience. For more information, see our Cookie Policy.

You may choose not to allow some types of cookies. However, blocking some types may impact your experience of our site and the services we are able to offer. See the different category headings below to find out more or change your settings.

Allow all

Manage Consent Preferences

Strictly Necessary Cookies

Always active

These cookies are necessary for the website to function and cannot be switched off in our systems. They are usually only set in response to actions made by you which amount to a request for services, such as setting your privacy preferences, logging in or filling in forms. You can set your browser to block or alert you about these cookies, but some parts of the site will not then work.

Functional Cookies

These cookies enable the website to provide enhanced functionality and personalisation. They may be set by us or by third party providers whose services we have added to our pages. If you do not allow these cookies then some or all of these services may not function properly.

Performance Cookies

These cookies allow us to count visits and traffic sources so we can measure and improve the performance of our site. They help us to know which pages are the most and least popular and see how visitors move around the site.

Targeting Cookies

These cookies may be set through our site by our advertising partners. They may be used by those companies to build a profile of your interests and show you relevant adverts on other sites. If you do not allow these cookies, you will experience less targeted advertising.

Cookie List

Clear

Apply Cancel

Consent Leg.Interest

Confirm my choices

Powered by onetrust
