# Supplementary material for: The Great Genotyper: a graph-based method for population genotyping of small and structural variants
Source: Gigascience. 2025 Oct 3;14:giaf112. doi: 10.1093/gigascience/giaf112 (PMC12491952; doi:10.1093/gigascience/giaf112)
Supplement: giaf112_Authors_Response_To_Reviewer_Comments_Revision_2 [file giaf112_authors_response_to_reviewer_comments_revision_2.pdf]

Author's Response To Reviewer Comments

We would like to thank the editor and the reviewers for their constructive comments and valuable feedback throughout the review process. In this round, we have addressed the remaining issues based on the editorial guidance provided during our last communication with Dr. Hans Zauner.

A) CPU Times for All Genotypers
Following the suggestion to provide a breakdown of CPU times for all genotypers, we have included a new Supplementary Table 4. This table presents benchmarking results for the Great Genotyper, PanGenie, and GraphTyper, including details of their core algorithms, application steps (e.g., indexing and genotyping), and runtime metrics. In addition, we updated the relevant paragraph on page 4, line 156, to clarify that the initial indexing step in the Great Genotyper makes one-time genotyping slower than with the other tools, and that its speed advantage becomes evident only in repeated genotyping scenarios. The new table is now cited in that paragraph as well as on page 7, line 294.

B) Variants Called by Manta but Missing in the 1K Panel
As explained in our last communication, we are now sharing the Truvari analysis outputs for all three panels tested in our evaluation. The following changes were made to the manuscript:
• Page 6, line 234: We added a pointer for readers to refer to the 'Data and Code Availability' section.
• Page 9, line 371: We included the following text in the 'Data and Code Availability' section: "The VCF output files of Truvari's pairwise analysis comparing SVs on Chr22—imputed using the 4k reference panel, the 1000 Genomes panel, and SV calls from Manta—are available at: https://farm.cse.ucdavis.edu/~tahmed/truvari.tar.gz"
• Figure 3 caption (Panel D): We updated the description to: "Panel D shows the precision and recall of variant imputation using the 4k reference panel in comparison to imputation using the 1000 Genomes panel and SV calling using Manta."

We appreciate the opportunity to improve our manuscript and look forward to your feedback.

Editorial Manager®

Cookie Preference Center

We use cookies which are necessary to make our site work. We may also use additional cookies to analyze, improve and personalize our content and your digital experience. For more information, see our Cookie Policy.

You may choose not to allow some types of cookies. However, blocking some types may impact your experience of our site and the services we are able to offer. See the different category headings below to find out more or change your settings.

Allow all

Manage Consent Preferences

Strictly Necessary Cookies

Always active

These cookies are necessary for the website to function and cannot be switched off in our systems. They are usually only set in response to actions made by you which amount to a request for services, such as setting your privacy preferences, logging in or filling in forms. You can set your browser to block or alert you about these cookies, but some parts of the site will not then work.

Functional Cookies

These cookies enable the website to provide enhanced functionality and personalisation. They may be set by us or by third party providers whose services we have added to our pages. If you do not allow these cookies then some or all of these services may not function properly.

Performance Cookies

These cookies allow us to count visits and traffic sources so we can measure and improve the performance of our site. They help us to know which pages are the most and least popular and see how visitors move around the site.

Targeting Cookies

These cookies may be set through our site by our advertising partners. They may be used by those companies to build a profile of your interests and show you relevant adverts on other sites. If you do not allow these cookies, you will experience less targeted advertising.

Cookie List

Clear

Apply Cancel

Consent Leg.Interest

Confirm my choices

Powered by onetrust
