# Supplementary material for: The Great Genotyper: a graph-based method for population genotyping of small and structural variants
Source: Gigascience. 2025 Oct 3;14:giaf112. doi: 10.1093/gigascience/giaf112 (PMC12491952; doi:10.1093/gigascience/giaf112)
Supplement: giaf112_Authors_Response_To_Reviewer_Comments_Revision_3 [file giaf112_authors_response_to_reviewer_comments_revision_3.pdf]

Author's Response To Reviewer Comments

Thank you so much for the excellent news regarding the acceptance of our manuscript for publication. We are very pleased to hear this.

We appreciate the suggested formatting changes to integrate the data DOI and restructure the Data and Code Availability sections. We have reviewed these proposed changes and have two comments that we believe will further clarify and optimize the presentation of our data and code.

First, regarding the splitting of the "Data and Code Availability" section into dedicated source code and data availability sections, we propose that the workflows for use cases be included within the code section, while the indexes be placed in the data availability section. This approach would ensure that all shared code is consistently available on GitHub, and all data is accessible via the UC Davis server and the GigaDB repository, maintaining a clear distinction and logical organization for our readers.

Second, we have a query regarding the citation of the HPRC PanGenie results. The suggested pointer, [cite A], is currently associated with "TheGreatGenotyper_usecases," which are not hosted on the specified Zenodo repository. We believe the citation might have been intended to refer to the HPRC pangenome. Therefore, we suggest an alternative location for this citation handle to accurately reflect the data source.

Based on these considerations, we have drafted a slightly revised structure for this section, but we are happy to address any further edits

em Editorial Manager®

Cookie Preference Center

We use cookies which are necessary to make our site work. We may also use additional cookies to analyze, improve and personalize our content and your digital experience. For more information, see our Cookie Policy.

You may choose not to allow some types of cookies. However, blocking some types may impact your experience of our site and the services we are able to offer. See the different category headings below to find out more or change your settings.

Allow all

Manage Consent Preferences

Strictly Necessary Cookies

Always active

These cookies are necessary for the website to function and cannot be switched off in our systems. They are usually only set in response to actions made by you which amount to a request for services, such as setting your privacy preferences, logging in or filling in forms. You can set your browser to block or alert you about these cookies, but some parts of the site will not then work.

Functional Cookies

These cookies enable the website to provide enhanced functionality and personalisation. They may be set by us or by third party providers whose services we have added to our pages. If you do not allow these cookies then some or all of these services may not function properly.

Performance Cookies

These cookies allow us to count visits and traffic sources so we can measure and improve the performance of our site. They help us to know which pages are the most and least popular and see how visitors move around the site.

Targeting Cookies

These cookies may be set through our site by our advertising partners. They may be used by those companies to build a profile of your interests and show you relevant adverts on other sites. If you do not allow these cookies, you will experience less targeted advertising.

Cookie List

Clear

Apply Cancel

Consent Leg.Interest

Confirm my choices

Powered by onetrust
