# Supplementary material for: The Great Genotyper: a graph-based method for population genotyping of small and structural variants
Source: Gigascience. 2025 Oct 3;14:giaf112. doi: 10.1093/gigascience/giaf112 (PMC12491952; doi:10.1093/gigascience/giaf112)
Supplement: giaf112_Reviewer_1_Report_Original_Submission — Paul Horton -- 7/29/2024 [file giaf112_reviewer_1_report_original_submission.pdf]

Reviewer Report

Title: The Great Genotyper: A Graph-Based Method for Population Genotyping of Small and Structural Variants

Version: Original Submission **Date:** 7/29/2024

Reviewer name: Paul Horton

Reviewer Comments to Author:

The manuscript introduces "The Great Genotyper", which the authors describe as a workflow or pipelinefor population genotyping of small and structural variants.First a bit of a disclaimer; although I have recently published in the field ofpangenome related computational methods for genotyping, I cannot claim to be a real expert.In any case here is my review.SUMMARYThe application area is important.Several comparison experiments are reported, the results are clearly presented and seem impressive.Although I have not verified them, I have no reason to doubt their validity.However I am not completely sure what the main innovation of the manuscript is.Is it in applying existing tools in a novel combination, or in better parameter tuning ofthose tools, such as the parameter tuning of MetaGraph shown in Supplementary Fig. 7?Or are the comparison experiments described in the manuscript themselves the main innovation?I would like to see a more explicit summary of the innovations introduced by the authors,and a more clear positioning of this work and MetaGraph (see below for more on this).WRITINGthe English is clean and clear.AVAILABILITY OF SOFTWAREthe authors provide a link to a GitHub repository including source codeand instructions on how to install and run the pipeline.(I did not test it out, but it looks reasonable).REGARDING DESCRIPTION OF THE METHODOLOGYStarting with the abstract, the "Counting Colored De Bruijn Graph" (CCDG) data structure is mentionedseveral places in the manuscript without detailed explanation.In particular, just reading the Methods section alone does not make it completely clearthat CCDG here refers to the data structure employed by Metagraph [M Karasikov et al. 2020].In Results section 3.1, the authors do clearly state that Metagraph is used to construct CCDGs,but I would expect to find that information in the Methods section.Moreover it seems that perhaps Metagraph is rather central to this work(?)If so, then it should be given more prominence in the manuscript.Incidentally, "sourmash sketch" is another non-self-explanatory term which comes up in multiplespots in the manuscript and is partially explained in section 3.1 as a "FracMinHash"calculated by sourmash [cite] used "for quality control including calculation of sequencingdepth, ...". Other than this description belonging under methods, it is useful asfar as it goes, and yes the reader can refer to the citation for a detailed description.However as a reader I would have appreciated a line or two more giving a rough explanationof what a sourmash sketch or FracMinHash actually is.In general, much of the content of section 3.1 seems to be more suitably placed underMethods rather then Results.IMPORTANT BUT EASILY FIXEDIn the References section, all citations seem to be missing the year field.MINOR AND EASILY FIXED"Figure 3E summarizes of the associations"--> "Figure 3E summarizes the associations"Misspellings in Suppl. Fig. 3 legendDISCRETIONARY MINOR COMMENTSThe text does not directly refer to subfigure 3D.I think it depicts results described inSection 3.3.3 Impute SV by using the 4k reference panel.I would be more sure if the main text referred to

thatsubfigure at the appropriate spot.When reading the legend of Figure 3E, I did not know what a "Sankey plot" was;and whether is it a general category of plot or something specific to genomics.So an explanation or reference might be in order.On the other hand; Wikipedia does have entries for "Sankey plot" and "alluvial diagram",so I would leave this suggestion to the discretion of the authors.OTHER COMMENTSThe analysis of the human fibrinogen locus variant ClinVar:RCV000247066 seems likea nice anecdotal result.

Level of Interest

Please indicate how interesting you found the manuscript: Choose an item.

Quality of Written English

Please indicate the quality of language in the manuscript: Choose an item.

Declaration of Competing Interests

Please complete a declaration of competing interests, considering the following questions:

- Have you in the past five years received reimbursements, fees, funding, or salary from an organisation that may in any way gain or lose financially from the publication of this manuscript, either now or in the future?
- Do you hold any stocks or shares in an organisation that may in any way gain or lose financially from the publication of this manuscript, either now or in the future?
- Do you hold or are you currently applying for any patents relating to the content of the manuscript?
- Have you received reimbursements, fees, funding, or salary from an organization that holds or has applied for patents relating to the content of the manuscript?
- Do you have any other financial competing interests?
- Do you have any non-financial competing interests in relation to this paper?

If you can answer no to all of the above, write 'I declare that I have no competing interests' below. If your reply is yes to any, please give details below.

I declare that I have no competing interests

I agree to the open peer review policy of the journal. I understand that my name will be included on my report to the authors and, if the manuscript is accepted for publication, my named report including any attachments I upload will be posted on the website along with the authors' responses. I agree for my report to be made available under an Open Access Creative Commons CC-BY license (http://creativecommons.org/licenses/by/4.0/). I understand that any comments which I do not wish to

be included in my named report can be included as confidential comments to the editors, which will not be published.

Choose an item.

To further support our reviewers, we have joined with Publons, where you can gain additional credit to further highlight your hard work (see: https://publons.com/journal/530/gigascience). On publication of this paper, your review will be automatically added to Publons, you can then choose whether or not to claim your Publons credit. I understand this statement.

Yes Choose an item.
