# Supplementary material for: The Great Genotyper: a graph-based method for population genotyping of small and structural variants
Source: Gigascience. 2025 Oct 3;14:giaf112. doi: 10.1093/gigascience/giaf112 (PMC12491952; doi:10.1093/gigascience/giaf112)
Supplement: giaf112_Reviewer_2_Report_Original_Submission — Jana Ebler -- 8/6/2024 [file giaf112_reviewer_2_report_original_submission.pdf]

Reviewer Report

Title: The Great Genotyper: A Graph-Based Method for Population Genotyping of Small and Structural Variants

Version: Original Submission **Date:** 8/6/2024

Reviewer name: Jana Ebler

Reviewer Comments to Author:

In their paper "The Great Genotyper: A Graph-Based Method for Population Genotyping of Small and Structural Variants" the authors present a new genotyping tool called "The Great Genotyper" which can genotype small and structural variants from various sources based on short-read sequencing data. The method is specifically designed to solve the N+1 problem, i.e. rapidly genotyping new variants in a fixed set of short-read sequencing (SRS) datasets. The first step is the creation of an indexing structure which consists of a Counting Colored de Bruijn graph (CCDG). It offers a compact representation of the sequencing data sets of the population. This structure is used later for k-mer based genotyping. The authors show that their method performs as good as state-of-the-art tools, while being much faster in the N+1 scenario. The authors furthermore demonstrate several use cases of their tool, including the creation of a reference panel consisting of 4k samples, SV imputation and LD analysis.Overall, the study is well designed. The genotyping performance of the tool is properly benchmarked using other state-of-the-art tools for short reads, and the authors show how their tool can be used to gain new biological insights. However, I think the comparisons in terms of speed and memory are not fair since the computational resources required to construct the index are neither mentioned nor taken into account for the comparison. Also, I think the description of the underlying genotyping algorithm that was developed in this paper should be improved, as it is currently not very clear how the method works in detail.Major Comments:1) The genotyping workflow is designed specifically for cases where the set of SRS samples is fixed. While there might be applications for which this is the case, I think the much more frequent scenario is that the set of SRS samples is variable, as new SRS datasets are being produced continuously. Is it possible to efficiently update the CCDG in such cases, i.e. can new SRS datasets be efficiently added to an existing CCDG, or does the whole index need to be rebuilt in such cases? Furthermore, I think many real-world scenarios require building a CCDG from scratch, e.g. if one wants to genotype a special cohort of samples, samples sequenced with a specific coverage, newly sequenced samples, etc. In practice, I'm not sure if there are many cases that really benefit from the strength of the method (being able to reuse a precomputed index for a specific population). Therefore, I think it would be good to highlight such use cases more and give concrete examples to convince the reader why it is useful.2) In line 133 the authors state: "The Great Genotyper could genotype 4.5 million variants across 4.2K WGS samples in approximately 100 hours, utilizing 32 cores and 145 GB of memory, as depicted in Figure 2A. To put this performance into context, Pangenie and GraphTyper2 required nearly an hour and 12 hours, respectively, to genotype the same 4.5 million variants in a single sample using the same machine." From what I understand, these time / memory requirements do not include the time needed to construct the CCDG index structure. I think all three genotyping workflows should be compared based

on their runtime and memory usages starting from the raw reads, because this is what matters in practice. Currently, the resources needed to build the CCDG are not included. The authors must add the runtime and memory requirements of the indexing step on a single 32 core machine with 145 GB of RAM (as used for the other two tools) because this step has to be done, even if it is just once, so it should not be neglected.3) In addition to adding detailed time and memory usage of the indexing workflow (see comment above), how does the construction of the index and the genotyping scale in terms of runtime and memory usage for larger sets of SRS samples? And how does the size of the index scale? Would the workflow be applicable to large collections of SRS data, e.g. the UK biobank? What is the limit in terms of sample size?4) I think the benchmarking experiments were well designed, but some more details should be provided. How are precision/recall/F-score metrics computed exactly? Do these metrics evaluate the actual genotypes or just presence/absence signals? i.e. if a variant is genotyped as 0/1 while being homozygous (1/1) in the ground truth, would this error be counted? The versions of different tools used in the study should also be mentioned. Was the latest version of each genotyping tool used for the benchmarking experiments?5) In line 197 it is stated that no similar reference panel is available for SVs. There is in fact a more recent, improved reference panel available for the 1kg cohort which does include SVs as well, see: Byrska-Bishop et al, PMID: 36055201. How do the results compare to this panel?6) The description of how the genotyping algorithm works needs to be more elaborate, i.e. which/how components are used from other tools like PanGenie and Beagle, and which parts have been newly developed. How exactly do the steps work? I think the Methods section as it is right now is not detailed enough for the reader to understand how the method works. Also, more details on the evaluation metrics should be given (see my comment further above).7) Is the pipeline to generate the index CCDG publicly available? The code provided here https://github.com/dib-lab/TheGreatGenotyper/tree/master seems to only contain the genotyper. Furthermore, documentation and instructions on how to construct indexes need to be provided.Minor Comments:1) Line 180: the HPRC citation is missing2) Line 345: Pangenine -> Pangenie (typo)

Level of Interest

Please indicate how interesting you found the manuscript: Choose an item.

Quality of Written English

Please indicate the quality of language in the manuscript: Choose an item.

Declaration of Competing Interests

Please complete a declaration of competing interests, considering the following questions:

- Have you in the past five years received reimbursements, fees, funding, or salary from an organisation that may in any way gain or lose financially from the publication of this manuscript, either now or in the future?

- Do you hold any stocks or shares in an organisation that may in any way gain or lose financially from the publication of this manuscript, either now or in the future?
- Do you hold or are you currently applying for any patents relating to the content of the manuscript?
- Have you received reimbursements, fees, funding, or salary from an organization that holds or has applied for patents relating to the content of the manuscript?
- Do you have any other financial competing interests?
- Do you have any non-financial competing interests in relation to this paper?

If you can answer no to all of the above, write 'I declare that I have no competing interests' below. If your reply is yes to any, please give details below.

1. No 2. No 3. No 4. No 5. No 6. No, but note that I am the author of PanGenie, a method that the tool presented in this paper built upon and is compared to.

I agree to the open peer review policy of the journal. I understand that my name will be included on my report to the authors and, if the manuscript is accepted for publication, my named report including any attachments I upload will be posted on the website along with the authors' responses. I agree for my report to be made available under an Open Access Creative Commons CC-BY license (http://creativecommons.org/licenses/by/4.0/). I understand that any comments which I do not wish to be included in my named report can be included as confidential comments to the editors, which will not be published.

Choose an item.

To further support our reviewers, we have joined with Publons, where you can gain additional credit to further highlight your hard work (see: https://publons.com/journal/530/gigascience). On publication of this paper, your review will be automatically added to Publons, you can then choose whether or not to claim your Publons credit. I understand this statement.

Yes Choose an item.
