# Supplementary material for: The Great Genotyper: a graph-based method for population genotyping of small and structural variants
Source: Gigascience. 2025 Oct 3;14:giaf112. doi: 10.1093/gigascience/giaf112 (PMC12491952; doi:10.1093/gigascience/giaf112)
Supplement: giaf112_Reviewer_2_Report_Revision_1 — Jana Ebler -- 1/15/2025 [file giaf112_reviewer_2_report_revision_1.pdf]

Reviewer Report

Title: The Great Genotyper: A Graph-Based Method for Population Genotyping of Small and Structural Variants

Version: Revision 1 **Date:** 1/15/2025

Reviewer name: Jana Ebler

Reviewer Comments to Author:

Re Comment 1:What I was trying to say with my original comment was that I think cases where the variant set is fixed and the set of samples changes are much more common in practice (due to the ever increasing number of SRS datasets produced) than the N+1 case that the presented genotyper solves. In most use cases, genotyping happens just once per sample (possible adding additional samples afterwards). So in such a case, indexing the samples and running The Great Genotyper does not seem to be any faster than other tools (based on the provided runtimes for the indexing step, also see my response to Comment 2). Maybe I misunderstood the experiments presented but it is unclear to me why they demonstrate such a N+1 case. It seems that for the experiments described in 3.3.2, 3.3.3 and 3.3.4 the HPRC variants are genotyped in all 4k samples only one single time and all downstream analyses happen on the resulting genotypes. Or are additional runs of genotyping necessary to conduct the analyses described in these three paragraphs?Re Comment 2:The authors made some adjustments and provided more information on the resources needed for the indexing step. Given these numbers however, Figure 2A and the following statements made in the abstract (line 23) and Section 3.2 (line 159) are misleading:"It took 100 hours to genotype 4.5M variants in the 4.2K samples using one server with 32 cores and 145GB of memory. A similar task would take months or even years using single-sample genotypers.""To put this performance into context, Pangenie and GraphTyper2 required nearly an hour and 12 hours, respectively, to genotype the same 4.5 million variants in a single sample using the same machine. Extrapolating this duration, these state-of-the-art genotypers would take months to complete the genotyping process each time a new variant dataset needs analysis."In order to provide a proper comparison of runtimes to justify these statements, (single-core) CPU times should be provided of all steps (preprocessing/indexing/genotyping) to reflect the total time needed by each tool from raw reads to genotypes. I understand that the index construction has to happen only once and does not need to be repeated when re-genotyping the cohort, but it still has to happen once, so the runtime cannot be ignored.The numbers that the authors added still make a comparison between the tools difficult, since each step / tool was run on different machines with different numbers of cores. Given the currently provided runtimes, it seems that preprocessing/indexing took around 21 * 24 * 16 * 16 = 129024 single-core hours, subindexing took 10 * 24 * 4 * 32 = 30720 hours and genotyping 3200 hours. So given this, the total runtime is 162944 hours which is equal to 6789 days. PanGenie however needs 1 * 32 * 4200 = 134400 hours, which is 5600 days. So if this estimate is accurate, The Great Genotyper would actually be slower than PanGenie for genotyping a set of variants once across a cohort (the experiment described here). Only with more rounds of genotyping (on the same index), it would eventually be faster compared to genotyping the same set multiple times with PanGenie, since an

additional genotyping step with The Great Genotyper would only add around 3200 hours, while the total time needed for PanGenie doubles (2*134400 hours). However, the text as it is written now (as well as Figure 2A), creates the impression that even the first round is faster already, which, from the numbers currently provided, does not seem to be true.Since speed is one of the selling points of this method, it is important that the authors provide a clear and detailed comparison of performances of the tools and quantify how big the speedup is for multiple rounds of genotyping on the same index. One way to do this would be to show total (single-core) runtimes for all tools for 1,2,3, … rounds of genotyping a fixed set of samples. While the first round (which requires indexing) would be comparable to the other tools (assuming my calculations above are correct), additional rounds of genotyping would demonstrate a big speedup as the indexing step can be skipped for The Great Genotyper, while the other tools need to start from scratch.Re Comment 3: OKRe Comment 4: OKRe Comment 5:Thank you for adding the SV results for the 1kGP panel. I agree with the authors that the lower performance observed for SVs is expected, but I am still surprised that it is this low for 1KG Panel, especially compared to Manta, given that the ground truth are GIAB callsets which I assume mainly cover regions well accessible by short reads. Why are there so many variants covered by Manta that could not be imputed with the 1KG Panel? Where are these missing variants located?Re Comment 6: OKRe Comment 7: OKRe Comment 8: OK

Level of Interest

Please indicate how interesting you found the manuscript: Choose an item.

Quality of Written English

Please indicate the quality of language in the manuscript: Choose an item.

Declaration of Competing Interests

Please complete a declaration of competing interests, considering the following questions:

- Have you in the past five years received reimbursements, fees, funding, or salary from an organisation that may in any way gain or lose financially from the publication of this manuscript, either now or in the future?
- Do you hold any stocks or shares in an organisation that may in any way gain or lose financially from the publication of this manuscript, either now or in the future?
- Do you hold or are you currently applying for any patents relating to the content of the manuscript?
- Have you received reimbursements, fees, funding, or salary from an organization that holds or has applied for patents relating to the content of the manuscript?
- Do you have any other financial competing interests?
- Do you have any non-financial competing interests in relation to this paper?

If you can answer no to all of the above, write 'I declare that I have no competing interests' below. If your reply is yes to any, please give details below.

1. no 2. no 3. no 4. no 5. no 6. no - note that I am the author of PanGenie, one of the tools that the presented method builds upon.

I agree to the open peer review policy of the journal. I understand that my name will be included on my report to the authors and, if the manuscript is accepted for publication, my named report including any attachments I upload will be posted on the website along with the authors' responses. I agree for my report to be made available under an Open Access Creative Commons CC-BY license (http://creativecommons.org/licenses/by/4.0/). I understand that any comments which I do not wish to be included in my named report can be included as confidential comments to the editors, which will not be published.

Choose an item.

To further support our reviewers, we have joined with Publons, where you can gain additional credit to further highlight your hard work (see: https://publons.com/journal/530/gigascience). On publication of this paper, your review will be automatically added to Publons, you can then choose whether or not to claim your Publons credit. I understand this statement.

Yes Choose an item.
