# Supplementary material for: The Great Genotyper: a graph-based method for population genotyping of small and structural variants
Source: Gigascience. 2025 Oct 3;14:giaf112. doi: 10.1093/gigascience/giaf112 (PMC12491952; doi:10.1093/gigascience/giaf112)
Supplement: giaf112_Reviewer_2_Report_Revision_2 — Jana Ebler -- 5/7/2025 [file giaf112_reviewer_2_report_revision_2.pdf]

Reviewer Report

Title: The Great Genotyper: A Graph-Based Method for Population Genotyping of Small and Structural Variants

Version: Revision 2 **Date:** 5/7/2025

Reviewer name: Jana Ebler

Reviewer Comments to Author:

Unfortunately, many of my comments have not been sufficiently addressed. In particular:Comment 3:My previous comment regarding CPU times has not been addressed. The authors must provide CPU times for all tools (The Great Genotyper, PanGenie and Paragraph) for 1,2,3 .. rounds of genotyping. The authors argue that the other tools were designed for a different scenario. But this is exactly the point to show here - that your tool solves a problem that the other tools were not able to do efficiently. Also, as I mentioned several times before, the resources of the indexing step must not be ignored as this is a step that is absolutely necessary to use the tool. Even if it just happens once, in practice, users want to know how which resources are necessary to run a tool.Comment 4:My comments were not addressed by the authors. My question was why there were so many variants covered my Manta (a short-read caller) but not in the 1kg panel (also a short read callset). I was also asking where these missing variants are located. These comments must be addressed.

Level of Interest

Please indicate how interesting you found the manuscript: Choose an item.

Quality of Written English

Please indicate the quality of language in the manuscript: Choose an item.

Declaration of Competing Interests

Please complete a declaration of competing interests, considering the following questions:

- Have you in the past five years received reimbursements, fees, funding, or salary from an organisation that may in any way gain or lose financially from the publication of this manuscript, either now or in the future?
- Do you hold any stocks or shares in an organisation that may in any way gain or lose financially from the publication of this manuscript, either now or in the future?
- Do you hold or are you currently applying for any patents relating to the content of the manuscript?
- Have you received reimbursements, fees, funding, or salary from an organization that holds or has applied for patents relating to the content of the manuscript?

- Do you have any other financial competing interests?
- Do you have any non-financial competing interests in relation to this paper?

If you can answer no to all of the above, write 'I declare that I have no competing interests' below. If your reply is yes to any, please give details below.

1. No 2. No 3. No 4. No 5. No 6. No. But note I'm the author of PanGenie, a tool that the authors compare to.

I agree to the open peer review policy of the journal. I understand that my name will be included on my report to the authors and, if the manuscript is accepted for publication, my named report including any attachments I upload will be posted on the website along with the authors' responses. I agree for my report to be made available under an Open Access Creative Commons CC-BY license (http://creativecommons.org/licenses/by/4.0/). I understand that any comments which I do not wish to be included in my named report can be included as confidential comments to the editors, which will not be published.

Choose an item.

To further support our reviewers, we have joined with Publons, where you can gain additional credit to further highlight your hard work (see: https://publons.com/journal/530/gigascience). On publication of this paper, your review will be automatically added to Publons, you can then choose whether or not to claim your Publons credit. I understand this statement.

Yes Choose an item.
